# Supplementary material for: The Miocene primate Pliobates is a pliopithecoid
Source: Nat Commun. 2024 Apr 1;15:2822. doi: 10.1038/s41467-024-47034-9 (PMC10984959; doi:10.1038/s41467-024-47034-9)
Supplement: Supplementary file 1 — Supplementary Information [file 41467_2024_47034_MOESM1_ESM.pdf]

Supplementary Information

The Miocene primate *Pliobates* is a pliopithecoid

Florian Bouchet*, Clément Zanolli, Alessandro Urciuoli, Sergio Almécija, Josep Fortuny, Josep M. Robles, Amélie Beaudet, Salvador Moyà-Solà, David M. Alba*

*To whom correspondence may be addressed: florian.bouchet@icp.cat, david.alba@icp.cat

This file includes:

Supplementary Text 1 to 4

Supplementary Figure 1 to 9

Supplementary Table 1 to 6

Supplementary References

Supplementary Text 1. Differential diagnosis (Differential diagnosis relative to other pliopithecoids emended from the original description based on dental features; for cranial and postcranial features, see ref. 1). *Pliobates* differs from non-crouzeliid pliopithecoids in the relatively narrower P4 with a narrower distal basin; the more buccolingually compressed cusps and sharper crests in the molars; the M1 and M2 markedly longer buccally than lingually; the relatively narrower M2; the relatively broader and more trapezoidal M3, with a lingually located metacone and a lesser developed lingual cingulum; the more inclined i2; the relatively narrower m1 and m2, with a longer mesial fovea, a distal fovea continuous with the talonid basin (more clearly so in the m1), and the hypoprotocristid originating distally from the protoconid; and the lack of the mesial arm of the pliopithecine triangle in both m1 and m2 (although it is only variably present and poorly developed in *Epipliopithecus*). It also differs from dionysopithecids in the more asymmetrical I1 (unknown in *Platodontopithecus*); the more mesial paracone in the upper premolars; the upper molars with a more distally located protocone and a smaller hypocone; the less developed lingual cingulum distally to the hypocone in M1 and M2; the lesser developed buccal cingulum in M3; the relatively broader P3 with a less peripheral paracone (unknown in *Dionysopithecus*); and the less peripheralized cusps of the lower molars. *Pliobates* further differs from pliopithecids in the more asymmetrical I1; the more ovoid and relatively narrower P3 with a narrower distal basin and a more peripheral paracone; the relatively narrower M1 with a relatively longer trigon basin; the more distally located protocone and the smaller hypocone in M3; the female c1, with a more marked mesiolingual cuspulid-like enamel thickening (only known in *Pliopithecus platyodon*). Finally, *Pliobates* also differs from *Epipliopithecus* in the m1 and m2 with a more medial hypoconulid and a very distinct distal arm of the pliopithecine triangle.

In turn, *Pliobates* differs from other crouzeliid genera in the more suboval p4 with more mesial metaconid and protoconid (unknown in *Krishnapithecus*), and the m1 and m2 with entoconid almost transversely aligned with the hypoconid (except *Barberapithecus*) and lacking the mesial arm of the pliopithecine triangle (although it is variably present in *Anapithecus* and absent to only incipiently developed in *Laccopithecus* and *Krishnapithecus*). It also differs from anapithecines in the more ovoid P3 with a less peripheral paracone (unknown in *Krishnapithecus*); the relatively broader and more trapezoidal M3, with less peripheral metacone and smaller hypocone (unknown in *Egarapithecus*); and the relatively narrower m1 and m2. From anapithecines other than *Fanchangia*, in the smaller M2–M3 metacone; the lack of distinct p4 distal cuspids; and the presence of a distinct distal arm of

the pliopithecine triangle in both m1 and m2 (variably present in *Anapithecus*). From anapithecines other than *Anapithecus*, in the better developed (more ledge-like) buccal cingulid in lower molars. From anapithecines other than *Krishnapithecus*, in the distal fovea of lower molars, continuous with the talonid basin (more clearly so in m1). From *Fanchangia*, *Krishnapithecus*, and *Laccopithecus*, in the more buccolingually compressed cusps and sharper crests in the molars; and the m1 and m2 with a longer mesial fovea and hypoprotocristid originating distally from the protoconid. From *Fanchangia*, *Laccopithecus*, and *Anapithecus*, in the more asymmetrical I1, with narrower cingulum and slenderer crown base; the more suboval and relatively broader P4; the less buccolingually waisted M1 and M2; and the relatively broader female c1. From *Fanchangia* and *Laccopithecus*, in the presence of a rudimentary hypocone in the P4; and the broader lingual cingulum in M1 and M2. From *Laccopithecus* and *Anapithecus*, in the lesser developed lingual cingulum in M3. From *Anapithecus* and *Egarapithecus*, in the less inclined cristid obliqua in m1 and m2 (directed toward the protoconid instead of the hypoprotocristid-hypometacristid merging). From *Krishnapithecus*, in the lower, less peripheral, but more extensive cuspids in m1 and m2, including a larger hypoconulid with distinct postcristid and hypoentocristid. From *Anapithecus* in the mesiodistally waisted i2 towards cervix. And from *Egarapithecus*, in the more medial hypoconulid in m1 and m2.

Finally, *Pliobates* differs from other crouzeliine genera (*Barberapithecus*, *Plesiopliopithecus*, and *Crouzelia*) in a unique combination of dental features. From *Barberapithecus* and *Plesiopliopithecus*, in the distal fovea continuous with the talonid basin in the lower molars (especially m1). From *Barberapithecus* and *Crouzelia*, in the wider buccal cingulid in m1 and m2. From *Barberapithecus*, in the more asymmetrical I1; the upper molars with narrower buccal cingulum and more peripheral buccal cusps; the M1 and M2 with wider lingual cingulum and less buccolingual waisting; the relatively broader and more trapezoidal M3, with more lingually located metacone, smaller hypocone, and narrower lingual cingulum; the female c1 with a more marked mesiolingual cuspulid-like thickening; and the relatively narrower m1 and m2, with hypoprotocristid originating more distally from the protoconid. From *Plesiopliopithecus* and *Crouzelia*, in the lesser developed buccal cingulid in p4; and the m1 and m2 with larger hypoconulid, and a more distinct distal arm of the pliopithecine triangle. Finally, from *Crouzelia*, in the distinct postcristid and hypoentocristid in m1 and m2.

Supplementary Text 2. Preservation of the specimens The dental remains of the holotype are preserved in two different maxillary fragments (Fig. 1 in ref. 1): IPS58443.1 includes the right I1–C1 alveoli filled with sediment, the broken P3, and the complete P4–M3 series (Fig. 2m); in turn, IPS58443.2 (Fig. 2m) preserves the left M3, the broken M2 (the buccal moiety is broken away), and the apicalmost portion of the lingual and distobuccal roots of the M1. The molars of the holotype show an advanced (M1–M2) to moderately advanced (M3) degree of wear. In the M1, the four main cusps have been completely eroded by wear (the dentine exposures corresponding to the paracone and metacone being continuous with one another). The M2 shows a comparable degree of wear, except for the metacone, which is only worn at its tip, with its dentine exposed area not being continuous with that of the paracone. The M3 shows a more moderate degree of wear, with dentine exposure being limited to the protocone and the apex of the paracone.

IPS44014 (Figs. 2a–c) is a left maxillary fragment preserving the I1–I2 and C1 alveoli (the latter somewhat damaged distally) as well as the socketed P3–M1 series. The P3 and P4 crowns are well preserved and only show slight wear with minimal dentine exposure restricted to the paracone apex. The M1 is only slightly more worn than the premolars, with dentine exposure limited to the apices of its four main cusps.

The right maxillary fragment IPS43758 (Fig. 2d–e) preserves the M1–M2, which show an advanced degree of wear (even more marked in the M2 than in the M1). The M1 further has its mesiolingual portion slightly damaged (a fragment of the enamel cap is missing), and the M2 is more severely damaged on its distal portion, since a crown fragment is missing, although measurements can be readily taken. There is abundant dentine exposure in the four main cusps, with the exposed areas corresponding to the protocone and hypocone completely merged in the M2. The M1 also shows dentine exposure at the metacone. Owing to bone breakage, the roots are visible.

The left maxillary fragment IPS42977 (Figs. 2j–l) preserves an almost complete DP4 that is only missing a chip of enamel from the distolingual portion of the crown wall. It shows some dentine exposure along the crista obliqua and the apices of the three cusps.

The left I1 IPS43488 (Fig. 2z) is completely preserved except for the apical end of the root. The crown is very worn, both labially and, more markedly, lingually, so that dentine is abundantly exposed along the whole distolingual portion of the crown (from the incisal edge to close to the base) and its mesiolingual basal portion.

The left M2 germ IPS94888 (Figs. 2q and 4i) is very well preserved and further includes the basalmost portion of the forming roots.

The right M1 IPS100379 (Figs. 2p and 4g) preserves the crown, which is very corroded, as well as the complete lingual root. The occlusal surface is covered by a carbonaceous crust that extends onto the root. The cusp apices show a moderate degree of wear. In contrast, most of the lingual, mesial, and distal parts of the crown completely expose the dentine exposure, probably because the enamel cap has been dissolved.

The left M2 germ IPS100384 (Figs. 2r and 4h) preserves the unworn occlusal morphology.

The mandibular fragment IPS43936 (Figs. 1g–l and 3) includes the symphysis and the left corpus. The alveoli of the left di1–dc1 and right di1 are preserved. The crown of the dp3 is only partially preserved (since its distalmost portion is missing) and shows very slight wear with no dentine exposure (Figs. 2x and 4o). The crown of the dp4 is well preserved, except for a small enamel chip missing from the mesial aspect of the metaconid, and only shows minimal wear on the cuspid apices (Figs. 2x and 4p). The occlusal morphology of the m1 is perfectly preserved because it is an unerupted germ, but it can only be partially ascertained because it is still inside its crypt. 3D models of the m1 and the m2 germs could nevertheless be digitally extracted from the CT scans (Figs. 3 h–i and 4k–l), that of the m2 lacking a larger portion of the crown base, although reliable estimates of BL and MD measurements could be taken. 3D models of tooth germs of the right i1 and left i1–p4 were similarly extracted from their crypts (Fig. 3c–g). While the i1 crowns are nearly completed down to the cervix and show a beginning of root formation, the i2 and c1 crowns are close to completion, but lack the basalmost portion of the crown. The crown germs of p3–p4 only show parts of the cusps. Due to the lack of completion of enamel formation, no measurements were taken.

The left i2 IPS44273 (Fig. 2a’) preserves the whole crown and most of the root. The crown is moderately worn with some dentine exposure along the incisal edge.

The left c1 IPS43433 (Fig. 2b’) is completely preserved. The crown displays a moderate degree of wear at its apex and more abundant dentine exposure on the distobuccal basal portion of the crown.

The right p4 IPS43820 (Fig. 2t) is very fragmentary, consisting of a very worn partial crown that is missing its mesiolingual portion and only preserves the basalmost portion of the roots. Most occlusal details have been worn away.

The right p4 IPS93524 (Figs. 2s and 4j) is only partially preserved, including the damaged crown and the basalmost portion of the roots. Most of the mesial end of the crown is broken away, and enamel is also missing from the distal end and the buccal margin of the talonid basin. The preserved occlusal portions display an advanced degree of wear, with dentine exposure at the metaconid apex.

The right m2 unworn germ IPS94886 (Figs. 2u and 4m) only preserves the mesial part of the crown, including the protoconid, the metaconid, and the trigonid basin.

The right DI1 IPS44393 (Fig. 2z) is an unworn germ.

The left DP3 germ IPS43013 (Fig. 2v) displays a moderate degree of wear, mainly restricted to the three main cusps, with minimal dentine exposure, and to the distal portion of the crown.

The left dp4 IPS106878 crown (Figs. 2m and 3q) is overall well preserved, showing minimal to slight wear on the apices of the five main cuspids. However, there is an oblique crack running from the hypoconid to the mesiolingualmost corner of the crown, revealing some dentine along the cristid obliqua. The roots of this tooth seem to have been broken away.

Supplementary Text 3. Extended Descriptions

Upper incisors As preserved, the crown of the left I1 IPS43488 displays a subovoid occlusal contour that is tilted mesiolabially relative to its main mesiodistal axis (Fig. 2z). The crown is spatulate and waisted at the cervix, with an inclined incisal edge (the crown being higher mesially than distally). It is broader than long (although mesiodistal length is probably affected by wear) and moderately high (the preserved labial height of 4.7 mm is only slightly greater than occlusal dimensions). The cervix displays an inverted V-shape distolabially and especially mesiolabially. On the mesial aspect of the crown, there is an interproximal wear facet against the right I1 close to the incisal edge. Lingual morphology cannot be adequately ascertained due to wear, but the distal ridge appears shorter than the mesial, which displays a marked angulation at about midheight. The root is mesiodistally compressed (MD = 2.9 mm, BL = 4.4 mm) and somewhat curved distalward, with a marked constriction toward the cervix. The I1 alveoli of both the holotype (Fig. 1 in ref. 1) and IPS44014 (Fig. 2a–c) suggest that this tooth was slightly procumbent.

The I2 alveoli of the holotype (MD = 3.0 mm, BL = 4.0 mm) and of IPS44014 (MD = 2.2 mm, BL = 3.4 mm) suggest that this incisor displayed a somewhat mesiodistally compressed root and was more vertically and distally socketed than the I1 (Fig. 1 in ref. 1; Fig. 2a–c). The dimensions of their alveoli indicate some degree of incisor heteromorphy.

Upper canine No C1 is available, but its alveolus is preserved in two specimens (Fig. 1 in ref. 1; Fig. 2a–c), displaying an elliptical occlusal contour that is longer than broad, with its main mesiodistal axis being slightly exorotated. The C1 alveolus of the holotype (MD = 4.9, BL = 3.8 mm) is much smaller than that of IPS44014 (MD = 8.6 mm, BL = 5.6 mm), being respectively attributed to female and male individuals, and thus hinting at a marked degree of canine sexual dimorphism. In both specimens, the C1 and the I2 are separated by small diastema (1.8 mm in the holotype and 1.3 mm in IPS44014). The postcanine tooth row slightly diverges distalward from the sagittal plane.

Upper premolars The P3 occlusal morphology cannot be adequately ascertained from the holotype due to damage (Fig. 2m) but is well preserved in IPS44014 (Figs. 2n and 4c). The crown displays a suboval contour much broader than long (BLI = 145%) and slightly longer buccally than lingually, with a more markedly convex profile on the lingual side. The two main cusps are buccolingually compressed to some extent (particularly the paracone, which is taller and more extensive than the protocone). There is considerable buccal flare, with the crown base appearing inflated due to the non-

peripheral position of the paracone, but the buccal cingulum is very narrow (barely distinguishable except at its mesial and distal ends). The protocone is more mesially and peripherally located than the paracone (close to the mesiolingual corner of the crown) and there is no lingual cingulum (except for a tiny remnant distolingually from the protocone). The two cusps are connected by a sinuous and fine hypoparacrista that extends from the preparacrista to the mesiobuccal aspect of the protocone. Together with the long, steep, and straight preparacrista, as well as the shorter and mesiobuccally oriented preprotocrista, the hypoparacrista defines a restricted (broader than long) mesial fovea that is centrally located. The distal basin is trapezoidal (wider distally than mesially) and much more extensive and deeper than the mesial fovea, being delimited by the straight and short postparacrista buccally and by the curved and longer postprotocrista lingually. The latter crest becomes continuous with the distal marginal ridge, whereas the postparacrista shapes an abrupt angle with the latter. The occlusal surface shows some enamel wrinkling. A subtle longitudinal groove can be discerned on the deepest portion of the distal basin but it does not interrupt the hypoparacrista.

The P4 occlusal morphology cannot be adequately ascertained in the holotype due to wear (Fig. 2m) and is better preserved in the less worn specimen IPS44014 (Figs. 2n and 4d) as well as in the germ of IPS42977, which is still in crypt but was digitally reconstructed based on microCT scans (Fig. 4e). The P4 crown displays a slightly suboval occlusal contour that is broader than long (BLI = 128–166%; Table 1), and slightly longer buccally than lingually, where the crown displays a more marked convex profile (more tapering in IPS44014 and seemingly IPS42977 than it the holotype). The size of the P4 relative to the P3 cannot be adequately assessed in the holotype due to damage, but in IPS44014 the former is slightly smaller in all dimensions. As in the P3, the P4 protocone is slightly more mesially located than the paracone, and both cusps are buccolingually compressed (albeit the protocone is larger and more bulbous). In contrast, in the P4 the paracone is more peripheral and the protocone is less peripheral than in the P3. No distinct distal cuspules are present except for a rudimentary hypocone close to the distolingual corner of the crown. The mesial crests display a similar orientation as in the P3, but the P4 preparacrista is shorter than that of the P3 (subequal in length to the preprotocrista). The mesial fovea is similarly developed, being much wider than long and crescent-shaped (except in IPS42977), mostly located on the buccal half of the crown. The hypoparacrista similarly extends from the mesiolingual aspect of the paracone (close to the origin of the preparacrista) to the mesiobuccal aspect of the protocone base. Unlike in the P3, there is a

longitudinal groove that extends from the mesial fovea to the distal basin, although it does not interrupt the hypoparacrista. As in the P3, the distal basin is spacious and quadrangular, only slightly longer than broad, much larger and deeper than the mesial fovea, and displays some enamel wrinkling. The postparacrista is longer than in the P3 (and thus more similar in length to the postprotocrista), but similarly forms an abrupt angle with the distal marginal ridge. The buccal cingulum is variably developed, being more marked in IPS42977, where it is nevertheless discontinuous. The lingual cingulum is restricted to the lingual and distolingual aspects of the protocone but much better developed than in the P3 in which it is merely a remnant.

Upper molars Several first and second upper molars are available, including the M1–M2 of the holotype (Figs. 2m and 4a) and IPS43758 (Fig. 2o), the M1 of IPS44014 (Figs. 2n and 4f), the isolated M1 IPS100379 (Figs. 2p and 4g), and the isolated M2s IPS94888 (Figs. 2q and 4i) and IPS100384 (Figs. 2r and 4h). Their occlusal morphology can be better ascertained in IPS44014 and IPS100379 (M1), as well as IPS94888 and IPS100384 (M2). They display a similar subrectangular occlusal contour that is slightly wider than long (BLI = 117–122% in the M1 and BLI = 117–127% in the M2; Table 1), as well as more markedly convex and somewhat tapering lingually (with most specimens displaying a variably developed lingual constriction between the mesial and distal lobes). The M2 is larger and more lingually than the M1. Both molars are low-crowned and display four main pyramidal and buccolingually compressed cusps, the buccal ones being more peripheral and more mesially situated than the corresponding lingual ones. However, the hypocone is more peripheral than the protocone, being located close to the distolingual corner of the crown. The two buccal cusps are similar in size and shape in the M1, the paracone being slightly higher than the metacone, but the metacone is smaller in the M2. The protocone is larger but lower than the buccal cusps, whereas the hypocone is the smallest. There is some variability in the degree of cusp compression, crest sharpness, lingual cingulum development, and hypocone size and shape among the available sample of M1s and M2s. However, unworn and slightly worn specimens (IPS94888, IPS100379, and IPS100384) invariably display a degree of cusp compression and crest sharpness that, due to wear, was not evident from the holotype when it was first described. The preparacrista is very short and slightly obliquely oriented, whereas the preprotocrista is much longer and more inclined. A small paraconule (= protoconule) at the junction between the preprotocrista and the marginal ridge can barely be discerned. The hypoparacrista is somewhat sinuous and slightly oriented distolingually,

extending from the mesiolingual aspect of the paracone base or the preparacrista to the mesiobuccal aspect of preprotocrista. The hypoparacrista distally encloses the small mesial fovea (which is broader than long and mostly located on the buccal half of the crown) from the much larger and deeper trigon basin. The latter is subtriangular in shape and approximately as broad as long, distally delimited by a continuous crista obliqua much longer than the hypoparacrista, and buccally delimited by the similarly long and continuous postparacrista and premetacrista. The distal fovea is wider, shorter, and more centrally located than the trigon basin. The short prehypocrista links the hypocone with the distal aspect of the protocone but there is also a similarly developed crest originating from the hypocone that joins the postprotocrista portion of the crista obliqua, in some specimens flanked by a secondary crest or cuspule-like developments visible at the enamel-dentine junction. The postmetacrista is short and rather mesiodistally aligned, whereas the posthypocrista is similarly short but curved and more continuous with the distal marginal ridge. The buccal cingulum is discontinuous and more developed between the two buccal cusps, whereas the lingual cingulum is much wider and ledge-like (especially in the M2), extending from the protoconule to the prehypocrista (i.e., not distally from the hypocone) and being frequently disrupted by one or more secondary crests that radiate from the base of the protocone. Both M1 and M2 display two buccal roots and a single lingual root with a biconvex lingual contour in cross section.

The M3 is only represented by the two antimeres of the holotype (Figs. 2m and 4b). It differs from the preceding molars in the shorter length (resulting in a relatively much broader proportions: BLI = 139–149%), the lesser development of the talon (with smaller metacone and rudimentary hypocone), and the trapezoidal occlusal contour (with a convex lingual side and an obliquely oriented buccal side). The mesial fovea, although similarly restricted, is slightly broader than in the preceding molars, whereas the trigon basin is more triangular and somewhat smaller due to the more median location of the metacone relative to the paracone. The metacone is comparatively smaller and less distinct, being located on the distobuccal corner of the crown without a distinct postmetacrista. The hypocone is rudimentary (much lesser developed than in the preceding molars). Some development of enamel wrinkling can still be discerned on the distal fovea in spite of wear. The development of the buccal cingulum is similar to that of the M2, being more developed around the paracone (although it is partially interrupted) and progressively narrowing until it reaches the metacone, where it fades away. The lingual cingulum is much broader, as in the preceding molars, constituting a curved shelf-like

extension around the protocone that displays secondary enamel ridges that radiate from the base of this cusp.

Lower incisors The lower incisors are represented by the left i1 and i2 germs of IPS43936 (Fig. 3c–d) and the left i2 IPS44273 (Fig. 2a'). The i1 crown is quite symmetrical (only minimally higher mesially than distally) and displays three mamelons on the unworn incisal edge. It is mesiodistally longest close to the incisal edge, progressively tapering but becoming labiolabially wider toward the cervix. The mesial and distal ridges are not very marked and the lingual surface is concave and devoid of distinct structures, except for two subtle vertical grooves on the apicalmost portion of the crown.

The i2 morphology markedly differs from that of the i1, being characterized by a very asymmetrical crown that progressively becomes labiolingually wider toward the cervix but displays a similar mesiodistal length throughout its apical half. The crown is mesiodistally shorter than labiolingually broad (BLI = 127% in IPS44273) The mesial margin displays a moderately convex profile, whereas the distal margin displays a distinct distal prong that shapes a marked angulation at about crown midheight (the prong has been eroded by wear in IPS44273, but its position can be inferred based on the angulation of the distal margin). While IPS44273 appears moderately high-crowned based on maximum preserved labial height (4.5 mm), the i2 germ IPS43936 indicates that the crown would have originally been somewhat higher (based on the inferred position of the prong). Based on IPS44273, the i2 displays a marked and ledge-like lingual cingulid above a bulbous basal swelling, whereas there is no basal swelling in the i1 germ. The lingual aspect of the i2 crown displays a marked mesial pillar, running from the crown apex to the lingual cingulid, that separates the mesial fovea from the distal one, the latter being more extensive than the former. In IPS44273 only a faint central crest runs vertically through the distal fovea. The root of the i2 is very mesiodistally compressed and constricted toward the cervix, which shapes an inverted V-shape both mesially and distally.

Lower canine The lower canines are represented by the left c1 IPS43433 (Fig. 2b') and the c1 germ of IPS43936 (Fig. 3e). Both may be attributed to female individuals based on crown size and shape, whereas the male c1 remains unknown. The crown is low (preserved labial height = 5 mm in IPS43433) and displays an oval occlusal contour that is longer than broad (BLI 76% in IPS43433), with maximum breadth attained mesially from the crown midlength. The crown is much higher labially

than on the lingual side, where the cervix shapes a shallow inverted V-shape. The occlusal contour is moderately convex lingually and otherwise more markedly convex. However, while the mesial crown wall is uniformly convex, both mesiolingual and distolabial crown walls are concave (in the latter case, being accentuated by wear against the C1), being separated from one another by three steep cristids that originate from the single main cuspid. The mesiolingual cristid is marked but short, ending at about crown midheight in a cuspulid-like enamel developed on the narrow but distinct lingual cingulid, which extends until the distal end of the crown but progressively fades out mesially. The distolingual cristid is fainter and longer, merging with the lingual near the distal end of the crown. The distobuccal cristid is the longest, being somewhat affected by wear. The root is long and labiolingually compressed, somewhat constricted basally below the cervix and progressively tapering along its apical two-thirds.

Lower premolars The p3 germ of IPS43936 is too incompletely formed to adequately ascertain occlusal morphology (Fig. 3f), although some details can be ascertained. There is a single main cuspid (the protoconid) probably located on the mesial half of the crown. Three steep and sharp cristids originate from its apex: the preprotocristid mesiolingually, the postprotocristid distally, and the hypoprotocristid distolingually. The two latter cusps define a steep distal fovea but, given that the crown is not completed, their length and the development of distal cuspulids cannot be ascertained.

The occlusal morphology of the p4 cannot be adequately ascertained because available specimens are quite worn and incompletely preserved (IPS43820 and IPS93524; Figs. 2s–t and 4j) or too incompletely formed (IPS43936; Fig. 3g), although the very partial crown germ of the latter enables a better assessment of the morphology of the mesial cuspids than the remaining specimens. The crown displays an elliptical to suboval contour (longer than broad), with its main mesiodistal axis apparently exorotated (as judged from the position of the distal contact facet against the m1 in IPS93524). Based on IPS93524 and IPS43936 the metaconid seems more buccolingually compressed and peripheral than the protoconid, which is higher and more extensive. The two cusps are connected by a well-defined transverse cristid formed by the hypoprotocristid and hypometacristid, which separates the mesial fovea from the larger and deeper talonid basin, which occupies most of the distal portion of the crown. The two available specimens display a secondary cristid on the mesial fovea, extending from the base of the protoconid (IPS43936) or from the hypoprotocristid-hypometacristid junction (IPS93524) to the mesiolingual end of the crown. The premetacristid is poorly defined in both

specimens. The postmetacristid and the longer postprotocristid are more distinct and distally oriented, terminating at the vestigial entoconid and hypoconid, respectively (only observable in IPS93524). There is no lingual cingulid, while a discontinuous buccal cingulid can be discerned at least distally from the protoconid at about crown midlength. In both IPS43820 and IPS93524 there are two roots (the distal one slightly larger than the mesial) which appear partially fused as one, each one displaying an elliptical cross-section and being somewhat obliquely oriented.

Lower molars The m3 is as yet unknown, whereas the m1 and m2 can be described based on the unerupted germs digitally extracted from IPS43936 (Figs. 3 h–i and 4k–l) as well as on the basis of the m2 germ mesial fragment IPS94886 (Figs. 2u and 4m). The m1 and m2 show very similar occlusal morphology except for the larger dimensions of the latter. Their occlusal contour is subrectangular (much longer than broad; BLI = 76–77%), slightly broader distally, with a somewhat convex mesial contour and very mildly constricted buccal and lingual sides between the mesial and distal lobes. They have five main cuspids of pyramidal shape, the buccal ones being less buccolingually compressed and peripherally located than the lingual ones, whereas the hypoconid and entoconid are more peripheral than the trigonid cuspids. The protoconid is clearly more mesially located than the metaconid, whereas the hypoconid and entoconid are transversely aligned. The hypoconulid is centrally located at the distal end of the crown in the m1 and slightly more buccally and less distally situated in the m2. The trigonid is higher than the talonid and displays an extensive mesial fovea that is only minimally broader than long. This fovea is delimited by the protoconid base and the short preprotocristid buccally, the longer and curved premetacristid (which becomes continuous with the mesial marginal ridge) lingually, and a continuous and slightly oblique cristid distally, formed by the merging of the hypoprotocristid (originated from the distolingual aspect of the protoconid) and the hypometacristid. There is also a secondary transverse cristid that originates from the mesiolingual aspect of the protoconid, terminating at the center of the mesial fovea (IPS43936) or completely dividing it into two distinct foveae (IPS94886). The talonid basin is very extensive and somewhat longer than broad, progressively broadening distalward. It is delimited lingually by a short postmetacristid and a much longer and curved pre-entocristid, while buccally it is closed by a cristid obliqua constituted by a short postprotocristid and a much longer prehypocristid. Both buccal cristids are continuous in the m1, shaping a straight but mesiolingually tilted cristid obliqua that terminates close to the origin of the hypoprotocristid, whereas in the m2 the prehypocristid is more mesiodistally

aligned and angled relative to the postprotocristid, which originates more buccally than the hypoprotocristid. Both the m1 and the m2 display an incompletely developed pliopithecine triangle: the distal arm is as well developed as the prehypocristid and only minimally shorter, being obliquely oriented toward the center of the talonid basin and originating from the prehypocristid (m1) or the mesiobuccal aspect of the hypoconid (m2); in contrast, there is no distinct mesial arm of the triangle, although a very short secondary crest originating from the hypometacristid (m1) or the merging of the hypometacristid and hypoprotocristid (m2) might be homologous to it. There is no cristid connecting the hypoconid with the entoconid. The posthypocristid and prehypoconulid cristid are short and directed to one another, whereas the hypoentocristid and postcristid are somewhat longer (particularly in the m2) but not completely aligned, thus not completely demarcating the relatively distal fovea located on the distolingual corner of the crown from the rest of the talonid basin. The postentocristid (shorter and more indistinct in the m1 than in the m2) curves to become continuous with the distal marginal ridge. The posthypoconulid cristid is very short in the m2 and lacking in the m1. The buccal cingulid (incompletely formed in the m2 germ) is continuous and moderately well developed, becoming shelf-like around the protoconid and the distal aspect of the hypoconid, but narrower and partly interrupted by secondary ridges along the hypoconid base. There is no lingual cingulid except for a small remnant at the junction between the postmetacristid and the pre-entocristid in the m1.

Deciduous upper incisors The right DI1 germ IPS44393 (Fig. 2c’) shows a spatulate, waisted (i.e., constricted toward the cervix), asymmetrical, and low crown—with maximum labial height (2.9 mm) being lower than mesiodistal length (3.3 mm), although it should be taken into account that the germ is not completed. It displays an elliptical occlusal contour that is mesiodistally longer than labiolingually broad (BLI = 76%) and tapers mesially. The labial crown wall is markedly convex. In contrast, the lingual aspect of the crown is markedly concave except at its basalmost portion. The mesial ridge is slightly longer than the distal, and both are continuous with the moderately ledge-like lingual cingulum. Above the latter, the lingual aspect is rather smooth, only showing very subtle subvertical enamel grooves and folds and a very diffuse central swelling that separates the mesial from the somewhat larger distal fovea but does not constitute a distinct pillar. There is no DI2 available.

Deciduous upper premolars The left dP3 IPS43013 (Fig. 2v) is smaller than the permanent cheek teeth and displays a very asymmetrical, trapezoidal contour somewhat broader than long (BLI =

123%). The lingual side is markedly convex, whereas the buccal profile is almost straight but very obliquely oriented, so that the crown is broader mesially than distally. The mesial cusps are transversely aligned, buccolingually compressed, and much better developed than the distal ones. The paracone is higher and more extensive than the protocone, to which it is connected by a distinct and slightly oblique hypoparacrista that separates rectangular (much broader than long) mesial fovea (further bounded by the short preprotocrista and the somewhat longer preparacrista) from the deeper and much more extensive distal basin. A small hypocone is located on the distolingual corner of the crown, being linked to the distal aspect of the protocone by a short prehypocrista. In contrast, there is no distinct metacone, except for a rudimentary and largely worn ridge-like thickening of the enamel at the distobuccal corner on the crown, where the long postparacrista reaches the distal marginal ridge. There are no traces of a crista obliqua, so that the distal basin is very extensive. No distinct posthypocrista can be distinguished from the distal marginal ridge. There are no well-developed cingula except for a small cingular development between the protocone and hypocone, which displays an oblique groove that slightly extends onto the lingual crown wall but does not interrupt the prehypocrista.

The left dP4 IPS42977 (Figs. 2w and 4n) displays a subtriangular occlusal contour that is only minimally broader than long (BLI = 104%) but much longer buccally than lingually. The crown base is much more convex lingually than buccally or distally, whereas the mesial profile is rather sinuous and very obliquely oriented. The trigon cusps are pyramidal and of similar size, although the paracone is more protruding and more buccolingually compressed than the others. The protocone is less peripheral than the buccal cusps, and located in an intermediate position between the latter along the mesiodistal crown axis. The hypocone is much smaller than the trigon cusps and more peripheral than the protocone, being situated close to the distolingual corner of the crown. A short and thick preparacrista extends from the paracone apex to the mesiobuccal corner of the crown. The preprotocrista is straight, sharper and longer than the preparacrista, and obliquely oriented in a mesiobuccal direction, merging with mesial marginal ridge at about crown midline. The sinuous and distinct hypoparacrista originates from the mesiolingual aspect of the paracone and merges with the preprotocrista at about its midlength, thus completely separating the slit-like mesial fovea (mostly located on the buccal half of the crown) from the more extensive and deeper trigon basin. The trigon basin is polygonal in shape, being approximately as long as broad, being buccally enclosed by the

long postparacrista and the shorter premetacrista (which merge with one another forming an obtuse angle) and distally delimited by a fine and slightly curved crista obliqua. The latter separates the trigon basin from the similarly extensive but rectangular (broader than long) distal fovea, which is lingually delimited by the hypocone and its associated crests, including a fine and short prehypocrista (directed toward the distal aspect of the protocone) and an even shorter posthypocrista. In turn, a longer but blunt and indistinct postmetacrista further delimits the distal fovea on the buccal side. The distal marginal ridge is not very well defined. No enamel wrinkling can be discerned on the occlusal basins. The buccal cingulum is narrow and rather discontinuous (partly interrupted at the level the buccal cusps by secondary folds of the enamel), whereas the C-shaped lingual cingulum is much wider (shelf-like), extending around the protocone until the mesial aspect of the hypocone and the prehypocrista, being interrupted by a secondary enamel fold that radiates from the distolingual aspect of the protocone base. Based on the microCT scan data, there is a single lingual root of subquadrangular cross-section as well as two distinct and divergent buccal roots that are more triangular and buccodistally compressed.

Deciduous lower incisors No deciduous lower incisors are available, only their alveoli are preserved in IPS43936 (Figs. 1g–l and 3a). Those of the di1 (right: MD = 2.6 mm, BL = 2.7 mm; left: MD = 2.5 mm, BL = 2.7 mm) indicate that the root base was only slightly mesiodistally compressed, whereas the left di2 alveolus (MD = 1.7, BL = 2.4 mm) indicates that the lateral incisor was somewhat smaller than the central and displayed a more mesiodistally compressed root basally.

Deciduous lower canine As for the deciduous lower incisors, only the left dc1 alveolus is preserved in IPS43936 (Fig. 1g–l and 3a; MD = 3.2, BL = 2.5 mm), suggesting that this tooth was somewhat buccolingually compressed and that, despite being aligned with the postcanine deciduous dentition, its main mesiodistal axis was somewhat exorotated relative to the postcanine tooth row.

Deciduous lower premolars The crown of the single available dp3 IPS43936 (Figs. 2x and 4o) is distally broken but apparently displayed a suboval occlusal profile longer than broad and widest distally, with a markedly convex and tapering mesial outline. Only the two trigonid main cuspids are preserved. The protoconid is very buccolingually compressed and centrally located; a marked, straight and long preprotocristid descends from the protoconid apex until the mesial end of the crown. The metaconid is lower, smaller, more bulbous, and as peripheral as but more distal than the protoconid. No distinct postprotocristid originates from the protoconid. Instead, a short hypoprotocristid originates

distally from the protoconid and curves until merging with the more transversely aligned hypometacristid, separating the longer than broad mesial fovea from the deeper and more extensive trigonid basin. This mesial fovea, located on the lingual half of the crown, is steeply inclined and lingually open, as there is no distinct premetacristid. No paraconid is present either at the mesial end of the crown. The preserved portion of the talonid basin indicates that it was originally longer than broad, and preferentially located on the lingual moiety of the crown, being buccally delimited by a very obliquely oriented cristid obliqua, apparently constituted exclusively by the prehypocristid, as it merges with the junction between the hypoprotocristid and the hypometacristid. The preserved portion of the seemingly postmetacristid progressively curves toward the distolingual portion of the crown, suggesting that the entoconid (like the hypoconid, given the inclination of the cristid obliqua) would have originally been much more peripheral than the trigonid cuspids. Cingulids are restricted to the mesiobuccal and especially the mesiolingual aspect of the crown, flanking the terminal portion of the preprotocristid. MicroCT scan data show that this tooth is biradiculate.

The two available dp4s (IPS43936 and IPS106878; Figs. 2x–y and 4p–q) display a subquadrangular to suboval occlusal contour that is much longer than broad (BLI 72.3–76%; Table 1) and, like in the dp3, wider distally, with mesially tapering convex outline. There are five main cuspids as well as a poorly-developed paraconid (unlike in the dp3). The protoconid and hypoconid are more buccolingually compressed, more mesially located, more protruding, and less peripheral than their lingual counterparts, with the entoconid being the most peripheral cusp. In IPS106878, the buccal cuspids (especially the hypoconid) are slightly more median than in IPS43936. The hypoconulid has a bulbous shape and is the smallest cusp, being centrally located close to the distal marginal ridge. A thick and short preprotocristid (shorter than in the dp3) and a shaper and slightly longer premetacristid (indistinct in the dp3) extend in mesial to mesiobuccal direction from the protoconid and metaconid apices, respectively, to the mesial marginal ridge. A poorly-developed paraconid is present at the junction of premetacristid, the mesial marginal ridge, and a secondary oblique cristid (absent from the dp3) that originates from the protoconid and splits the mesial fovea. No postprotocristid distinct from the hypoprotocristid originates from the distal aspect of the protoconid; in contrast, a single short cristid (that we interpret as the hypoprotocristid) originates distolingually from the protoconid and curves until merging with the more transversely aligned hypometacristid. They conform a continuous cristid that separates the somewhat longer than broad mesial fovea from the deeper and more

extensive talonid basin. The talonid basin is longer than broad, and more lingually located than the mesial fovea (especially in IPS106878). Lingually, the talonid basin is delimited by a slightly curved postmetacristid that is directed toward the straighter pre-entocristid. Buccally, the talonid basin is delimited by the very obliquely oriented cristid obliqua (more so in IPS43936 than in IPS106878) constituted by the prehypocristid and, according to our interpretation, the displaced postprotocristid, which reaches the junction between the hypoprotocristid and the hypometacristid. The hypoconid and hypoconulid are connected by the short but well-developed posthypocristid and the prehypoconulid cristid. In contrast, the postentocristid can barely be discerned, curving distobuccally to become continuous with the distal marginal ridge, whereas a posthypoconulid cristid is lacking. There is no hypoentocristid or postcristid, so that the distal fovea, located on the distolingual corner of the crown, is completely open to the talonid basin. As in m1–m2, in the dp4 there is no lingual cingulid except for a small remnant at the junction between the postmetacristid and the pre-entocristid (less marked in IPS106878). The buccal cingulid is moderately developed (slightly more so in IPS43936 than IPS106878) but discontinuous, being widest between the protoconid and the hypoconid, but interrupted at the buccal level of these cusps. In IPS43936 there are two (mesial and distal) roots of bilobed cross-section, each one corresponding to a pair of fused roots.

Supplementary Text 4. Results of the additional cladistic analysis

The dental-only analysis at the genus level based on a wider representation of taxa (Supplementary Fig.6a) also recovers pliopithecoids as a clade of stem catarrhines but displays some differences relative to the dental-only analysis at the species level reported in the main text (Fig. 6). In particular, dendropithecids are recovered as a paraphyletic assemblage of stem cercopithecoids instead of a stem-catarrhine clade more derived than pliopithecoids, which we consider an unreliable result based on current knowledge of catarrhine evolution and the results of multiple previous cladograms (see main text). In contrast, *Ekembo* is recovered as a stem hominoid and both *Pierolapithecus* and *Hispanopithecus* as crown hominoids, in agreement with previous results based on different matrices[1,2], although the molecular phylogeny of crown hominoids is not recovered. The most parsimonious topology of the dental-only analysis at the genus level further displays some differences from that reported in the main text regarding the internal topology of pliopithecoids, with *Anapithecus* being more derived than both *Barberapithecus* and *Pliobates* toward *Plesiopliopithecus* + *Crouzelia*. Nevertheless, both analyses recover the monophyly of crouzeliids and support the placing of *Pliobates* within this clade. The differences in the most parsimonious arrangement of pliopithecoids might simply result from the more restricted representation of pliopithecoid taxa in the genus-level analysis than in that reported in the main text, the latter being arguably more reliable because of this reason. The craniodental analysis (Supplementary Fig. 6b) yields very similar results (identical regarding *Pliobates* and other pliopithecoids), but recovers the monophyly of crown hominids (in agreement with molecular data), with *Hispanopithecus* being supported as a stem hominid and *Pierolapithecus* as a pongine.

Unlike the dental and craniodental analyses, the postcranial-only analysis is not well-resolved (Supplementary Fig. 6c), because most clades (including pliopithecoids, dendropithecids, and cercopithecoids) are collapsed except crown hominoids, hominids, and a hominoid clade that excludes *Ekembo* but includes *Pliobates* as the only stem hominoid. This cladogram indicates that postcranial characters are informative regarding the internal phylogeny of crown hominoids (recovering the molecular topology) but yield very contradictory results for *Pliobates* as compared with the (cranio)dental dataset. When the postcranial characters are analyzed simultaneously with the craniodental ones, the former play an important role in determining the topology of *Pliobates* (and, indirectly, of other pliopithecoids). Thus, a well-resolved consensus tree is obtained (Supplementary

Fig. 6d), although displaying a different topology than that based on dental or craniodental characters (Supplementary Fig. 6a–b). In particular, the phylogenetic position of *Ekembo* is not resolved, and while the molecular phylogeny of crown hominoids is recovered and dryopithecines are supported as stem hominids (in agreement with refs. 1 and 2), the results for dendropithecids and pliopithecoids are very heterodox and reminiscent of those previously obtained by Alba et al.[1]. In particular, dendropithecids and pliopithecoids are supported as successive paraphyletic assemblages of stem hominoids, which is probably an artifact caused by several factors: first, the omission from the analysis of a wider representation of Early and Middle Miocene apes from Africa (such as afropithecids and nyanzapithecids) that are customarily considered stem hominoids; second, the large amount of missing data in most dendropithecids and pliopithecoids; and, third, the possession of multiple crown hominoid-like postcranial features in *Pliobates*. The fact that *Pliobates* shares both dental derived features with pliopithecoids and postcranial derived features with hominoids appears to be pulling the whole pliopithecoid clade toward crown hominoids. This is similar to what has previously been argued for *Oreopithecus*—a likely stem hominoid that clusters with crown hominoids in total evidence analyses presumably owing to postcranial convergences[2]—as well as hypothesized for hylobatids and hominids among crown hominoids[3].

Given the contradictory results obtained for (cranio)dental data vs. total evidence (i.e., further including the postcranial data), two alternative interpretations are possible. It might be argued on cladistic epistemological grounds that the results based on all the available evidence are always preferable. Such reasoning seems logical on theoretical grounds but in this particular case would imply supporting not only *Pliobates*, but all other pliopithecoids, as members of the hominoid stem lineage—which goes against other, independently derived cladograms as well as generally accepted knowledge about ape evolution. Alternatively, it may be argued that the total evidence results are biased by postcranial parallelisms between *Pliobates* and hominoids and, thus, less realiable than the most parsimonious topology based on (cranio)dental data. While the phylogenetic position of pliopithecoids from Eurasia relative to dendropithecids and putative stem hominoids from Africa certainly deserves further research, several lines of evidence favor the second interpretation that postcranial features are biasing the results and that pliopithecoids (including *Pliobates*) constitute a catarrhine clade that is not closely related to crown hominoids. This is directly supported by the fact that our postcranial-only analysis does not resolve the phylogenetic relationships of most Miocene

catarrhines (with the exception of dryopithecines), so that postcranial evidence is introducing more ‘noise’ than true phylogenetic signal regarding these taxa (especially *Pliobates*). Also, more indirectly, the contention that postcranial characters are biasing the cladistic results for particular taxa is further supported by the fact that similar contradictory results between craniodental and postcranial data have been previously found among hominoids[2], being attributed to postcranial homoplasy between *Oreopithecus* and crown hominoids, as well as between hylobatids and hominids (for further discussion, see ref. 3). Incidentally, even the total evidence analysis recovers *Pliobates* as closely related to pliopithecoid crouzeliids (branching off, like *Plesiopliopithecus* + *Crouzelia*, between *Barberapithecus* and *Anapithecus*), thereby supporting our taxonomic assessment that the former taxon is a derived crouzeliid.

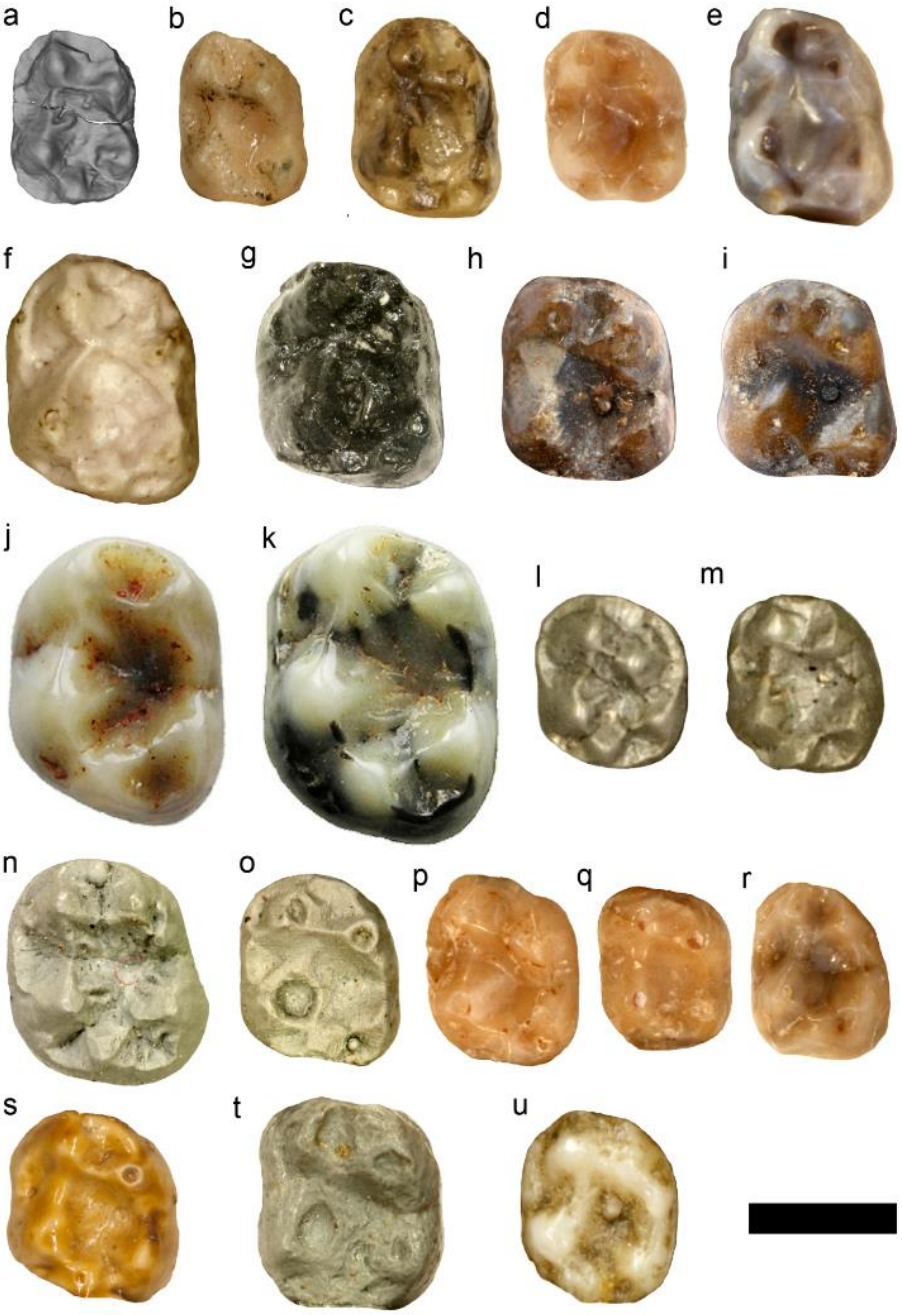

Supplementary Figure 1. Comparisons of the lower first molar occlusal morphology of *Pliobates* and other pliopithecoids. Specimens are depicted in occlusal view as if from the left side (indicated when reversed; mesial is on top). **a** *Pliobates cataloniae*, virtual model rendering of IPS43936; **b** *Crouzelia auscitanensis*, Sa 999 (holotype; cast); **c** *Plesiopliopithecus lockeri*, OLL 2010/6 (holotype; cast); **d** *Barberapithecus huerzeleri*, IPS1724m (holotype); **e** *Egarapithecus narcisoi*, IPS45757 (holotype); **f** *Anapithecus hernyaki*, RUD 9 (holotype; cast); **g** *Anapithecus hernyaki* RUD 98 (reversed; cast); **h** *Laccopithecus robustus*, PA 879 (paratype; reversed; cast); **i** *Laccopithecus robustus*, PA 881 (paratype; cast); **j** *Fanchangia jini*, V 18115.1 (paratype; reproduced

from ref. 4); **k** *Fanchangia jini*, V 18115.17 (paratype; reproduced from ref. 4); **l** *Dionysopithecus shuangouensis*, PA 1249 (cast); **m** *Dionysopithecus shuangouensis*, PA 1251 (reversed; cast); **n** *Platodontopithecus jianghuaiensis*, PA 1225 (reversed; cast); **o** *Pliopithecus antiquus*, MNHN Sa 995 (holotype; cast); **p** *Pliopithecus canmatensis* IPS35036 (holotype); **q** *Pliopithecus canmatensis*, IPS41719 (paratype). **r** *Pliopithecus canmatensis*, IPS41955 (paratype); **s** *Pliopithecus platyodon*, Graz Joanneum 2100 (holotype; cast); **t** *Pliopithecus zhanxiangi*, BGN mandible (paratype; reversed; cast); **u** *Epipliopithecus vindobonensis*, Individual II (cast). Scale = 5 mm.

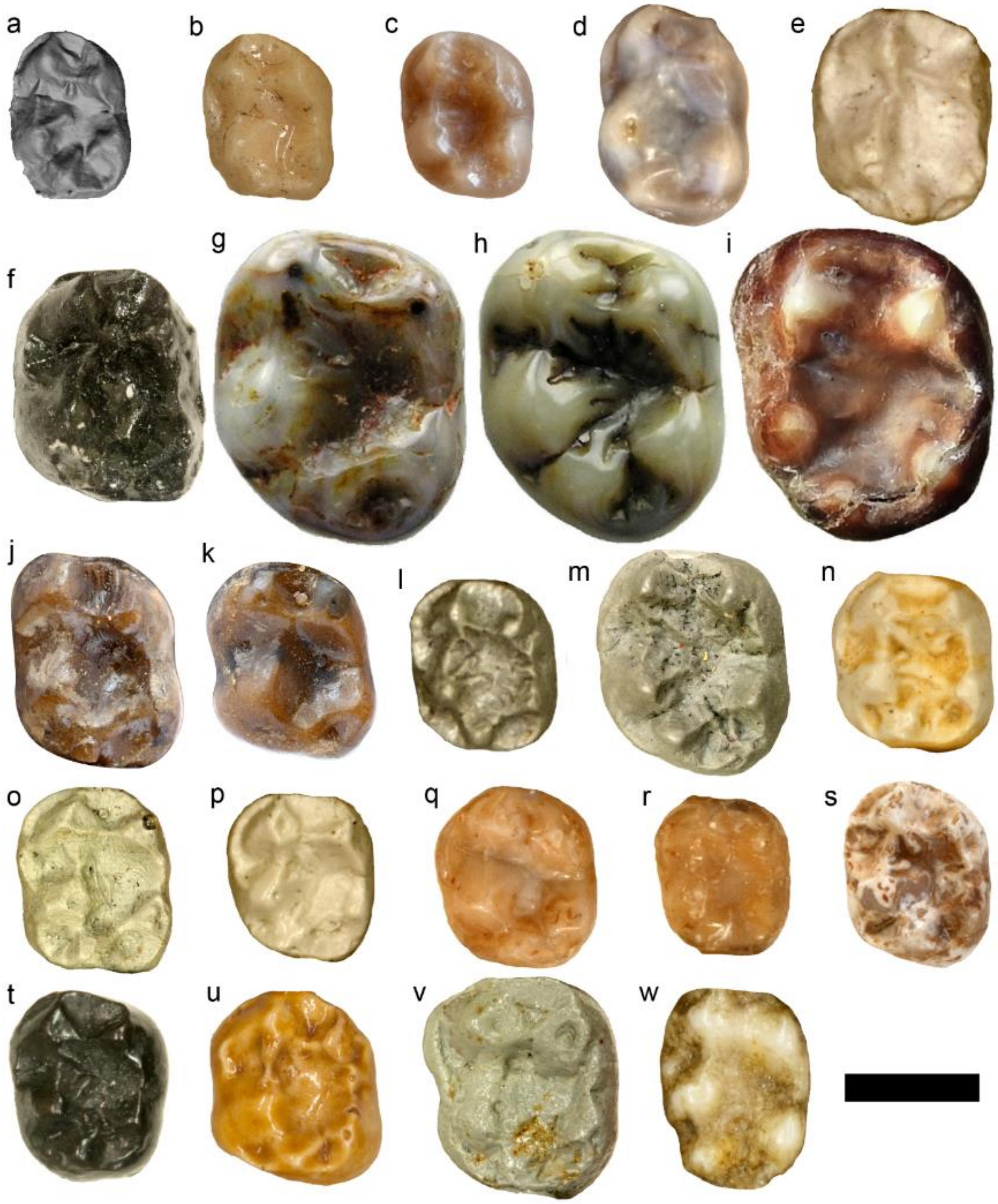

Supplementary Figure 2. Comparisons of the lower second molar occlusal morphology of *Pliobates* and other pliopithecoids. Specimens are depicted in occlusal view as if from the left side (indicated when reversed; mesial is on top). **a** *Pliobates cataloniae*, virtual model rendering of IPS43936; **b** *Crouzelia auscitanensis*, MNHN Sa 999 (holotype; cast); **c** *Barberapithecus huerzeleri*, IPS1724n (holotype; reversed); **d** *Egarapithecus narcisoi* IPS 2943 (holotype); **e** *Anapithecus hernyaki*, RUD 9 (holotype; cast); **f** *Anapithecus hernyaki*, RUD 98 (reversed; cast); **g** *Fanchangia jini* V 18115.2 (paratype; reproduced from ref. 4); **h** *Fanchangia jini* V 18114 (holotype; reversed; reproduced from ref. 4); **i** *Krishnapithecus krishnaii*, PRS04/12 (reproduced from ref. 5); **j**

Laccopithecus robustus PA 879 (paratype; reversed; cast); **k** *Laccopithecus robustus* PA 881 (paratype; cast); **l** *Dionysopithecus shuangouensis*, PA 1252 (reversed; cast); **m** *Platodontopithecus jianghuaiensis*, PA 1220 (cast); **n** *Pliopithecus antiquus*, L. G. 109 (reversed; cast); **o** *Pliopithecus antiquus*, MNHN Sa 995 (holotype; cast); **p** *Pliopithecus antiquus* (reversed; cast); **q** *Pliopithecus canmatensis*, IPS35036 (holotype); **r** *Pliopithecus canmatensis*, IPS41719 (paratype). **s** *Pliopithecus canmatensis*, IPS41660 (paratype; reversed); **t** *Pliopithecus piveteaui*, Collections Lecointre (holotype; cast); **u** *Pliopithecus platyodon*, Graz Joanneum 2100 (holotype; cast); **v** *Pliopithecus zhanxiangi*, BGN mandible (paratype; reversed; cast); **w** *Epipliopithecus vindobonensis*, Individual II (cast). Scale = 5 mm.

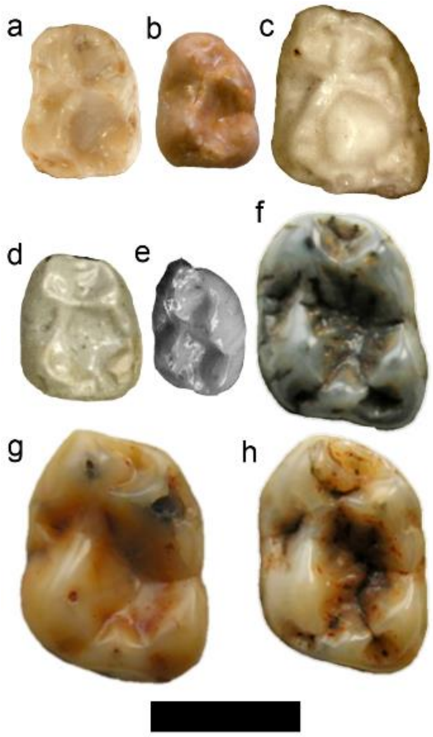

Supplementary Figure 3. Comparisons of the deciduous lower fourth premolar occlusal morphology of *Pliobates* and other pliopithecoids. Specimens are depicted in occlusal view as if from the left side (indicated when reversed; mesial is on top). **a** *Pliobates cataloniae*, IPS43936; **b** *Pliobates cataloniae*, IPS106878; **c** *Anapithecus hernyaki*, RUD 9 (holotype; cast); **d** *Dionysopithecus shuangouensis*, PA 1241 (reversed; cast); **e** *Pliopithecus piveteaui* (reproduced from ref. 6); **f** *Fanchangia jini*, V 18115.15 (paratype; reversed; reproduced from ref. 4); **g** *Fanchangia jini*, V 18115.20 (paratype; reversed; reproduced from ref. 4); **h** *Fanchangia jini*, V 18115.21 (paratype; reversed; reproduced from ref. 4). Scale = 5 mm.

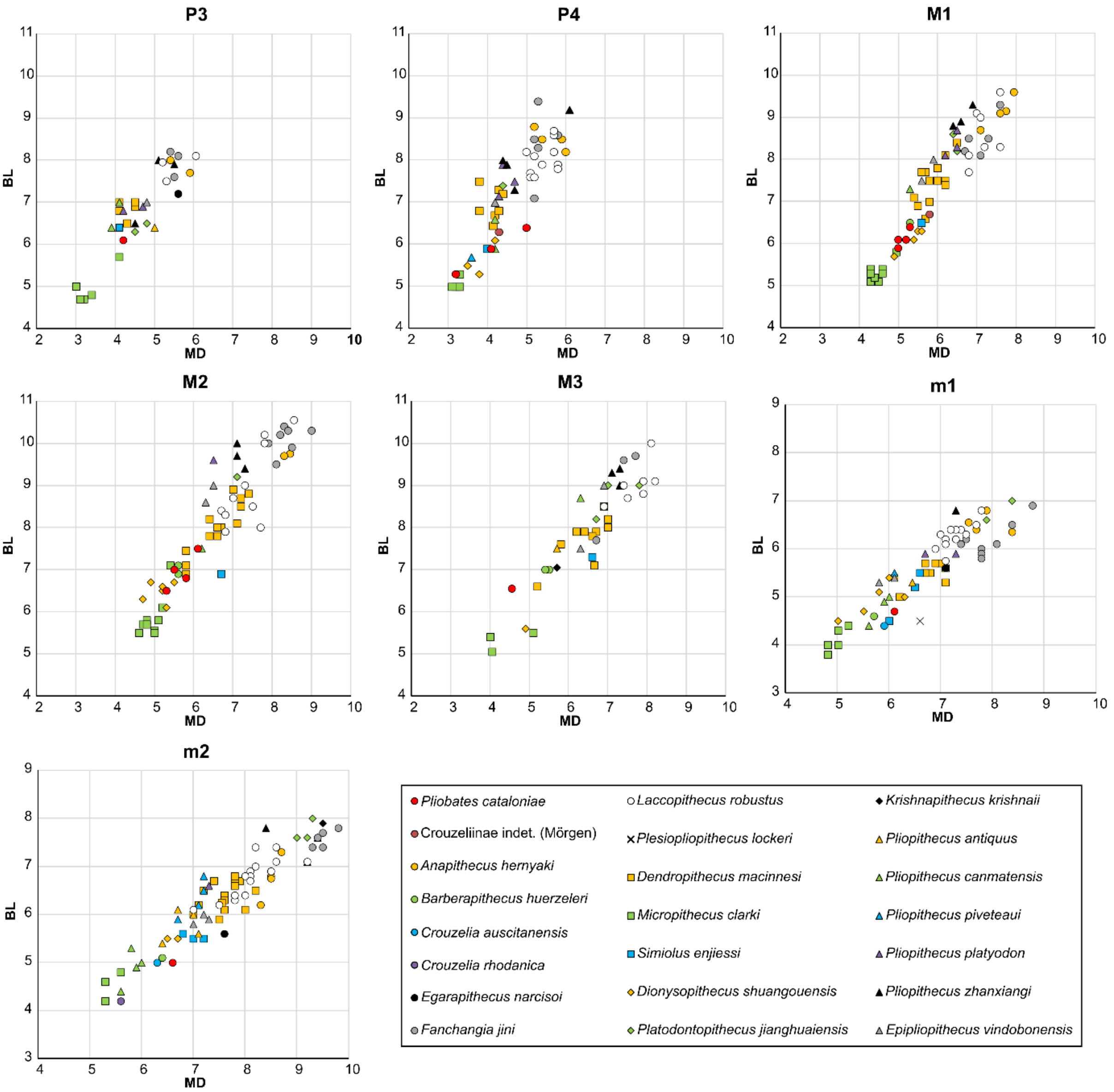

Supplementary Figure 4. Dental size and proportions. Bivariate plots of buccolingual breadth (BL; in mm) vs. mesiodistal length (MD; in mm) in upper and lower cheek teeth of *Pliobates cataloniae* compared with pliopithecoids and dendropithecids. Each species is defined by a unique color-coded symbol. See Supplementary Table 2 for data sources. Source data are provided as a Source Data file.

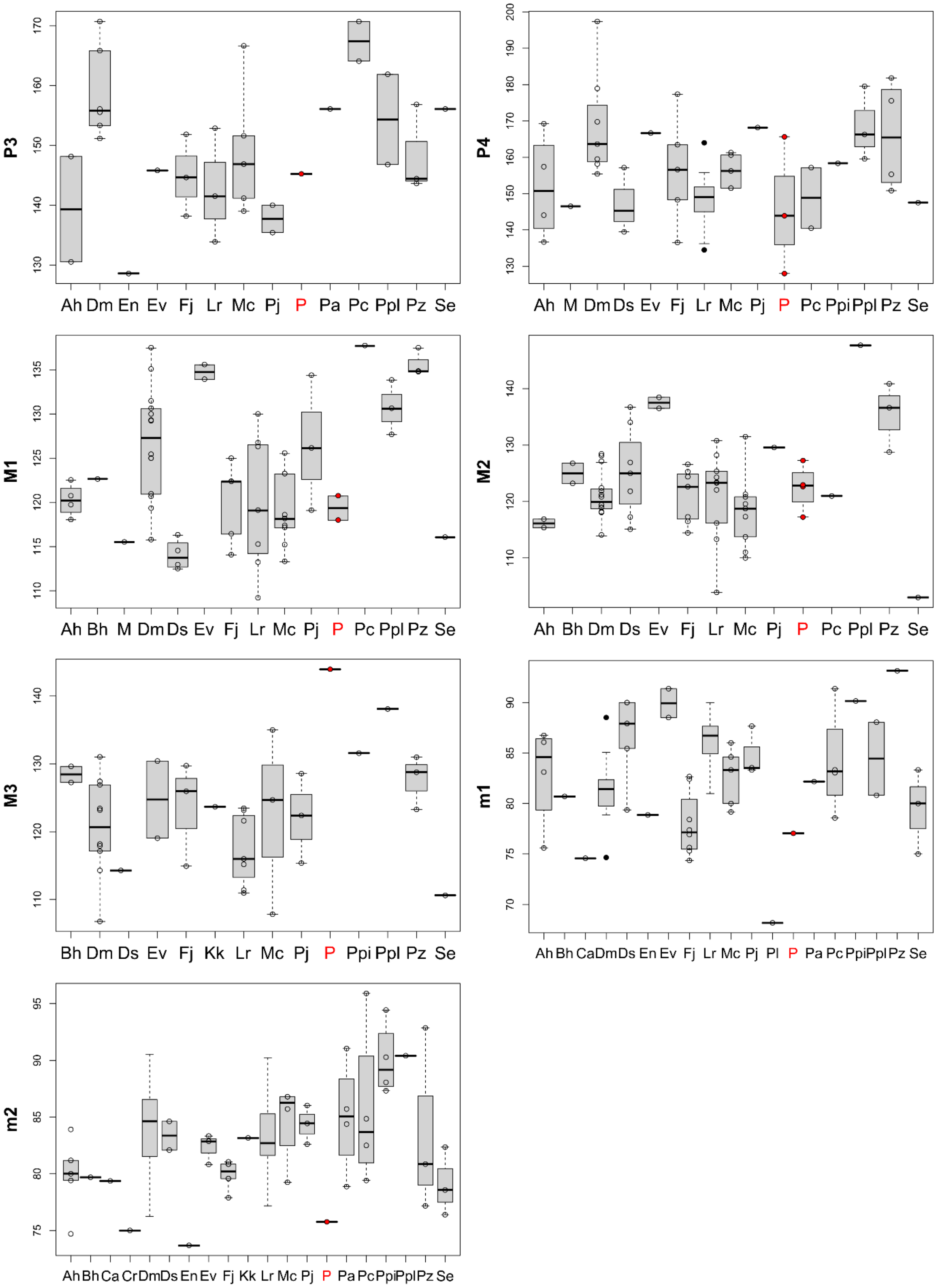

Supplementary Figure 5. Dental proportions. Box-and-whisker plots of breadth/length index (BLI; in %) in upper and lower cheek teeth of *Pliobates cataloniae* compared with pliopithecoids and dendropithecids. White dots (red dots in the case of *P. cataloniae*) represent single specimen values

and are shown when $n \leq 10$. For box-and-whisker plots, horizontal lines denote medians, boxes depict the interquartile range, whiskers the minimum-maximum values excluding outliers, and black dots denote outliers (beyond 1.5 times above or below the interquartile range). Abbreviations: Ah = *Anapithecus hernyaki*; Bh = *Barberapithecus huerzeleri*; Ca = *Crouzelia auscitanensis*; Cr = *Crouzelia rhodanica*; Dm = *Dendropithecus macinnesi*; Ds = *Dionysopithecus shuangouensis*; En = *Egarapithecus narcisoi*; Ev = *Epipliopithecus vindobonensis*; Fj = *Fanchangia jini*; Kk = *Krishnapithecus krishnaii*; Lr = *Laccopithecus robustus*; M = Crouzeliinae indet. from Mörgen; Mc = *Micropithecus clarki*; Pj = *Platodontopithecus jianghuaiensis*; Pl = *Plesiopliopithecus lockeri*; P = *Pliobates cataloniae*; Pa = *Pliopithecus antiquus*; Pc = *Pliopithecus canmatensis*; Ppi = *Pliopithecus piveteaui*; Ppl = *Pliopithecus platyodon*; Pz = *Pliopithecus zhanxiangi*; Se = *Simiolus enjiessi*. See Supplementary Table 2 for data sources. Source data are provided as a Source Data file.

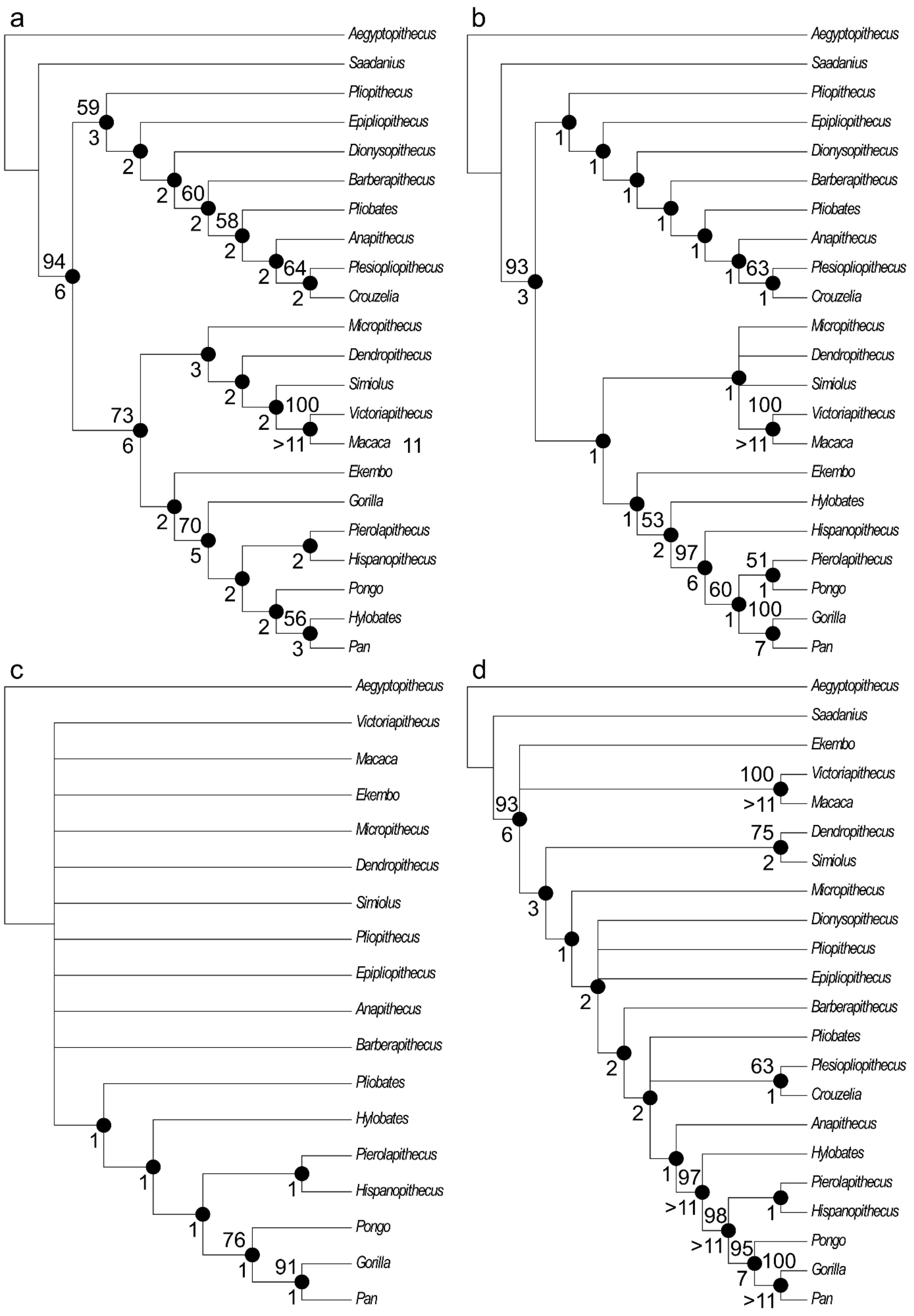
a
Aegyptopithecus
Saadanius
Pliopithecus
Epipliopithecus
Dionysopithecus
Barberapithecus
Pliobates
Anapithecus
Plesiopliopithecus
Crouzelia
Micropithecus
Dendropithecus
Simiolus
Victoriapithecus
Macaca
11
Ekembo
Gorilla
Pierolapithecus
Hispanopithecus
Pongo
Hylobates
Pan
59
3
2
2
60
2
58
2
64
2
2
94
6
3
2
100
2
>11
73
6
2
70
5
2
2
2
56
2
3
b
Aegyptopithecus
Saadanius
Pliopithecus
Epipliopithecus
Dionysopithecus
Barberapithecus
Pliobates
Anapithecus
Plesiopliopithecus
Crouzelia
Micropithecus
Dendropithecus
Simiolus
Victoriapithecus
Macaca
Ekembo
Hylobates
Hispanopithecus
Pierolapithecus
Pongo
Gorilla
Pan
1
1
1
1
1
63
1
1
93
3
1
100
>11
1
1
53
2
97
6
51
1
60
100
1
7
c
Aegyptopithecus
Victoriapithecus
Macaca
Ekembo
Micropithecus
Dendropithecus
Simiolus
Pliopithecus
Epipliopithecus
Anapithecus
Barberapithecus
Pliobates
Hylobates
Pierolapithecus
Hispanopithecus
Pongo
Gorilla
Pan
1
1
1
1
76
1
91
1
d
Aegyptopithecus
Saadanius
Ekembo
Victoriapithecus
Macaca
Dendropithecus
Simiolus
Micropithecus
Dionysopithecus
Pliopithecus
Epipliopithecus
Barberapithecus
Pliobates
Plesiopliopithecus
Crouzelia
Anapithecus
Hylobates
Pierolapithecus
Hispanopithecus
Pongo
Gorilla
Pan
100
>11
93
6
75
2
3
1
2
2
63
1
2
1
97
>11
98
1
>11
95
100
7
>11

Supplementary Figure 6. Results of the additional cladistic analyses considering a wider sample of catarrhines at the genus level. a cladogram derived from the single most parsimonious tree based on 97 dental characters for 22 taxa (tree length = 324 steps; CI = 0.451; RI = 0.613; RCI = 0.276); **b** strict consensus cladogram derived from the two most parsimonious trees based on 175 craniodental characters for 22 taxa (tree length = 543 steps; CI = 0.417; RI = 0.557; RCI = 0.232); **c** strict consensus cladogram derived from the 90 most parsimonious trees based on 206 postcranial characters for 18 taxa (tree length = 353 steps; CI = 0.333; RI = 0.254; RCI = 0.085); **d** strict consensus cladogram derived from the five most parsimonious trees based on 381 postcranial characters for 22 taxa (tree length = 920 steps; CI = 0.400; RI = 0.524; RCI = 0. 210). Bremer's indices and bootstrap percentages (only shown when ≥50%) are reported below and above nodes, respectively. The list of characters and taxon-character matrix can be found in Supplementary Data 4 and 5, respectively.

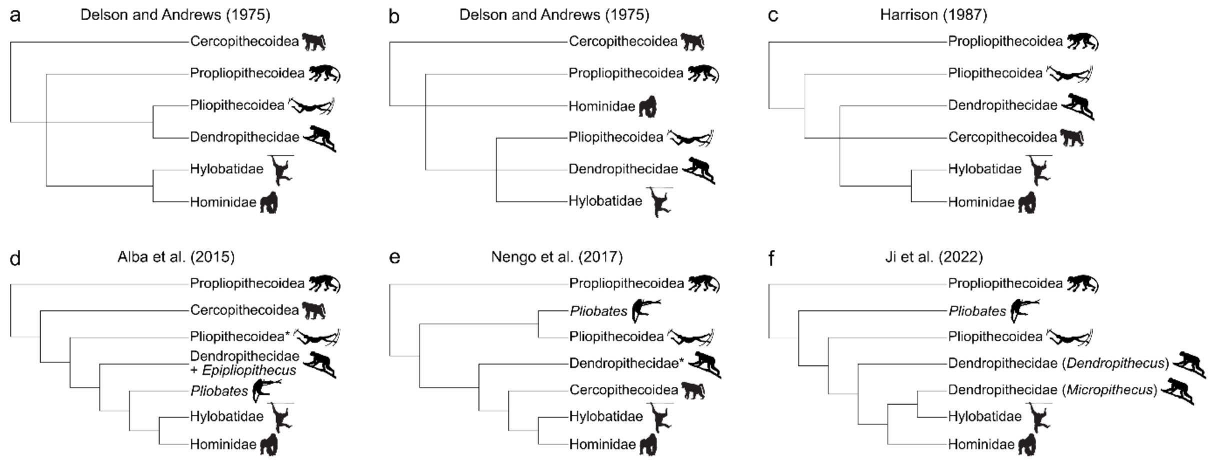

Supplementary Figure 7. Different views on the phylogenetic relationships between major extant and extinct catarrhine groups before and after 1980, including recent cladistic analyses that considered *Pliobates*. a–b alternative hypotheses of Delson and Andrews, 1975 (modified from Fig. 3 in ref. 7); **c** hypothesis of Harrison, 1987 (modified from Fig. 6 in ref. 8); **d-e** results of the cladistic analyses of Alba et al., 2015 (**d**; modified from Fig. 8 in ref. 1), Nengo et al., 2017 (**e**; modified from Fig. 5 in ref. 9), and Ji et al., 2022 (**f**; modified from Fig. 10 in ref. 10). Groups denoted with an asterisk were recovered as paraphyletic, with *Epipliopithecus* being recovered as a dendropithecid instead of a pliopithecoid in ref. 1 and *Simiolus* and *Micropithecus* being recovered as a clade more derived than an East African small-bodied catarrhine clade including *Dendropithecus* in ref. 9. Black silhouettes (not to scale) of Propliopithecoidea, Cercopithecoidea, Hylobatidae, and Hominidae were taken from the following sources: Propliopithecoidea from Fig. 3 in ref. 11; Cercopithecoidea, Hylobatidae, and Hominidae from Summary Figure in ref. 12. Reprinted with permission from AAAS. Black silhouettes (not to scale) of Dendropithecidae and Pliopithecoidea were redrawn from Fig. 15.8 and Fig. 15.12, respectively, in ref. 13. Reprinted with permission from Elsevier.

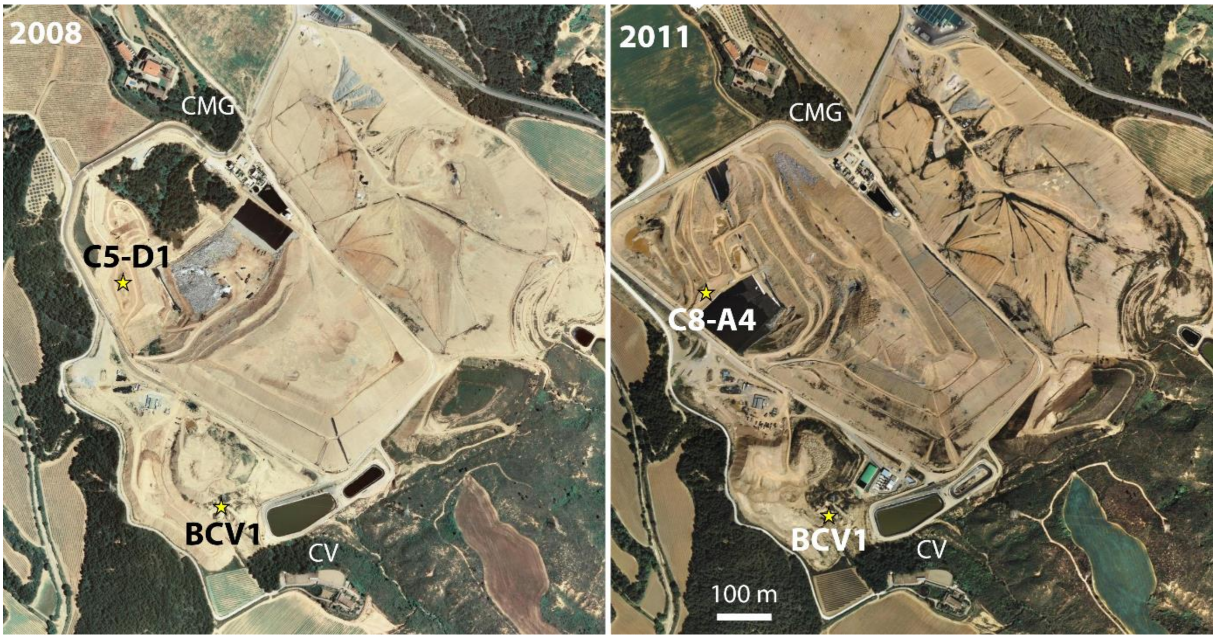

Supplementary Figure 8. Ortophotomaps of Abocador de Can Mata in 2008 and 2011, showing the location of the two localities that have yielded *Pliobates* remains (ACM/C5-D1 and ACM/C8-A4). The location of ACM/BCV1 (the type locality of *Pierolapithecus catalaunicus*) is provided for comparison. Fossil localities are denoted by yellow stars. The situation of the farmhouses of Can Mata de la Garriga (CMG) and Can Vila (CV) are also provided for reference. Base orthophotomaps: © Institut Cartogràfic i Geològic de Catalunya, downloaded from VISSIR v. 3.26 and reproduced with permission by means of a Creative Commons license CC BY 4.0 (ICGC, 2021).

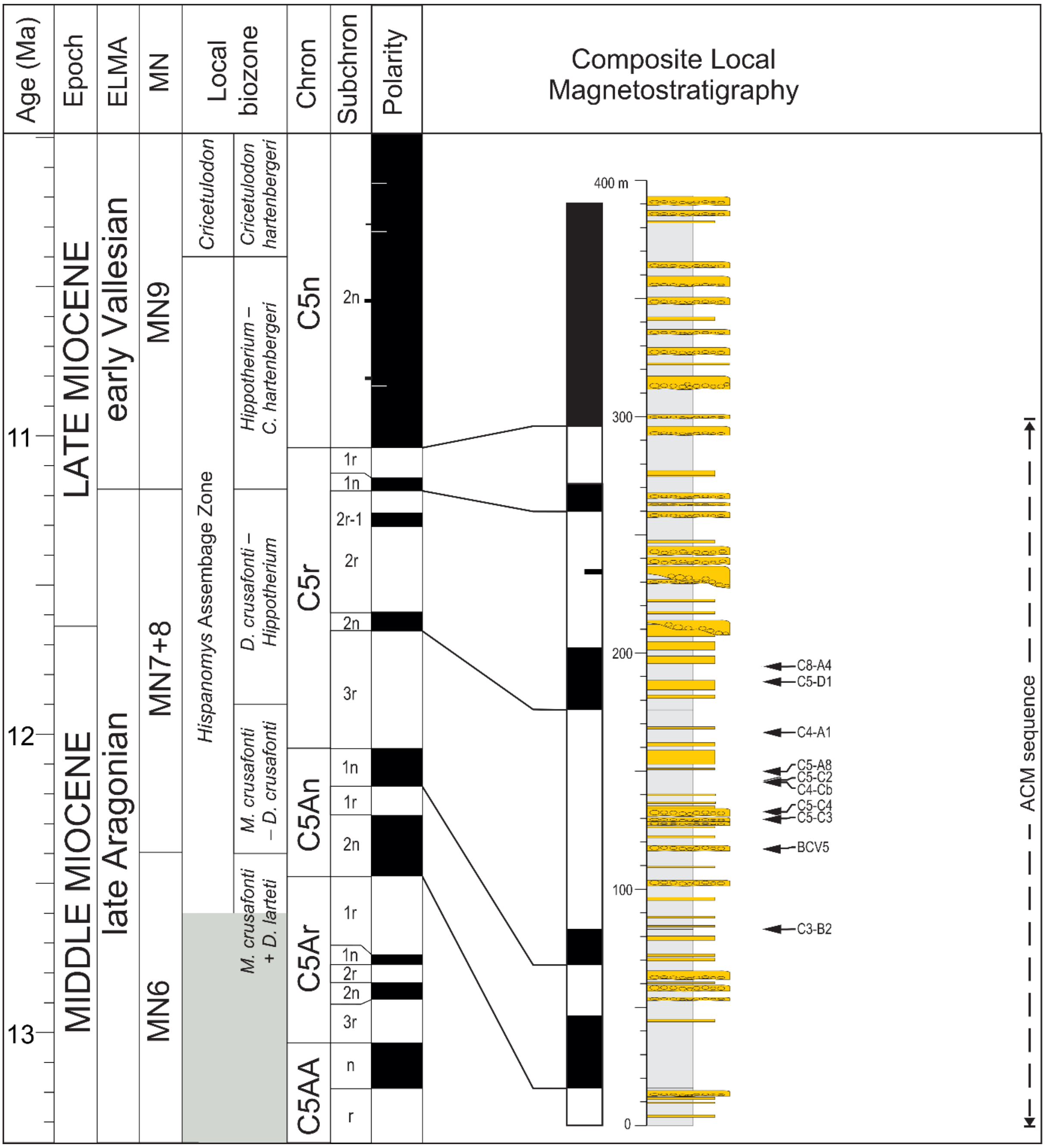

Supplementary Figure 9. Correlation of the composite local magnetostratigraphy of ACM and nearby areas with the Geomagnetic Polarity Time Scale (GPTS). European Land Mammal Ages (ELMA), Mammal Neogene units, and local biozones of the Vallès-Penedès Basin (after 14) are shown on the left. For the local biozonation, a plus symbol denotes a concurrent range subzone, whereas an en dash denotes an interval subzone. The stratigraphic position of the pliopithecoid-bearing localities is shown to the right on the composite lithostratigraphic column (black arrows). *Pliobates cataloniae* is recorded from ACM/C8-A4 and ACM/C5-D1, the Pliopithecoidea indet. (oldest

pliopithecoid occurrence in the Iberian Peninsula) from ACM/C3-B2[15], and *Pliopithecus canmatensis* from all remaining localities. Modified from Fig. 8 in ref. 16.

Supplementary Table 1. Dentognathic remains of *Pliobates cataloniae* from ACM/C5-D1.

Catalogue No.	Anatomical identification	Figure(s)
IPS42977	Left maxillary fragment with DP4	Fig. 2j–l
IPS43013	Left DP3 crown	Fig. 2v
IPS43433	Left c1 crown and nearly complete root; female	Fig. 2b’
IPS43488	Left I1 partial crown and nearly complete root	Fig. 2z
IPS43758	Right maxillary fragment with M1 and damaged M2	Fig. 2d–e
IPS43820	Right p4 broken crown and partial roots	Fig. 2t
IPS43936	Partial infant mandible with left dp3–dp4 and permanent tooth germs inside their crypts; female	Figs. 2g–I and 3
IPS44014	Left maxillary fragment with I1–C1 alveoli and P3–M1; male	Fig. 2a–c
IPS44273	Left i2 crown and nearly complete root	Fig. 2a’
IPS44393	Right DI1 germ	Fig. 2c’
IPS93524	Right p4 partial crown and basalmost portion of the roots	Figs. 2s and 4j
IPS94886	Right m2 germ mesial fragment	Figs. 2u and 4m
IPS94888	Left M2 germ with basalmost portion of the roots	Figs. 2q and 4i
IPS100379	Right M1 damaged crown and partial roots	Figs. 2p and 4g
IPS100384	Left M2 germ	Figs. 2r and 4h
IPS106878	Left dp4 crown	Figs. 2y and 4q

Supplementary Table 2. Comparative sample used in this study.

Taxon	Sources for dental measurements
Dionysopithecus shuangouensis	(17)
Platodontopithecus jianghuaiensis	(17)
Pliopithecus antiquus	(18, 19); authors' own data from casts
Pliopithecus bii	(20)
Pliopithecus canmatensis	(21)
Pliopithecus piveteaui	(19, 22)
Pliopithecus platyodon	authors' own data from casts
Pliopithecus zhanxiangi	(23)
Epipliopithecus vindobonensis	(19); authors' own data from casts
Krishnapithecus krishnaii	(5, 24)
Anapithecus hernyaki	authors' own data from casts
Barberapithecus huerzeleri	(25)
Crouzelia auscitanensis	(26); authors' own data from casts
Crouzelia rhodanica	(26)
Crouzeliinae indet. (Mörgen)	(27, 28)
Egarapithecus narcisoi	(29)
Fanchangia jini	(4)
Laccopithecus robustus	(30)
Plesiopliopithecus lockeri	authors' own data from casts
Dendropithecus macinnesi	(31); authors' own data from casts
Micropithecus clarki	(32, 33); authors' own data from casts
Simiolus enjiessi	(34–36); authors' own data from casts

Supplementary Table 3. Dental BM estimates of *Pliobates cataloniae.* When the two antimeres of the same tooth locus were available for a single individual, average of the R and L MD and BL measurements (in mm) were employed to compute A.

Specimen	Tooth	A (mm^2)	BM (kg)	50% CI	
IPS58443 (holotype)	M1	29.50	3.95	3.66	4.24
IPS43758	M1	30.50	4.19	3.90	4.48
IPS44014 (male)	M1	33.92	5.06	4.76	5.35
IPS100379	M1	31.72	4.49	4.20	4.78
M1 average		31.41	4.41	3.66	5.35
IPS58443 (holotype)	M2	34.45	4.84	4.56	5.13
IPS43758	M2	39.44	5.79	5.50	6.08
IPS94888	M2	38.50	5.61	5.32	5.89
IPS100384	M2	45.75	7.04	6.75	7.33
M2 average		39.54	5.81	4.56	7.33
IPS58443 (holotype)	M3	29.80	5.42	5.15	5.68
IPS43936	m1	28.67	5.46	5.25	5.67
IPS43936	m2	33.00	5.48	5.24	5.71
Average for all tooth loci			5.31	3.66	7.33

Abbreviations: A = occlusal tooth square area (in mm^2); BL = (maximum) buccolingual breadth (in mm); BM = body mass; CI = confidence interval; L = left; MD = mesiodistal length (in mm); R = right.

Supplementary Table 4. Main postcranial features of *Pliobates* based on Alba et al. (2015), distinguishing between primitive (plesiomorphic) and derived (purportedly synapomorphic) features compared with crown hominoids.

Plesiomorphic features	
Proximal humerus	Anteriorly straight and somewhat proximally retroflexed shaft
Distal humerus	Poorly defined trochlear lateral keel and lack of spool-shaped trochlea
Proximal ulna	Narrow trochlear notch without a median keel
Pisiform	Small facet for the styloid process
Derived features	
Distal humerus	Moderately globular capitulum; no entepicondylar foramen; well-developed zona conoidea
Proximal radius	Laterally facing bicipital tuberosity; slightly tilted and almost circular head with reduced lateral lip and beveled surface for the zona conoidea
Distal ulna	Expanded head with a two-faceted semilunar articulation; more developed ulnar fovea; slender and hook-like styloid process
Capitate	Facet for the second metacarpal divided by a deep ligamentary notch
Triquetrum	Small triquetrum relative to hamate size

Supplementary Table 5. Comparative taxa used in this study.

Superfamily	Family	Taxon	Chronostratigraphic range	Geographical distribution	Material	Main references
Pliopithecoidea	Dionysopithecidae	*Dionysopithecus shuangouensis*	~19–18 Ma	China	Dental	(17)
Pliopithecoidea	Dionysopithecidae	*Platodontopithecus jianghuaiensis*	~19–18 Ma	China	Dental, postcranial	(17)
Pliopithecoidea	Pliopithecidae	*Epipliopithecus vindobonensis*	~14.7 Ma	Slovakia	Cranial, dental, postcranial	(19, 37–38)
Pliopithecoidea	Pliopithecidae	*Pliopithecus antiquus*	14.4–11.2 Ma	France	Dental, postcranial	(18, 19, 39–40)
Pliopithecoidea	Pliopithecidae	*Pliopithecus bii*	14.4–12.4 Ma	China	Dental	(20)
Pliopithecoidea	Pliopithecidae	*Pliopithecus canmatensis*	~11.9–11.7 Ma	Spain	Cranial, dental, postcranial	(21, 41)
Pliopithecoidea	Pliopithecidae	*Pliopithecus piveteaui*	16.4–14.4 Ma	France	Dental	(19, 22, 39, 42)
Pliopithecoidea	Pliopithecidae	*Pliopithecus platyodon*	16.4–12.4 Ma	Switzerland and Austria	Dental	(39, 43–44)
Pliopithecoidea	Pliopithecidae	*Pliopithecus zhangxiangi*	14.4–12.4 Ma	China	Cranial, dental	(23)
Pliopithecoidea	Crouzeliidae	*Fanchangia jini*	~18–17 Ma	China	Dental	(4)
Pliopithecoidea	Crouzeliidae	*Krishnapithecus krishnai*	~9 Ma	India	Dental	(5, 24)
Pliopithecoidea	Crouzeliidae	*Laccopithecus robustus*	7 Ma	China	Cranial, dental, postcranial	(30, 45–47)
Pliopithecoidea	Crouzeliidae	*Egarapithecus narcisoi*	~9.1 Ma	Spain	Dental	(29)
Pliopithecoidea	Crouzeliidae	*Anapithecus hernyaki*	~10 Ma	Austria, Germany, and Hungary	Cranial, dental, postcranial	(39, 48–51)
Pliopithecoidea	Crouzeliidae	*Barberapithecus huerzeleri*	~11.2 Ma	Spain	Dental, postcranial	(25, 52)
Pliopithecoidea	Crouzeliidae	Crouzeliinae indet. (Mörgen)	11.2–9.9 Ma	Germany	Dental	(27–28)
Pliopithecoidea	Crouzeliidae	*Plesiopliopithecus lockeri*	14.4–12.4 Ma	Austria	Dental	(39, 53–55)
Pliopithecoidea	Crouzeliidae	*Crouzelia auscitanensis*	14.4–13.8 Ma	France	Dental, postcranial	(26, 39)
Pliopithecoidea	Crouzeliidae	*Crouzelia rhodanica*	12.4–11.9 Ma	France	Dental	(26, 39)

Incertae sedis	Dendropithecidae	*Dendropithecus macinnesi*	~20.3–17 Ma	Kenya	Cranial, dental, postcranial	(31, 56–58)
Incertae sedis	Dendropithecidae	*Micropithecus clarki*	~20.5–19 Ma	Uganda	Cranial, dental, postcranial	(32–33, 57–59)
Incertae sedis	Dendropithecidae	*Simiolus enjiessi*	~21–17.5 Ma	Kenya	Cranial, dental, postcranial	(34–36, 58, 60)
Cercopithecoidea	Victoriapithecidae	*Victoriapithecus macinnesi*	~19.5–12.1 Ma	Kenya and Uganda	Cranial, dental, postcranial	(61–66)
Hominoidea	Proconsulidae	*Ekembo heseloni*	~18.5–16 Ma	Kenya	Cranial, dental, postcranial	(58, 67–68)
Saadanioidea	Saadaniidae	*Saadanius hijazensis*	29–28 Ma	Saudi Arabia	Cranial, dental	(69)

Supplementary Table 6. Digital object identifiers (DOIs) for outer enamel surface 3D models of *Pliobates cataloniae* teeth available from MorphoSource after this work. 3D models of the enamel dentine junction have been uploaded to MorphoSource but are embargoed until ongoing research of tooth endostructural morphology is published.

Catalogue No.	Tooth	Digital objet identifier (DOI)	
		OES	EDJ
IPS58443.1	R M2	https://doi.org/10.17602/M2/M494194	https://doi.org/10.17602/M2/M494191
IPS58443.2	L M3	https://doi.org/10.17602/M2/M494200	https://doi.org/10.17602/M2/M494197
IPS42977	L DP4	https://doi.org/10.17602/M2/M493937	https://doi.org/10.17602/M2/M493934
IPS42977	L P4 germ	https://doi.org/10.17602/M2/M493943	https://doi.org/10.17602/M2/M493940
IPS43936	L i1 germ	https://doi.org/10.17602/M2/M494140	https://doi.org/10.17602/M2/M494137
IPS43936	L i2 germ	https://doi.org/10.17602/M2/M494143	https://doi.org/10.17602/M2/M494146
IPS43936	L c1 germ	https://doi.org/10.17602/M2/M494122	https://doi.org/10.17602/M2/M494119
IPS43936	L p3 germ	https://doi.org/10.17602/M2/M494164	https://doi.org/10.17602/M2/M494161
IPS43936	L p4 germ	https://doi.org/10.17602/M2/M494170	https://doi.org/10.17602/M2/M494684
IPS43936	L m1 germ	https://doi.org/10.17602/M2/M494152	https://doi.org/10.17602/M2/M494149
IPS43936	L m2 germ	https://doi.org/10.17602/M2/M494158	https://doi.org/10.17602/M2/M494155
IPS43936	L dp3	https://doi.org/10.17602/M2/M494128	https://doi.org/10.17602/M2/M494125
IPS43936	L dp4	https://doi.org/10.17602/M2/M494134	https://doi.org/10.17602/M2/M494131
IPS44014	L P3	https://doi.org/10.17602/M2/M494182	https://doi.org/10.17602/M2/M494179
IPS44014	L P4	https://doi.org/10.17602/M2/M494188	https://doi.org/10.17602/M2/M494185
IPS44014	L M1	https://doi.org/10.17602/M2/M494173	https://doi.org/10.17602/M2/M494176
IPS93524	R p4	https://doi.org/10.17602/M2/M494206	https://doi.org/10.17602/M2/M494203
IPS94886	R m2 germ	https://doi.org/10.17602/M2/M494212	https://doi.org/10.17602/M2/M494209
IPS94888	L M2 germ	https://doi.org/10.17602/M2/M494218	https://doi.org/10.17602/M2/M494215
IPS100379	R M1	https://doi.org/10.17602/M2/M494224	https://doi.org/10.17602/M2/M494221
IPS100384	L M2 germ	https://doi.org/10.17602/M2/M494230	https://doi.org/10.17602/M2/M494227
IPS106878	L dp4 crown	https://doi.org/10.17602/M2/M494236	https://doi.org/10.17602/M2/M494233

Abbreviations: L = left; R = right.

Supplementary References

1. Alba, D. M. et al. Miocene small-bodied ape from Eurasia sheds light on hominoid evolution. *Science* **350**, aab2625 (2015).
2. Pugh, K. D. Phylogenetic analysis of Middle-Late Miocene apes. *J. Hum. Evol.* **165**, 103140 (2022).
3. Urciuoli, A. & Alba, D. M. Systematics of Miocene apes: State of the art of a neverending controversy. *J. Hum. Evol.* **175**, 103309 (2023).
4. Harrison, T. et al. A new genus of pliopithecoid from the late Early Miocene of China and its implications for understanding the paleozoogeography of the Pliopithecoidea. *J. Hum. Evol.* **145**, 102838 (2020).
5. Sankhyan, A. R., Kelley, J. & Harrison, T. A highly derived pliopithecoid from the Late Miocene of Haritalyangar, India. *J. Hum. Evol.* **105**, 1–12 (2017).
6. Gagnaison, C., Castillo, L., Grugier, O. & Renou, J.-C. Une hémimandibule de *Pliopithecus piveteaui* dans le Miocène de Contres (41, France). *Symbioses* **16**, 26–29 (2006).
7. Delson, E. & Andrews, P. Evolution and interrelationships of the catarrhine primates. in *Phylogeny of the Primates: A Multidisciplinary Approach* (eds. Luckett, W. P. & Szalay, F. S.) 405–446 (Plenum Press, 1975).
8. Harrison, T. The phylogenetic relationships of the early catarrhine primates: a review of the current evidence. *J. Hum. Evol.* **16**, 41–80 (1987).
9. Nengo, I. et al. New infant cranium from the African Miocene sheds light on ape evolution. *Nature* **548**, 169–174 (2017).
10. Ji, X. et al. The earliest hylobatid from the Late Miocene of China. *J. Hum. Evol.* **171**, 103251 (2022).
11. Almécija, S., Tallman, N., Sallam, H. M., Fleagle, J. G., Hammond, A. S. & Seiffert, E. R. Early anthropoid femora reveal divergent adaptive trajectories in catarrhine hind-limb evolution. *Nat. Comm.* **10**, 4778 (2019).
12. Almécija, S., Hammond, A. S., Thompson, N. E., Pugh, K. D., Moyà-Solà, S. & Alba, D. M. Fossil apes and human evolution. *Science* **372** eabb4363 (2021). DOI: 10.1126/science.abb436
13. Fleagle, J. G. *Primate Adaptation and Evolution, Third Edition* (Academic Press, 2013). https://doi.org/10.1016/C2009-0-01979-5

14. Casanovas-Vilar, I. et al. The Miocene mammal record of the Vallès-Penedès Basin (Catalonia). *C. R. Palevol* **15**, 791–812 (2016).
15. Alba, D. M., Moyà-Solà, S., Robles, J. M. & Galindo, J. Brief communication: The oldest pliopithecid record in the Iberian Peninsula based on new material from the Vallès-Penedès Basin. *Am. J. Phys. Anthropol.* **147**, 135–140 (2012).
16. Alba, D. M. et al. A revised (earliest Vallesian) age for the hominoid-bearing locality of Can Mata 1 based on new magnetostratigraphic and biostratigraphic data from Abocador de Can Mata (Vallès-Penedès Basin, NE Iberian Peninsula). *J. Hum. Evol.* **170**, 103237 (2022).
17. Harrison, T. & Gu, Y. Taxonomy and phylogenetic relationships of Early Miocene catarrhines from Sihong, China. *J. Hum. Evol.* **37**, 225–277 (1999).
18. Hürzeler, J. Contribution à l'odontologie et à la phylogénèse du genre *Pliopithecus* Gervais. *Ann. Paleontol.* **40**, 5–63 (1954).
19. Zapfe, H. Die Primatenfunde aus der miozänen Spaltenfüllung von Neudorf an der March (Děvinská Nová Ves), Tschechoslowakei. *Schweizer. Palaeontol. Abh.* **78**, 4–293 (1961a).
20. Wu, W.-Y., Meng, J., & Ye, J. The discovery of *Pliopithecus* from northern Junggar Basin, Xinjiang. *Vert. PalAs.* **41**, 76–86. (2003).
21. Alba, D. M. et al. A new species of *Pliopithecus* Gervais, 1849 (Primates: Pliopithecidae) from the Middle Miocene (MN8) of Abocador de Can Mata (els Hostalets de Pierola, Catalonia, Spain). *Am. J. Phys. Anthropol.* **141**, 52–75 (2010).
22. Ginsburg, L. Le Pliopithèque des faluns Helvétiens de la Touraine et de l'Anjou. in *Problèmes Actuels de Paléontologie (Évolution des Vertébrés). Paris, 4-9 Juin, 1973.* 877–886 (CNRS, Paris, 1975).
23. Harrison, T., Delson, E. & Guan, J. A new species of *Pliopithecus* from the Middle Miocene of China and its implications for early catarrhine zoogeography. *J. Hum. Evol.* **21**, 329–361 (1991).
24. Chopra, S. R. K. & Kaul, S. A new species of *Pliopithecus* from the Indian Sivaliks. *J. Hum. Evol.* **8**, 475–477 (1979).
25. Alba, D. M. & Moyà-Solà, S. A new pliopithecid genus (Primates, Pliopithecoidea) from Castell de Barberà (Vallès-Penedès Basin, Catalonia, Spain). *Am. J. Phys. Anthropol.* **147**, 88–112 (2012).
26. Ginsburg, L & Mein, P. *Crouzelia rhodanica*, nouvelle espèce de primate catarhinien, et essai sur la position systématique des Pliopithecidae. *Bull. Mus. Natl. Hist. Nat.* **2**, 57–85 (1980).

27. Heißig, K. *Ein Pliopithecide aus dem Vallesium* (*MN 9) der Oberen* Süßwassermolasse Bayerns. *Ber. naturwiss. Ver. Schwaben* **103**, 1–2 (1999).

28. Seehuber, *U. Litho- und biostratigraphische Untersuchungen in der Oberen Süßwassermolasse in der Umgebung von Kirchheim in Schwaben* (Ludwig-Maximilians-Universität München, München, Germany. 2008).

29. Moyà-Solà, S., Köhler, M. & Alba, D. M. *Egarapithecus narcisoi*, a new genus of Pliopithecidae (Primates, Catarrhini) from the Late Miocene of Spain. *Am. J. Phys. Anthropol.* **114**, 312–324 (2001).

30. Pan, Y., Waddle, D. M. & Fleagle, J. G. Sexual dimorphism in *Laccopithecus robustus*, a Late Miocene hominoid from China. *Am. J. Phys. Anthropol.* **79**, 137–158 (1989).

31. Andrews, P. A revision of the Miocene Hominoidea of East Africa. *Bull. Br. Mus. Nat. Hist. Geol.* **30**, 85–224 (1978).

32. Pickford, M., Musalizi, S., Senut, B., Gommery, D. & Musiime, E. Small apes from the early Miocene of Napak, Uganda. *Geo-Pal Uganda* **3**, 1–111 (2010).

33. Pickford, M., Senut, B., Gommery, D., Musalizi, S. & Ssebuyungo, C. Revision of smaller-bodied anthropoids from Napak, early Miocene, Uganda: 2011-2020 collections. *Münchner Geowiss. Abh.* **51**, 1–135 (2021).

34. Leakey, R. E. F & Leakey, M. G. A. new Miocene small-bodied ape from Kenya. *J. Hum. Evol.* **16**, 369–387 (1987).

35. Harrison, T. A reassessment of the taxonomic and phylogenetic affinities of the fossil catarrhines from Fort Ternan, Kenya. *Primates* **33**, 501–522 (1992).

36. Pickford, M., Senut, B., Gommery, D., Musalizi, S. & Musiime, E. Revision of the Miocene Hominoidea from Moroto I and II, Uganda. *Geo-Pal Uganda* **10**, 1–32 (2017).

37. Zapfe, H. & Hürzeler, J. Die Fauna der miozänen Spaltenfüllung von Neudorf an der March (ČSR.). *Primates. Sitzungsber. Öst. Akad. Wiss. Math. Naturwiss. Kl.* **166**, 113–123 (1957).

38. Zapfe, H. The skeleton of *Pliopithecus* (*Epipliopithecus*) *vindobonensis* Zapfe and Hürzeler. *Am. J. Phys. Anthropol.* **16**, 441–457 (1958).

39. Begun, D. R. The Pliopithecoidea. in *The Primate Fossil Record* (ed. Hartwig, W. C.) 221–240 (Cambridge University Press, 2002).

40. Senut, B. Les restes post-crâniens des Pliopithecidae (Primates) de Sansan. *Mém. Mus. Natl. Hist. Nat.* **203**, 535–558 (2012).

41. Alba, D. M. & Moyà-Solà, S. New fossil remains of *Pliopithecus canmatensis* from Abocador de Can Mata, and their implications for the taxonomic validity and phylogenetic position of Epipliopithecus (Primates, Pliopithecidae). *Am. J. Phys. Anthropol.* **153**, 64 (2014).

42. Gagnaison, C., Castillo, L., Grugier, O. & Renou, J.-C. Une hémimandibule de *Pliopithecus piveteaui* dans le Miocène de Contres (41, France). *Symbioses* **16**, 26–29. (2006).

43. Ginsburg, L. Chronology of the European pliopithecids. in *Primate Evolution* (eds. Else, J. G. & Ice, P. C.) 47–57 (Cambridge University Press, 1986).

44. Steininger, F. Dating the Paratethys Miocene hominoid record. in *Primate Evolution* (eds. Else, J. G. & Ice, P. C.) 71–84 (Cambridge University Press, 1986).

45. Wu, R. & Pan, Y. A late Miocene gibbon-like primate from Lufeng, Yunnan Province. *Acta Anthropol. Sin.* **3**, 185–194. (1984).

46. Wu, R. & Pan, Y. Preliminary observation on the cranium of *Laccopithecus robustus* from Lufeng, Yunnan with reference to its phylogenetic relationship. *Acta Anthropol. Sin*. **4**, 7–12. (1985).

47. Meldrum, D. J. & Pan, Y. Manual proximal phalanx of *Laccopithecus robustus* from the latest Miocene site of Lufeng. *J. Hum. Evol.* **17**, 719–731 (1988).

48. Begun, D. R. Catarrhine phalanges from the Late Miocene (Vallesian) of Rudabánya, Hungary. *J. Hum. Evol.* **17**, 413–438 (1988).

49. Begun, D. R. New catarrhine phalanges from Rudabánya (Northeastern Hungary) and the problem of parallelism and convergence in hominoid postcranial morphology. *J. Hum. Evol.* **24**, 373–402 (1993).

50. Kordos, L. & Begun, D. R. Femora of *Anapithecus* from Rudabànya. *Am. J. Phys. Anthropol.* **108** (S28), 173 (1999).

51. Kordos, L. & Begun, D. R. Primates from Rudabánya: allocation of specimens to individuals, sex and age categories. *J. Hum. Evol.* **40**, 17–39 (2001).

52. Arias-Martorell, J., Almécija, S., Urciuoli, A., Nakatsukasa, M., Moyà-Solà, S. & Alba, D. M. A proximal radius of *Barberapithecus huerzeleri* from Castell de Barberà: Implications for locomotor diversity among pliopithecoids. *J. Hum. Evol.* **157**, 103032 (2021).

53. Zapfe, H. A new fossil anthropoid from the Miocene of Austria. *Curr. Anthropol*. **1**, 428–429. (1960).

54. Zapfe, H. Ein Primatenfund aus der miozänen Molasse von Oberösterreich. *Z. Morphol. Anthropol*. **51**, 247–267. (1961b).

55. Alba, D. M. & Berning, B. On the holotype and original description of the pliopithecid *Plesiopliopithecus lockeri* (Zapfe, 1960). *J. Hum. Evol*. **65**, 338–340. (2013).

56. Le Gros Clark, W. E. & Thomas, D. P. *Associated jaws and limb bones of* Limnopithecus macinnesi, *Fossil Mammals Afr. 3* (British Museum, 1951).

57. Harrison, T. *Small-bodied apes from the Miocene of East Africa* (University College London, London, 1982).

58. Harrison, T. Dendropithecoidea, Proconsuloidea and Hominoidea (Catarrhini, primates). in *Cenozoic Mammals of Africa* (eds. Werdelin, L. & Sanders, W. J.) 429–469 (California Press, 2010).

59. Fleagle, J. G. & Simons, E. L. *Micropithecus clarki*, a small ape from the Miocene of Uganda. *Am. J. Phys. Anthropol.* **49**, 427–440 (1978).

60. Rossie, J. B., Gutierrez, M. M. & Goble, E. Fossil forelimbs of *Simiolus* from Moruorot, Kenya. *Am. J. Phys. Anthropol.* **147** (S54), 252. (2012).

61. Benefit, B. R. The permanent dentition and phylogenetic position of *Victoriapithecus* from Maboko Island, Kenya. *J. Hum. Evol.* **25**, 83–172 (1993).

62. Benefit, B. R. *Victoriapithecus*: The key to Old World monkey and Catarrhine origins. *Evol. Anthropol*. **7**, 155–174 (1999).

63. Benefit, B. R. & McCrossin, M. L. Earliest known Old World monkey skull. *Nature*, **388**, 368–371 (1997).

64. Benefit, B. R. & McCrossin, M. L. The Victoriapithecidae, Cercopithecoidea. in *The Primate Fossil Record* (ed. Hartwig, W. C.) 241–253 (Cambridge University Press, 2002).

65. Jablonski, N. G. & Frost, S. Cercopithecoidea. in *Cenozoic Mammals of Africa* (eds. Werdelin, L. & Sanders, W. J.) 393–428 (California Press, 2010).

66. Pickford, M., Senut, B., Musalizi, S., Gommery, D. & Ssebuyungo, C. Early Miocene victoriapithecid monkey from Napak, Uganda. *Geo-Pal Uganda* **12**, 1–17 (2019).

67. Walker, A., Teaford, M. F., Martin, L. & Andrews, P. A new species of *Proconsul* from the Early Miocene of Rusinga/Mfangano Islands, Kenya. *J. Hum. Evol.* **25**, 43–56 (1993).

68. McNulty, K. P., Begun, D. R., Kelley, J., Manthi, F. K. & Mbua, E. N. A systematic revision of *Proconsul* with the description of a new genus of early Miocene hominoid. *J. Hum. Evol.* **84**, 42–61 (2015).

69. Zalmout, I. S. et al. New Oligocene primate from Saudi Arabia and the divergence of apes and Old World monkeys. *Nature* **466**, 360–365 (2017).
