# Supplementary material for: The Miocene primate Pliobates is a pliopithecoid
Source: Nat Commun. 2024 Apr 1;15:2822. doi: 10.1038/s41467-024-47034-9 (PMC10984959; doi:10.1038/s41467-024-47034-9)
Supplement: Supplementary file 3 — Description of Additional Supplementary Files [file 41467_2024_47034_MOESM3_ESM.pdf]

Description of Additional Supplementary Materials

Supplementary Data 1. Dental character statements employed in the dental-only cladistic analysis. Character number (in bold type) is followed by character and character state definition. 23 characters were continuous and discretized.

Supplementary Data 2. Taxon-character matrix of 95 characters and 27 taxa used in the dental-only cladistic analysis. Missing data are denoted by a question mark. Variable character states are denoted by capital letters: A=(0,1), B=(0,2), C=(0,3), D=(1,2). Parsimony uninformative characters are italicized. See character statements in Supplementary Data 1.

Supplementary Data 3. List of apomorphies of the clades recovered in the consensus tree from the 192 most parsimonious trees. Double arrows '==>' denote unambiguous changes, whereas single arrows '-->' denote ambiguous changes (i.e., including reversals). See character statements in Supplementary Data 1 and node numbers in Fig. 5.

Supplementary Data 4. Character statements employed in the additional cladistic analysis. Character number (in bold type) is followed by character and character state definition. 23 characters were continuous and discretized.

Supplementary Data 5. Taxon-character matrix of 381 characters and 22 taxa used for the additional cladistic analysis. Missing data are denoted by a question mark. Variable character states are denoted by capital letters: A=(0,1), B=(0,2), C=(0,3), D=(1,2). Parsimony uninformative characters are italicized. See character statements in Supplementary Data 4.
