# Supplementary material for: The Miocene primate Pliobates is a pliopithecoid
Source: Nat Commun. 2024 Apr 1;15:2822. doi: 10.1038/s41467-024-47034-9 (PMC10984959; doi:10.1038/s41467-024-47034-9)
Supplement: Supplementary file 4 — Supplementary Data 1 [file 41467_2024_47034_MOESM4_ESM.docx]

| **#001:** I1–I2 size heteromorphy: 0, low; 1, moderate; 2, marked. |
| --- |
| **#002:** I1–I2 lingual morphology: 0, lingual features (other than lingual cingulum, if present) absent or poorly-developed; 1, lingual features (basal bulge, pillar and/or crenulations) well developed. |
| **#003:** Lingual cingulum and crown base of I1: 0, narrow cingulum with slender crown base; 1, broad cingulum with bulging crown base. |
| **#004:** I2 morphology: 0, peg-shaped; 1, spatulate. |
| **#005:** Apicobasal height of I1 relative to mesiodistal length: 0, low-crowned; 1, high-crowned. |
| **#006:** Apicobasal height of I2 relative to mesiodistal length: 0, low-crowned; 1, high-crowned. |
| **#007:** i2 mesiodistal waisting: 0, absent; 1, present. |
| **#008:** i2 mesial margin: 0, straight to slightly inclined; 1, clearly inclined mesialward. |
| **#009:** i2 distal margin: 0, markedly angled; 1, rather straight. |
| **#010:** i2 distal prong: 0, absent to slightly developed; 1, well developed. |
| **#011:** Apicobasal height of i1 relative to mesiodistal length: 0, high-crowned; 1, very high-crowned. |
| **#012:** Apicobasal height of i2 relative to mesiodistal length: 0, high-crowned; 1, very high-crowned. |
| **#013:** Male C1 crown shape: 0, moderately compressed but not very high and not markedly dagger-like; 1, male upper canine crown very compressed, high, and dagger-like; 2, male upper canine crown stouter and less compressed. |
| **#014:** Male C1 sulcus: 0, sulcus not extending onto root; 1, sulcus extending onto root. |
| **#015:** Female c1 length/breadth index: 0, broad; 1, narrow. |
| **#016:** Female c1 apicobasal height relative to mesiodistal length: 0, low-crowned; 1, high-crowned. |
| **#017:** Mesiolingual cristid-lingual cingulid thickening in the female c1: 0, restricted angle to rounded angle; 1, slightly prominent angle; 2, prominent angle, cuspulid-like. |
| **#018:** Position of the Mesiolingual cristid-lingual cingulid junction in the female c1: 0, at about crown-midheight; 1, higher. |
| **#019:** P3 breadth/length index: 0, very broad; 1, broad. |
| **#020:** P3 length relative to P4 length: 0, P3 shorter to subequal in length to P4; 1, P3 longer than P4. |
| **#021:** P3 length relative to M2 length: 0, P3 markedly shorter than M2; 1, P3 moderately shorter than M2. |
| **#022:** P3 cusp heteromorphy: 0, strong with tall paracone; 1, reduced with tall paracone; 2, reduced with low paracone. |
| **#023:** P3 central fovea shape: 0, broader than long; 1, relatively narrower. |
| **#024:** P3 buccal wall shape: 0, clearly triangular; 1, subtriangular to slightly rhomboid; 2, clearly rhomboid. |
| **#025:** P3 buccal wall height in buccal view: 0, low; 1, high. |
| **#026:** P3 lingual cingulum: 0, conspicuously present in most instances; 1, usually absent or poorly developed. |
| **#027:** P3 paracone position along the crown buccolingual axis: 0, very peripheral; 1, not very peripheral. |
| **#028:** P4 occlusal contour: 0, (sub)triangular; 1, (sub)oval to (sub)elliptical. |
| **#029:** P4 breadth/length index: 0, very broad; 1, broad. |
| **#030:** P4 length relative to M2 length: 0, P4 markedly shorter than M2; 1, P4 moderately shorter than M2. |
| **#031:** P4 lingual cingulum: 0, conspicuously present in most instances; 1, usually absent or poorly developed. |
| **#032:** P4 central fovea shape: 0, broader than long; 1, relatively narrower. |
| **#033:** P4 paracone position along the crown buccolingual axis: 0, very peripheral; 1, not very peripheral. |
| **#034:** p3 sectoriality: 0, non-sectorial to poorly sectorial; 1, moderately sectorial; 2, markedly to strongly sectorial. |
| **#035:** p3 mesiobuccal honing face length: 0, long; 1, short. |
| **#036:** p3 mesiobuccal honing face inclination: 0, inclined; 1, steep. |
| **#037:** Metaconid on p3: 0, absent; 1, small; 2, prominent. |
| **#038:** p4 crown shape: 0, (sub)rectangular to (sub)elliptical; 1, more clearly (sub)oval. |
| **#039:** p4 breadth/length index: 0, very to slightly narrow; 1, slightly broader than long. |
| **#040:** p4 length relative to m2 length: 0, p4 markedly shorter than m2; 1, p4 moderately shorter than m2. |
| **#041:** p4 buccal cingulid: 0, shelf-like from mesial to distal; 1, clearly disrupted or limited to some portions of the crown. |
| **#042:** Bilophodont molars: 0, absent; 1, present. |
| **#043:** Molar cusp morphology: 0, rounded cusps; 1, pyramidal cusps; 2, pyramidal but somewhat compressed cusps; 3, very compressed cusps. |
| **#044:** Upper molar cusp height: 0, low cusps; 1, high cusps. |
| **#045:** (Lower) molar cusp peripheralization: 0, cusps not peripheral; 1, cusps moderately peripheral; 2, cusps very peripheral. |
| **#046:** Molar crest morphology: 0, blunt crests; 1, moderately sharp crests; 2, markedly sharp crests. |
| **#047:** M1–M2 waisting: 0, absent to slight; 1, moderate to marked. |
| **#048:** M1 length/breadth index: 0, very broad; 1, moderately broad. |
| **#049:** M2 length/breadth index: 0, very broad; 1, moderately broad. |
| **#050:** M3 length/breadth index: 0, very broad; 1, moderately broad. |
| **#051:** M1/M2 length ratio: 0, M1 moderately shorter than M2; 1, M1 slightly shorter to subequal in length to M2. |
| **#052:** M3/M2 length ratio: 0, M3 moderately shorter than M2; 1, M3 slightly shorter to subequal in length to M2. |
| **#053:** M1–M3 protoconule: 0, present and large; 1, present and small; 2, usually absent or poorly distinct. |
| **#054:** M1–M2 buccal cingulum: 0, well-developed; 1, variously present or discontinuous/poorly developed. |
| **#055:** M3 buccal cingulum: 0, well-developed; 1, poorly developed. |
| **#056:** M1–M2 lingual cingulum breadth: 0, very to moderately broad; 1, narrow. |
| **#057:** M1–M2 lingual cingulum distal development: 0, not surrounding the hypocone; 1, surrounding the hypocone or showing clear distal style(s). |
| **#058:** M3 lingual cingulum: 0, well-developed; 1, moderately developed; 2, poorly developed. |
| **#059:** Crista obliqua in upper molars: 0, present in all instances; 1, variably present. |
| **#060:** M2–M3 metacone size: 0, large; 1, small (compared to the protocone and paracone). |
| **#061:** M1–M2 hypocone size (relative to metacone and paracone): 0, similarly sized to slightly smaller; 1, much smaller. |
| **#062:** M3 hypocone size (relative to metacone and paracone): 0, large; 1, small; 2, absent or rudimentary. |
| **#063:** M1–M2 paracone buccal position relative to the metacone (more marked in the M2): 0, markedly buccal; 1, buccal. |
| **#064:** M1–M2 protocone distal position relative to the paracone: 0, clearly distal; 1, distal; 2, aligned. |
| **#065:** M3 protocone distal position relative to the paracone: 0, clearly distal; 1, distal; 2, aligned. |
| **#066:** M1–M2 hypocone lingual position relative to the protocone: 0, clearly more lingual; 1, almost aligned or lingual. |
| **#067:** M3 hypocone lingual position relative to the protocone: 0, aligned; 1, clearly lingual. |
| **#068:** M1–M2 buccal profile length: 0, moderately longer than the lingual profile; 1, markedly longer than the lingual profile. |
| **#069:** M1–M3 hypocone-metacone crista: 0, absent; 1, disrupted; 2, complete. |
| **#070:** M1–M2 prehypocrista: 0, present and meets protocone; 1, variably present (meets protocone); 2, present and meets crista obliqua. |
| **#071:** M1–M2 trigon basin: 0, as long as broad; 1, broader than long. |
| **#072:** M1–M2 distal fovea: 0, shorter than trigon; 1, clearly longer than trigon, at least in some molars. |
| **#073:** m1–m3 cusps: 0, medium-sized to extensive; 1, more discrete. |
| **#074:** m1–m3 mesial arm of pliopithecine triangle: 0, always absent; 1, variably present and/or incipient; 2, present in some molar, constituting a pliopithecine triangle. |
| **#075:** m1–m3 distal arm of pliopithecine triangle: 0, always absent; 1, variably present and/or poorly constituted; 2, well constituted. |
| **#076:** m1–m3 buccal cingulid width: 0, Ledge-like; 1, moderately broad; 2, narrow to absent. |
| **#077:** m1–m3 buccal cingulid extent: 0, continuous at least in some molar; 1, usually discontinuous; 2, limited to some stylids. |
| **#078:** m1–m3 mesial fovea: 0, very large, about as long as broad; 1, small to restricted, clearly broader than long. |
| **#079:** m1 breadth/length index: 0, very narrow; 1, moderately narrow. |
| **#080:** m2 breadth/length index: 0, very narrow; 1, moderately narrow. |
| **#081:** m1/m2 length ratio: 0, m1 moderately shorter than m2; 1, m1 only slightly shorter than m2. |
| **#082:** m1–m2 hypoconulid size: 0, present and well developed in all instances; 1, present in all instances but clearly reduced in size; 2, almost indistinguishable or absent. |
| **#083:** m1–m2 protoconid mesial position relative to the metaconid: 0, clearly more mesial; 1, almost transversely aligned. |
| **#084:** m1–m2 entoconid distal position relative to the hypoconid: 0, clearly more distal; 1, almost transversely aligned. |
| **#085:** m1–m2 hypoconulid position if present: 0, median or slightly buccal; 1, clearly buccal. |
| **#086:** m1–m3 crest pattern: 0, hypoprotocristid originates lingually from protoconid, cristid obliqua (if present) joins the protoconid distally; 1, hypoprotocristid originates distally or distolingually from protoconid, from where the inclined cristid obliqua joins protoconid, at least in m1; 2, hypoprotocristid originates distally from protoconid, the inclined cristid obliqua joins the hypoprotocristid-hypometacristid (Y crest pattern) at least in m1; 3, only cristid obliquid is present so that the mesial fovea communicates with the talonid. |
| **#087:** m1–m3 postcristid-hypoentocristid: 0, present; 1, absent. |
| **#088:** m1–m3 talonid secondary wrinkling: 0, present; 1, absent. |
| **#089:** m1–m2 distal fovea: 0, opens on the talonid basin (at least in some molars); 1, large to medium-sized and separated from the talonid basin; 2, small and separated from the talonid basin. |
| **#090:** m3 breadth/length index: 0, very narrow; 1, moderately narrow. |
| **#091:** m3/m2 length ratio: 0, m1 moderately shorter than m2; 1, m1 only slightly shorter than m2. |
| **#092:** p4 distal tubercles: 0, small or indistinct; 1, prominent. |
| **#093:** m3 entoconid relative size: 0, small; 1, large. |
| **#094:** m3 hypoconulid size: 0, present and well developed in all instances; 1, present in all instances but clearly reduced in size; 2, almost indistinguishable or absent. |
| **#095:** m3 hypoconulid position if present: 0, median or slightly buccal; 1, clearly buccal. |
