# Supplementary material for: The Miocene primate Pliobates is a pliopithecoid
Source: Nat Commun. 2024 Apr 1;15:2822. doi: 10.1038/s41467-024-47034-9 (PMC10984959; doi:10.1038/s41467-024-47034-9)
Supplement: Supplementary file 5 — Supplementary Data 2 [file 41467_2024_47034_MOESM5_ESM.docx]

0*0*00000000000000000000000*0*0*0*0*00*0000000000*0*00*0*0000000000000*0*0000000000000*0*000000000000000000000*0*

0*0*00000001111111111222222*2*2*2*2*33*3333333344*4*44*4*4444555555555*5*6666666666777*7*777777888888888899999*9*

Taxon/character 1*2*34567890123456789012345*6*7*8*9*01*2345678901*2*34*5*6789012345678*9*0123456789012*3*456789012345678901234*5*

*Aegyptopithecus* *zeuxis* 00?0??0001000000?1011010?0111101110101100000000000111010100000000010000000000111100000002110000

*Saadanius* *hijazensis* ???1?????????0?????10?1??????0?1?????????01??010000110100000000000100?10???????????????????????

*Pliobates* *cataloniae* 0?0???1101????0?20111011011101010????1??1021020110101110020112100001000000200000100101000??0???

*Micropithecus* *clarki* 00000101000100100110110101110100010001110000100111101110100102011010111100110111101100001010001

*Dendropithecus* *macinnesi* 100?110010?00011??101002111111000100001000111100111111101001010111001D1100110111001110001100101

*Simiolus* *enjiessi* 10????00101100110110000211110100010001010011110111011110100101011000200100121100101010001110101

*Pliopithecus* *platyodon* 0000????????00101010100101111100111101110011011001111111010111101000001002200111100001001?10011

*Pliopithecus* *antiquus* ??????1001????????1??00??11??????1110111001101???????????????????????0??02200111100000001010011

*Pliopithecus* *canmatensis* ?0?1??10011???????10100??111010001110111001101001?0?11?10?010?10?0?0001002100111100001001110011

*Pliopithecus* *zhanxiangi* ????????????00????101001011111001????????011010000111111010101101000001002200111000000001110011

*Pliopithecus* *piveteaui* ????????????????????????????0????????111001101???0???????????????????0??02200110000000001100011

*Pliopithecus bii* ?00??????????????????????????????????????01101??????????????????????????022001?1?0000000110?011

*Epipliopithecus* *vindobonensis* ?0000010011100????111001011111001111101100111110001101101001A1111A00101101100110000010001110011

*Dionysopithecus* *shuangouensis* ?00?0?1001?0???????????????1010001111?00?010010101111100100101111000100102200110000101001100011

*Platodontopithecus* *jianghuaiensis* ????????????11????00101??10?11???1111?00?010010001111100100101111000?00002200111001101001100011

*Egarapithecus* *narcisoi* ??????????????????0??011010??????0112001102?01??????????????????????????0??11?00100012001011011

*Barberapithecus* *huerzeleri* ?00?????????001110???????????????11110011021011000111101010111100001000002210010100100001010011

*Anapithecus* *hernyaki* 0010??0101??0010200010110101010100112011103102111?1?1100010001100001000002101010100002002011011

*Laccopithecus* *robustus* 0010??1101??10101000101??1010101011110011011011111111111010011100001000001021110100000001011011

*Plesiopliopithecus* *lockeri* ??????1101???????????????????????111000?002?02??????????????????????????0210000??10001002??0???

*Crouzelia* *auscitanensis* ?????????????????????????????????????001002?02??????????????????????????02110000110001100??0???

*Crouzelia* *rhodanica* ??????????????????????????????????????????2?02??????????????????????????021100?0?20001100??????

Crouzeliinae indet. (Mörgen) ???????????????????????????10?010????????021?101????11?10?011?10?0?10000???????????????????????

*Fanchangia* *jini* ?01110????0???0???10101??101010101111010001001111000111102010?100001000002221100000000002000011

*Krishnapithecus* *krishnaii* ?????????????????????????????????????????00?21???0??1?1??10??2??0???0???111211?0110003110??????

*Ekembo* heseloni 000010001011201000111001111111000100001000111101100101110200021111000B0000011111001000001110001

*Victoriapithecus* *macinnesi* 10001100110101011100011111100111120011010111111111002111021001022100A200000221110D1100111010001
