# Supplementary material for: The Miocene primate Pliobates is a pliopithecoid
Source: Nat Commun. 2024 Apr 1;15:2822. doi: 10.1038/s41467-024-47034-9 (PMC10984959; doi:10.1038/s41467-024-47034-9)
Supplement: Supplementary file 6 — Supplementary Data 3 [file 41467_2024_47034_MOESM6_ESM.docx]

| \| Branch Character Steps CI Change \| \| --- \| \| ------------------------------------------------------------------------------ \| \| node 18 <-> *Aegyptopithecus* *zeuxis* 11 1 0.250 1 <-> 0 \| \| 12 1 0.333 1 <-> 0 \| \| 15 1 0.250 1 <-> 0 \| \| 19 1 0.250 1 <-> 0 \| \| 24 1 1.000 1 <-> 0 \| \| 26 1 1.000 1 <-> 0 \| \| 29 1 0.143 0 <-> 1 \| \| 33 1 0.200 0 <-> 1 \| \| 40 1 0.200 1 <-> 0 \| \| 43 1 0.500 1 <=> 0 \| \| 44 1 0.250 1 <-> 0 \| \| 57 1 0.250 0 <=> 1 \| \| 71 1 0.200 1 <=> 0 \| \| 81 1 0.143 0 <-> 1 \| \| 89 1 0.333 1 <-> 2 \| \| 95 1 1.000 1 <-> 0 \| \| node 18 --> *Saadanius* *hijazensis* 4 1 0.333 0 ==> 1 \| \| 21 1 0.333 1 ==> 0 \| \| 30 1 1.000 1 ==> 0 \| \| 47 1 0.167 0 ==> 1 \| \| 51 1 0.200 1 ==> 0 \| \| node 18 --> node 17 20 1 0.250 1 ==> 0 \| \| 23 1 0.250 1 ==> 0 \| \| 32 1 0.333 1 ==> 0 \| \| 46 1 0.500 0 ==> 1 \| \| 54 1 1.000 0 ==> 1 \| \| 56 1 0.167 0 --> 1 \| \| 60 1 0.333 0 --> 1 \| \| 62 1 0.400 0 ==> 1 \| \| 65 1 0.667 0 ==> 1 \| \| 67 1 0.500 1 ==> 0 \| \| node 17 --> node 12 7 1 0.500 0 ==> 1 \| \| 17 1 0.500 0 --> 1 \| \| 18 1 0.500 1 ==> 0 \| \| 35 1 1.000 0 ==> 1 \| \| 58 1 0.333 0 --> 1 \| \| 63 1 0.500 0 ==> 1 \| \| 74 1 0.500 0 ==> 2 \| \| 75 1 0.286 0 ==> 2 \| \| 94 1 1.000 0 ==> 1 \| \| node 12 --> node 10 5 1 0.333 0 --> 1 \| \| 11 1 0.250 1 --> 0 \| \| 32 1 0.333 0 --> 1 \| \| 48 1 0.200 0 --> 1 \| \| 49 1 0.250 0 --> 1 \| \| 65 1 0.667 1 --> 0 \| \| 68 1 1.000 0 --> 1 \| \| 71 1 0.200 1 --> 0 \| \| 81 1 0.143 0 --> 1 \| \| 90 1 0.333 1 ==> 0 \| \| node 10 --> node 9 8 1 0.500 0 ==> 1 \| \| 23 1 0.250 0 ==> 1 \| \| 37 1 0.333 0 --> 1 \| \| 38 1 0.200 1 ==> 0 \| \| 39 1 0.167 1 --> 0 \| \| 41 1 0.333 0 --> 1 \| \| 80 1 0.200 1 ==> 0 \| \| node 9 --> node 4 16 1 0.333 0 --> 1 \| \| 20 1 0.250 0 --> 1 \| \| 43 1 0.500 1 ==> 2 \| \| 61 1 0.500 0 ==> 1 \| \| 78 1 0.500 1 ==> 0 \| \| 84 1 0.167 0 --> 1 \| \| node 4 --> node 3 15 1 0.250 1 --> 0 \| \| 17 1 0.500 1 --> 2 \| \| 37 1 0.333 1 --> 0 \| \| 46 1 0.500 1 ==> 2 \| \| 52 1 0.250 1 --> 0 \| \| 56 1 0.167 1 --> 0 \| \| 58 1 0.333 1 --> 2 \| \| 62 1 0.400 1 --> 2 \| \| 79 1 0.250 1 ==> 0 \| \| 86 1 0.500 0 ==> 1 \| \| 89 1 0.333 1 ==> 0 \| \| node 3 --> *Pliobates* *cataloniae* 38 1 0.200 0 ==> 1 \| \| 74 1 0.500 2 ==> 0 \| \| node 3 --> node 2 41 1 0.333 1 --> 0 \| \| 75 1 0.286 2 ==> 1 \| \| 82 1 0.600 0 ==> 1 \| \| 84 1 0.167 1 --> 0 \| \| node 2 --> *Plesiopliopithecus* *lockeri* 89 1 0.333 0 ==> 2 \| \| node 2 --> node 1 76 1 0.250 0 ==> 1 \| \| 87 1 0.333 0 ==> 1 \| \| node 1 --> *Crouzelia* *rhodanica* 82 1 0.600 1 ==> 2 \| \| node 4 --> *Barberapithecus* *huerzeleri* 47 1 0.167 0 ==> 1 \| \| 48 1 0.200 1 ==> 0 \| \| 49 1 0.250 1 --> 0 \| \| 55 1 0.500 1 ==> 0 \| \| 76 1 0.250 0 ==> 1 \| \| node 9 --> node 8 3 1 1.000 0 ==> 1 \| \| 27 1 0.500 1 ==> 0 \| \| 47 1 0.167 0 ==> 1 \| \| 76 1 0.250 0 --> 2 \| \| 77 1 0.500 0 ==> 1 \| \| node 8 --> *Fanchangia* *jini* 4 1 0.333 0 ==> 1 \| \| 15 1 0.250 1 ==> 0 \| \| 39 1 0.167 0 --> 1 \| \| 40 1 0.200 1 ==> 0 \| \| 41 1 0.333 1 --> 0 \| \| 44 1 0.200 1 ==> 0 \| \| 51 1 0.200 1 ==> 0 \| \| 52 1 0.250 1 ==> 0 \| \| 58 1 0.333 1 --> 2 \| \| 79 1 0.250 1 ==> 0 \| \| 81 1 0.143 1 ==> 0 \| \| 89 1 0.333 1 ==> 2 \| \| 91 1 0.200 1 ==> 0 \| \| node 8 --> node 7 19 1 0.250 1 ==> 0 \| \| 60 1 0.333 1 ==> 0 \| \| 75 1 0.286 2 ==> 1 \| \| 92 1 1.000 0 ==> 1 \| \| node 7 --> node 5 13 1 0.667 0 --> 1 \| \| 61 1 0.500 0 --> 1 \| \| 74 1 0.500 2 ==> 1 \| \| node 5 --> *Krishnapithecus* *krishnai* 43 1 0.500 1 ==> 0 \| \| 45 1 0.667 0 ==> 2 \| \| 62 1 0.400 1 ==> 2 \| \| 73 1 1.000 0 ==> 1 \| \| 82 1 0.600 0 ==> 1 \| \| 86 1 0.500 0 ==> 3 \| \| 87 1 0.333 0 ==> 1 \| \| 88 1 0.500 0 ==> 1 \| \| 89 1 0.333 1 ==> 0 \| \| node 5 --> *Laccopithecus* *robustus* 50 1 0.200 0 ==> 1 \| \| 75 1 0.286 1 ==> 0 \| \| node 7 --> node 6 7 1 0.500 1 --> 0 \| \| 17 1 0.500 1 --> 2 \| \| 34 1 1.000 1 ==> 0 \| \| 37 1 0.333 1 ==> 2 \| \| 43 1 0.429 1 --> 2 \| \| 55 1 0.333 1 --> 0 \| \| 56 1 0.167 1 --> 0 \| \| 76 1 0.250 2 --> 0 \| \| 78 1 0.500 1 --> 0 \| \| 86 1 0.500 0 ==> 2 \| \| node 6 --> *Egarapithecus* *narcisoi* 76 1 0.250 0 --> 1 \| \| 79 1 0.250 1 ==> 0 \| \| 85 1 0.333 0 ==> 1 \| \| node 6 --> *Anapithecus* *hernyaki* 39 1 0.167 0 --> 1 \| \| 43 1 0.429 2 --> 3 \| \| 46 1 0.500 1 ==> 2 \| \| 89 1 0.333 1 ==> 2 \| \| node 12 --> node 11 12 1 0.333 1 --> 0 \| \| 13 1 0.667 0 --> 1 \| \| 14 1 0.500 0 --> 1 \| \| 19 1 0.250 1 --> 0 \| \| 23 1 0.250 0 --> 1 \| \| 27 1 0.500 1 --> 0 \| \| 37 1 0.333 0 ==> 1 \| \| 39 1 0.167 1 ==> 0 \| \| 40 1 0.200 1 ==> 0 \| \| 44 1 0.250 1 ==> 0 \| \| 50 1 0.200 0 ==> 1 \| \| 55 1 0.333 1 ==> 0 \| \| 56 1 0.167 1 ==> 0 \| \| 57 1 0.250 0 ==> 1 \| \| 58 1 0.333 1 --> 0 \| \| 64 1 0.500 0 ==> 1 \| \| 69 1 0.600 0 --> 1 \| \| 71 1 0.200 1 ==> 0 \| \| 84 1 0.167 0 ==> 1 \| \| 86 1 0.500 0 ==> 1 \| \| 91 1 0.200 1 ==> 0 \| \| node 11 --> *Dionysopithecus* *shuangouensis* 48 1 0.200 0 ==> 1 \| \| 72 1 0.333 0 ==> 1 \| \| 80 1 0.200 1 ==> 0 \| \| node 11 --> *Platodonpithecus* *jianghuaiensis* 29 1 0.143 0 --> 1 \| \| 83 1 0.500 0 ==> 1 \| \| node 12 --> *Epipliopithecus* *vindobonensis* 20 1 0.250 0 ==> 1 \| \| 29 1 0.143 0 --> 1 \| \| 33 1 0.200 0 --> 1 \| \| 37 1 0.333 0 ==> 1 \| \| 38 1 0.200 1 ==> 0 \| \| 45 1 0.667 0 ==> 1 \| \| 47 1 0.167 0 ==> 1 \| \| 53 1 0.667 1 ==> 0 \| \| 56 1 0.167 1 ==> 0 \| \| 57 1 0.250 0 ==> 1 \| \| 58 1 0.333 1 --> 0 \| \| 61 1 0.500 0 --> A \| \| 64 1 0.500 0 ==> 1 \| \| 66 1 0.667 0 --> A \| \| 69 1 0.600 0 ==> 1 \| \| 72 1 0.333 0 ==> 1 \| \| 74 1 0.500 2 ==> 1 \| \| 75 1 0.286 2 ==> 1 \| \| 80 1 0.200 1 ==> 0 \| \| 85 1 0.333 0 ==> 1 \| \| node 12 --> *Pliopithecus* *bii* 91 1 0.200 1 ==> 0 \| \| node 12 --> *Pliopithecus* *zhanxiangi* 29 1 0.143 0 --> 1 \| \| 33 1 0.200 0 --> 1 \| \| 53 1 0.400 1 ==> 0 \| \| node 12 --> *Pliopithecus* *piveteaui* 80 1 0.200 1 ==> 0 \| \| 91 1 0.200 1 ==> 0 \| \| node 12 --> *Pliopithecus* *platyodon* 29 1 0.143 0 --> 1 \| \| 33 1 0.200 0 --> 1 \| \| 47 1 0.167 0 ==> 1 \| \| 50 1 0.200 0 ==> 1 \| \| 61 1 0.500 0 ==> 1 \| \| 81 1 0.143 0 --> 1 \| \| 86 1 0.500 0 ==> 1 \| \| node 12 --> *Pliopithecus* *canmatensis* 4 1 0.333 0 ==> 1 \| \| 49 1 0.250 0 ==> 1 \| \| 51 1 0.200 1 ==> 0 \| \| 75 1 0.286 2 ==> 1 \| \| 81 1 0.143 0 --> 1 \| \| 86 1 0.500 0 ==> 1 \| \| node 17 --> node 16 5 1 0.333 0 --> 1 \| \| 6 1 0.500 0 --> 1 \| \| 9 1 0.500 0 --> 1 \| \| 10 1 0.500 1 --> 0 \| \| 25 1 0.500 0 --> 1 \| \| 36 1 1.000 1 ==> 0 \| \| 45 1 0.667 0 ==> 1 \| \| 48 1 0.200 0 ==> 1 \| \| 49 1 0.250 0 ==> 1 \| \| 50 1 0.200 0 --> 1 \| \| 64 1 0.500 0 ==> 1 \| \| 70 1 0.800 0 --> 1 \| \| 76 1 0.250 0 ==> 1 \| \| 83 1 0.500 0 ==> 1 \| \| 84 1 0.167 0 --> 1 \| \| node 16 --> node 14 56 1 0.167 1 --> 0 \| \| 57 1 0.250 0 ==> 1 \| \| 69 1 0.600 0 ==> 1 \| \| 72 1 0.333 0 ==> 1 \| \| 75 1 0.286 0 ==> 1 \| \| 81 1 0.143 0 --> 1 \| \| node 14 --> *Micropithecus* *clarki* 5 1 0.333 1 --> 0 \| \| 8 1 0.500 0 ==> 1 \| \| 9 1 0.500 1 --> 0 \| \| 11 1 0.250 1 --> 0 \| \| 22 1 0.500 0 ==> 1 \| \| 25 1 0.500 1 --> 0 \| \| 43 1 0.429 1 ==> 0 \| \| 44 1 0.200 1 ==> 0 \| \| 46 1 0.500 1 ==> 0 \| \| 52 1 0.250 1 ==> 0 \| \| 62 1 0.400 1 ==> 2 \| \| 67 1 0.500 0 ==> 1 \| \| 90 1 0.333 1 ==> 0 \| \| node 14 --> node 13 1 1 0.500 0 ==> 1 \| \| 16 1 0.333 0 ==> 1 \| \| 24 1 1.000 1 ==> 2 \| \| 85 1 0.333 0 ==> 1 \| \| 93 1 1.000 0 ==> 1 \| \| node 13 --> *Dendropithecus* *macinnesi* 12 1 0.333 1 ==> 0 \| \| 29 1 0.143 0 --> 1 \| \| 38 1 0.200 1 ==> 0 \| \| 40 1 0.200 1 ==> 0 \| \| 48 1 0.200 1 ==> 0 \| \| 66 1 0.667 0 ==> 1 \| \| 70 1 0.800 1 --> D \| \| 81 1 0.143 1 --> 0 \| \| 91 1 0.200 1 ==> 0 \| \| node 13 --> *Simiolus* *enjiessi* 21 1 0.333 1 ==> 0 \| \| 39 1 0.167 1 ==> 0 \| \| 51 1 0.200 1 ==> 0 \| \| 69 1 0.600 1 ==> 2 \| \| 70 1 0.800 1 --> 0 \| \| 71 1 0.200 1 ==> 0 \| \| 76 1 0.250 1 ==> 2 \| \| 77 1 0.500 0 ==> 1 \| \| 79 1 0.250 1 ==> 0 \| \| 80 1 0.200 1 ==> 0 \| \| 84 1 0.167 1 --> 0 \| \| node 16 --> node 15 51 1 0.200 1 ==> 0 \| \| 53 1 0.667 1 --> 0 \| \| 58 1 0.333 0 ==> 2 \| \| 60 1 0.333 1 --> 0 \| \| 66 1 0.667 0 ==> 1 \| \| 70 1 0.800 1 --> 2 \| \| 71 1 0.200 1 ==> 0 \| \| 77 1 0.500 0 --> 1 \| \| node 15 --> *Ekembo* *heseloni* 6 1 0.500 1 --> 0 \| \| 13 1 0.667 0 ==> 2 \| \| 18 1 0.500 1 ==> 0 \| \| 20 1 0.250 0 ==> 1 \| \| 29 1 0.143 0 --> 1 \| \| 38 1 0.200 1 ==> 0 \| \| 40 1 0.200 1 ==> 0 \| \| 50 1 0.200 1 --> 0 \| \| 62 1 0.400 1 ==> 2 \| \| 63 1 0.500 0 ==> 1 \| \| 70 1 0.800 2 --> B \| \| 84 1 0.167 1 --> 0 \| \| node 15 --> *Victoriapithecus* *macinnesi* 1 1 0.500 0 ==> 1 \| \| 10 1 0.500 0 --> 1 \| \| 11 1 0.250 1 --> 0 \| \| 14 1 0.500 0 ==> 1 \| \| 15 1 0.250 1 ==> 0 \| \| 16 1 0.333 0 ==> 1 \| \| 17 1 0.500 0 --> 1 \| \| 19 1 0.250 1 ==> 0 \| \| 21 1 0.333 1 ==> 0 \| \| 22 1 0.500 0 ==> 1 \| \| 23 1 0.250 0 ==> 1 \| \| 28 1 1.000 1 ==> 0 \| \| 31 1 1.000 0 ==> 1 \| \| 32 1 0.333 0 ==> 1 \| \| 33 1 0.200 0 --> 1 \| \| 34 1 1.000 1 ==> 2 \| \| 37 1 0.333 0 ==> 1 \| \| 39 1 0.167 1 ==> 0 \| \| 42 1 1.000 0 ==> 1 \| \| 47 1 0.167 0 ==> 1 \| \| 52 1 0.250 1 ==> 0 \| \| 53 1 0.667 0 --> 2 \| \| 59 1 1.000 0 ==> 1 \| \| 64 1 0.500 1 ==> 2 \| \| 65 1 0.667 1 ==> 2 \| \| 69 1 0.600 0 --> A \| \| 76 1 0.250 1 ==> 2 \| \| 77 1 0.500 1 --> 2 \| \| 82 1 0.600 0 ==> D \| \| 87 1 0.333 0 ==> 1 \| \| 88 1 0.500 0 ==> 1 \| \| 90 1 0.333 1 ==> 0 \| |
| --- | --- | --- | --- | --- | --- | --- | --- | --- | --- | --- | --- | --- | --- | --- | --- | --- | --- | --- | --- | --- | --- | --- | --- | --- | --- | --- | --- | --- | --- | --- | --- | --- | --- | --- | --- | --- | --- | --- | --- | --- | --- | --- | --- | --- | --- | --- | --- | --- | --- | --- | --- | --- | --- | --- | --- | --- | --- | --- | --- | --- | --- | --- | --- | --- | --- | --- | --- | --- | --- | --- | --- | --- | --- | --- | --- | --- | --- | --- | --- | --- | --- | --- | --- | --- | --- | --- | --- | --- | --- | --- | --- | --- | --- | --- | --- | --- | --- | --- | --- | --- | --- | --- | --- | --- | --- | --- | --- | --- | --- | --- | --- | --- | --- | --- | --- | --- | --- | --- | --- | --- | --- | --- | --- | --- | --- | --- | --- | --- | --- | --- | --- | --- | --- | --- | --- | --- | --- | --- | --- | --- | --- | --- | --- | --- | --- | --- | --- | --- | --- | --- | --- | --- | --- | --- | --- | --- | --- | --- | --- | --- | --- | --- | --- | --- | --- | --- | --- | --- | --- | --- | --- | --- | --- | --- | --- | --- | --- | --- | --- | --- | --- | --- | --- | --- | --- | --- | --- | --- | --- | --- | --- | --- | --- | --- | --- | --- | --- | --- | --- | --- | --- | --- | --- | --- | --- | --- | --- | --- | --- | --- | --- | --- | --- | --- | --- | --- | --- | --- | --- | --- | --- | --- | --- | --- | --- | --- | --- | --- | --- | --- | --- | --- | --- | --- | --- | --- | --- | --- | --- | --- | --- | --- | --- | --- | --- | --- | --- | --- | --- | --- | --- | --- | --- | --- | --- | --- | --- | --- | --- | --- | --- | --- | --- | --- | --- | --- | --- | --- | --- | --- | --- | --- | --- | --- | --- | --- | --- | --- | --- | --- | --- | --- | --- | --- | --- | --- | --- | --- | --- | --- | --- | --- | --- | --- | --- | --- | --- | --- | --- | --- | --- | --- | --- | --- | --- | --- | --- | --- | --- | --- | --- | --- | --- | --- | --- | --- | --- | --- | --- | --- |
