# Supplementary material for: The Miocene primate Pliobates is a pliopithecoid
Source: Nat Commun. 2024 Apr 1;15:2822. doi: 10.1038/s41467-024-47034-9 (PMC10984959; doi:10.1038/s41467-024-47034-9)
Supplement: Supplementary file 7 — Supplementary Data 4 [file 41467_2024_47034_MOESM7_ESM.docx]

| **Dental** | |
| --- | --- |
| **#001** | I1–I2 size heteromorphy: 0, low; 1, moderate; 2, marked. |
| **#002** | I1–I2 lingual morphology: 0, lingual features (other than lingual cingulum, if present) absent or poorly-developed; 1, lingual features (basal bulge, pillar and/or crenulations) well developed. |
| **#003** | Lingual cingulum and crown base of I1: 0, narrow cingulum with slender crown base; 1, broad cingulum with bulging crown base. |
| **#004** | I2 morphology: 0, peg-shaped; 1, spatulate. |
| **#005** | Apicobasal height of I1 relative to mesiodistal length: 0, low-crowned; 1, high-crowned. |
| **#006** | Apicobasal height of I2 relative to mesiodistal length: 0, low-crowned; 1, high-crowned. |
| **#007** | i2 mesiodistal waisting: 0, absent; 1, present. |
| **#008** | i2 mesial margin: 0, straight to slightly inclined; 1, clearly inclined mesialward. |
| **#009** | i2 distal margin: 0, markedly angled; 1, rather straight. |
| **#010** | i2 distal prong: 0, absent to slightly developed; 1, well developed. |
| **#011** | Apicobasal height of i1 relative to mesiodistal length: 0, high-crowned; 1, very high-crowned. |
| **#012** | Apicobasal height of i2 relative to mesiodistal length: 0, high-crowned; 1, very high-crowned. |
| **#013** | Male C1 crown shape: 0, moderatelyv compressed but not very high and not markedly dagger-like; 1, male upper canine crown very compressed, high, and dagger-like; 2, male upper canine crown stouter and less compressed. |
| **#014** | Male C1 sulcus: 0, sulcus not extending onto root; 1, sulcus extending onto root. |
| **#015** | Female c1 length/breadth index: 0, broad; 1, narrow. |
| **#016** | Female c1 apicobasal height relative to mesiodistal length: 0, low-crowned; 1, high-crowned. |
| **#017** | Mesiolingual cristid-lingual cingulid thickening in the female c1: 0, restricted angle to rounded angle; 1, slightly prominent angle; 2, prominent angle, cuspulid-like. |
| **#018** | Position of the Mesiolingual cristid-lingual cingulid junction in the female c1: 0, low; 1, at about crown-midheight; 2, higher. |
| **#019** | P3 breadth/length index: 0, very broad; 1, broad. |
| **#020** | P3 length relative to P4 length: 0, P3 shorter to subequal in length to P4; 1, P3 longer than P4. |
| **#021** | P3 length relative to M2 length: 0, P3 markedly shorter than M2; 1, P3 moderately shorter than M2. |
| **#022** | P3 cusp heteromorphy: 0, strong with tall paracone; 1, reduced with tall paracone; 2, reduced with low paracone. |
| **#023** | P3 central fovea shape: 0, broader than long; 1, relatively narrower. |
| **#024** | P3 buccal wall shape: 0, clearly triangular, without mesiobuccal expansion onto root; 1, subtriangular or somewhat rhomboid, with clear mesiobuccal expansion onto root; 2, slightly rhomboid, with no mesiobuccal expansion onto root; 3, clearly rhomboid. |
| **#025** | P3 buccal wall height in buccal view: 0, low; 1, high. |
| **#026** | P3 lingual cingulum: 0, conspicuously present in most instances; 1, usually absent or poorly developed. |
| **#027** | P3 paracone position along the crown buccolingual axis: 0, very peripheral; 1, not very peripheral. |
| **#028** | P4 occlusal contour: 0, (sub)triangular; 1, (sub)oval to (sub)elliptical. |
| **#029** | P4 breadth/length index: 0, very broad; 1, broad. |
| **#030** | P4 length relative to M2 length: 0, P4 markedly shorter than M2; 1, P4 moderately shorter than M2. |
| **#031** | P4 lingual cingulum: 0, conspicuously present in most instances; 1, usually absent or poorly developed. |
| **#032** | P4 central fovea shape: 0, broader than long; 1, relatively narrower. |
| **#033** | P4 paracone position along the crown buccolingual axis: 0, very peripheral; 1, not very peripheral. |
| **#034** | p3 sectoriality: 0, non-sectorial to poorly sectorial; 1, moderately sectorial; 2, markedly to strongly sectorial. |
| **#035** | p3 mesiobuccal honing face length: 0, long; 1, medium-size; 2, short. |
| **#036** | p3 mesiobuccal honing face inclination: 0, inclined; 1, steep. |
| **#037** | Metaconid on p3: 0, absent; 1, small; 2, prominent. |
| **#038** | p4 crown shape: 0, (sub)rectangular to (sub)elliptical; 1, more clearly (sub)oval. |
| **#039** | p4 breadth/length index: 0, very to slightly narrow; 1, slightly broader than long. |
| **#040** | p4 length relative to m2 length: 0, p4 markedly shorter than m2; 1, p4 moderately shorter than m2. |
| **#041** | p4 buccal cingulid: 0, shelf-like from mesial to distal; 1, clearly disrupted or limited to some portions of the crown. |
| **#042** | Bilophodont molars: 0, absent; 1, present. |
| **#043** | Molar cusp morphology: 0, rounded cusps; 1, pyramidal cusps; 2, pyramidal but somewhat compressed cusps; 3, very compressed cusps. |
| **#044** | Upper molar cusp height: 0, low cusps; 1, high cusps. |
| **#045** | (Lower) molar cusp peripheralization: 0, cusps not peripheral; 1, cusps moderately peripheral; 2, cusps very peripheral. |
| **#046** | Molar crest morphology: 0, blunt crests; 1, moderately sharp crests; 2, markedly sharp crests. |
| **#047** | M1–M2 waisting: 0, slight to moderate; 1, marked. |
| **#048** | M1 length/breadth index: 0, very broad; 1, moderately broad. |
| **#049** | M2 length/breadth index: 0, very broad; 1, moderately broad. |
| **#050** | M3 length/breadth index: 0, very broad; 1, moderately broad. |
| **#051** | M1/M2 length ratio: 0, M1 moderately shorter than M2; 1, M1 slightly shorter to subequal in length to M2. |
| **#052** | M3/M2 length ratio: 0, M3 moderately shorter than M2; 1, M3 slightly shorter to subequal in length to M2. |
| **#053** | M1–M3 protoconule: 0, present and large; 1, present and small; 2, usually absent or poorly distinct. |
| **#054** | M1–M2 buccal cingulum: 0, well-developed; 1, variously present or discontinuous/poorly developed; 2, absent. |
| **#055** | M3 buccal cingulum: 0, well-developed; 1, variously present or discontinuous/poorly developed; 2, absent. |
| **#056** | M1–M2 lingual cingulum breadth: 0, well-developed; 1, reduced or discontinuous; 2, absent. |
| **#057** | M1–M2 lingual cingulum distal development: 0, not surrounding the hypocone; 1, surrounding the hypocone or showing clear distal style(s). |
| **#058** | M3 lingual cingulum: 0, well-developed; 1, reduced or discontinuous; 2, absent. |
| **#059** | Crista obliqua in upper molars: 0, present in all instances; 1, variably present; 2, absent. |
| **#060** | M2–M3 metacone size: 0, large; 1, small (compared to the protocone and paracone). |
| **#061** | M1–M2 hypocone size (relative to metacone and paracone): 0, similarly sized to slightly smaller; 1, much smaller. |
| **#062** | M3 hypocone size (relative to metacone and paracone): 0, large; 1, small; 2, absent or rudimentary. |
| **#063** | M1–M2 paracone buccal position relative to the metacone (more marked in the M2): 0, markedly buccal; 1, buccal. |
| **#064** | M1–M2 protocone distal position relative to the paracone: 0, clearly distal; 1, distal; 2, aligned. |
| **#065** | M3 protocone distal position relative to the paracone: 0, clearly distal; 1, distal; 2, aligned. |
| **#066** | M1–M2 hypocone lingual position relative to the protocone: 0, more distal than metacone and/or lingual than protocone; 1, in line with metacone and paracone. |
| **#067** | M3 hypocone lingual position relative to the protocone: 0, in line with metacone and paracone; 1, more distal than metacone and/or lingual than protocone. |
| **#068** | M1–M2 buccal profile length: 0, equal in size to moderately longer than the lingual profile; 1, markedly longer than the lingual profile. |
| **#069** | M1–M3 hypocone-metacone crista: 0, discontinuous or absent; 1, variably present but without constituting a true hypoloph; 2, true hypoloph. |
| **#070** | M1–M2 prehypocrista: 0, present and meets protocone; 1, variably present (meets protocone); 2, present and meets crista obliqua. |
| **#071** | M1–M2 trigon basin: 0, as long as broad; 1, broader than long. |
| **#072** | M1–M2 distal fovea: 0, shorter than trigon; 1, clearly longer than trigon, at least in some molars. |
| **#073** | m1–m3 cusps: 0, medium-sized to extensive; 1, more discrete. |
| **#074** | m1–m3 mesial arm of pliopithecine triangle: 0, always absent; 1, variably present and/or incipient; 2, present in some molar, constituting a pliopithecine triangle. |
| **#075** | m1–m3 distal arm of pliopithecine triangle: 0, always absent; 1, variably present and/or poorly constituted; 2, well constituted. |
| **#076** | m1–m3 buccal cingulid width: 0, Ledge-like; 1, moderately broad; 2, narrow to absent. |
| **#077** | m1–m3 buccal cingulid extent: 0, continuous at least in some molar; 1, usually discontinuous; 2, limited to some stylids. |
| **#078** | m1–m3 mesial fovea: 0, very large, about as long as broad; 1, small to restricted, clearly broader than long. |
| **#079** | m1 breadth/length index: 0, very narrow; 1, moderately narrow. |
| **#080** | m2 breadth/length index: 0, very narrow; 1, moderately narrow. |
| **#081** | m1/m2 length ratio: 0, m1 moderately shorter than m2; 1, m1 only slightly shorter than m2. |
| **#082** | m1–m2 hypoconulid size: 0, present and well developed in all instances; 1, present in all instances but clearly reduced in size; 2, almost indistinguishable or absent. |
| **#083** | m1–m2 protoconid mesial position relative to the metaconid: 0, clearly more mesial; 1, almost transversely aligned. |
| **#084** | m1–m2 entoconid distal position relative to the hypoconid: 0, clearly more distal; 1, almost transversely aligned. |
| **#085** | m1–m2 hypoconulid position if present: 0, median or slightly buccal; 1, clearly buccal. |
| **#086** | m1–m3 crest pattern: 0, hypoprotocristid originates lingually from protoconid, cristid obliqua (if present) joins the protoconid distally; 1, hypoprotocristid originates distally or distolingually from protoconid, from where the inclined cristid obliqua joins protoconid, at least in m1; 2, hypoprotocristid originates distally from protoconid, the inclined cristid obliqua joins the hypoprotocristid-hypometacristid (Y crest pattern) at least in m1; 3, only cristid obliquid is present so that the mesial fovea communicates with the talonid. |
| **#087** | m1–m3 postcristid-hypoentocristid: 0, present; 1, absent. |
| **#088** | m1–m3 talonid secondary wrinkling: 0, absent to weak; 1, moderate; 2, marked. |
| **#089** | m1–m2 distal fovea: 0, opens on the talonid basin (at least in some molars); 1, large to medium-sized and separated from the talonid basin; 2, small and separated from the talonid basin. |
| **#090** | m3 breadth/length index: 0, very narrow; 1, moderately narrow. |
| **#091** | m3/m2 length ratio: 0, m1 moderately shorter than m2; 1, m1 only slightly shorter than m2. |
| **#092** | p4 distal tubercles: 0, small or indistinct; 1, prominent. |
| **#093** | m3 entoconid relative size: 0, small; 1, large. |
| **#094** | m3 hypoconulid size: 0, present and well developed in all instances; 1, present in all instances but clearly reduced in size; 2, almost indistinguishable or absent. |
| **#095** | m3 hypoconulid position if present: 0, median or slightly buccal; 1, clearly buccal. |
| **#096** | Lower incisor lingual enamel: 0, present; 1, absent. |
| **#097** | m3 hypoconid and entoconid linked by crest: 0, no; 1, yes; 2, true distal lophid. |
| **Cranial** | |
| **#098** | Splanchnocranium orientation relative to cranial base: 0, slightly klinorhynch; 1, airorhynch; 2, strongly klinorhynch. |
| **#099** | Inion position relative to glabella (with the cranium oriented in the Frankfurt plane): 0, on the same plane; 1, inion below glabella. |
| **#100** | Rostrum length: 0, long rostrum, distance from orbitale to the anterior-most point of maxilla roughly equals C1–M1 tooth row length; 1, short rostrum, distance from orbitale to the anterior-most point of maxilla roughly equals P1–C1 tooth row length. |
| **#101** | Angle between interorbital plane/nasals and frontal squama in lateral view: 0, clearly obtuse angle (>90º); 1, roughly right angle (90º). |
| **#102** | Orbitale-rhinion distance relative to tooth row (with the cranium oriented in the Frankfurt plane): 0, long (C1–M2); 1, short (C1–P4); very short (M1–M2). |
| **#103** | Orbitale position relative to the tooth row (with the cranium oriented in the Frankfurt plane): 0, over the molars; 1, over the premolars. |
| **#104** | Rhinion position relative to the tooth row (with the cranium oriented in the Frankfurt plane): 0, over C1–P3; 1, over P4–M1. |
| **#105** | Glabella position relative to the tooth row (with the cranium oriented in the Frankfurt plane): 0, over the molars; 1, over the premolars/canine. |
| **#106** | Supraorbital region: 0, superciliary arches; 1, supraorbital costae, coalescent with temporal lines; 2, supraorbital torus with inflated glabella. |
| **#107** | Frontal sulcus: 0, absent; 1, depression of the mid-frontal region just above glabella; 1, well- developed frontal sulcus present. |
| **#108** | Orbital/nasal surface: 0, concave; 1, flat. |
| **#109** | Premaxillomaxillary suture: 0, contacts nasal near midpoint; 1, contacts nasal (or frontal) superiorly; 2, contacts nasal inferiorly. |
| **#110** | Interorbital pillar: 0, broad; 1, narrow. |
| **#111** | Orbital shape: 0, (sub)circular; 1, oval (higher than broad); 2, oval, rectangular or rhomboid (broader than high). |
| **#112** | Orbital convergence (relative to the sagittal plane): 1, slightly laterally-directed orbits; 2, frontalized orbits. |
| **#113** | Orbital frontation (relative to the Frankfurt plane): 0, orbits posteriorly inclined; 1, orbits close to vertical. |
| **#114** | anteriorly or slightly laterally; 1, thin, sharp and laterally-facing lateral orbital rim, with the the zygomaticomaxillary foramina facing laterally. |
| **#115** | Infraorbital plane: 0, curved; 1, flat. |
| **#116** | Position of the infraorbital foramen relative to the orbit: 0, low; 1, very close to the orbital rim. |
| **#117** | Position of the infraorbital foramen relative to tooth row (with the cranium oriented in the Frankfurt plane): 0, above molars; 1, above P3–P4; 2, anteriorly from the premolars. |
| **#118** | Zygomatic root pneumatization: 0, solid; 1, hollow. |
| **#119** | Zygomatic root height relative to the alveolar plane: 0, low (close to the alveolar plane); 1, moderately high; 2, very high. |
| **#120** | Zygomatic height: 0, high; 1, low. |
| **#121** | Zygomatic root position relative to the tooth row (with the cranium oriented in the Frankfurt plane): 0, over the M2; 1, over the M1. |
| **#122** | Zygomatic orientation in lateral view (relative to the alveolar plane): 0, vertical: 1, posteriorly inclined; 2, anteriorly inclined. |
| **#123** | Frontozygomatic suture: 0, vertical; 1, medially directed. |
| **#124** | Ethmosphenoid contact: 0, none or very restricted (0-39%); 1, moderately short contact (40-90%); long contact (91-100%). |
| **#125** | Pterygoid process: 0, robust; 1, compressed. |
| **#126** | Nasal bones at nasion: 0, broad; 1, narrow. |
| **#127** | Nasal bones length: 0, short; 1, moderately long; 2, very long. |
| **#128** | Nasal aperture position relative to the alveolar plane: 0, low; 1, high. |
| **#129** | Frontal sinus: 0, absent; 1, present. |
| **#130** | Maxillary sinus size: 0, absent; 1, small; 2, large; 3, very extensive. |
| **#131** | Maxillary sinus floor: 0, low; 1, high. |
| **#132** | Maxillary sinus position relative to the tooth row: 0, at the level of C1; 1, at the level of P3–P4; 2, behind the premolars. |
| **#133** | Atrioturbinal ridges: 0, present and well developed; 1, present but moderate to weak; 2, absent. |
| **#134** | Premaxillary suture: 0, does not contact nasals or contacts nasals below the superior-most level of nasal aperture; 1, contacts nasals near their mid-height and above the superior-most level of nasal aperture; 2, contacts nasals near their superior-most level. |
| **#135** | Alveolar prognathism of the premaxilla: 0, absent; 1, present. |
| **#136** | Premaxilla ascending wing: 0, broad; 1, narrow. |
| **#137** | Subnasal floor: 0, fenestrated; 1, stepped; 2, smooth. |
| **#138** | Incisive fossa position: 0, opposite to C1; 1, distal to C1; 2, distal to P3. |
| **#139** | Incisive canal caliber: 0, absent; 1, large; 2, small. |
| **#140** | Greater palatine foramen: 0, round; 1, elongate. |
| **#141** | Number of incisive foramina: 0, double (i.e., one on each side of the midline); 1, single (confluence of two foramina, at least close to the surface). |
| **#142** | Size of incisive foramina: 0, large; 1, small; 2, tiny. |
| **#143** | Palate: 0, shallow; 1, deep. |
| **#144** | Posterior palate: 0, shallow; 1, deep. |
| **#145** | Canine fossa: 0, shallow; 1, deep. |
| **#146** | Palate shape: 0, anteriorly narrower; 1, parallel tooth rows; 2, bowed laterally. |
| **#147** | Palatine process: 0, thin; 1, thick. |
| **#148** | Palatine crest: 0, strong; 1, weak. |
| **#149** | Glenoid fossa: 0, shallow; 1, deep. |
| **#150** | Entoglenoid process: 0, large and pronounced; 1, diminutive, indistinct or absent; 2, moderately developed. |
| **#151** | Articular portion (postglenoid process) and tympanic portion of temporal bone (ordered): 0, unfused; 1, fused. |
| **#152** | Postglenoid foramen (ordered): 0, present; 1, absent. |
| **#153** | Temporal fossa: 0, narrow; 1, broader. |
| **#154** | Length of the frontal in temporal fossa: 0, short; 1, long. |
| **#155** | Condylar canal: 0, absent; 1, present. |
| **#156** | Ectotympanic: 0, annular; 1, tubular. |
| **#157** | Ossification of the external auditory meatus (ordered): 0, not ossified (annular ectotympanic); 1, large portion of the meatus not ossified (semitubular ectotympanic); 2, almost completely ossified (only the lateral end is not ossified); 3, tubular ectotympanic, completely ossified. |
| **#158** | Length of the tubular ectotympanic (ordered): 0, short external auditory tube reaching only the postglenoid process; 1, auditory tube long surpassing the postglenoid process. |
| **#159** | Depth of the subarcuate fossa: 0, deep; 1, moderately deep to shallow; 2, very shallow to non- existent. |
| **#160** | Anteroposterior position of the carotid foramen on the bulla: 0, posterior (close to the occipital and far from the Eustachian aperture; posterior to line joining midpoints of tympanic bones); 1, more anterior (over the line joining midpoints of tympanic bones). |
| **#161** | Foramen ovale position : 0, posteriorly situated, just below the Eustachian aperture, between the glenoid eminence and the bulla; 1, placed well anterior to the Eustachian aperture and laterally displaced. |
| **#162** | Bulla tympanica inflation: 0, well inflated; 1, little inflated; 2, not inflated at all. |
| **#163** | Foramen spinosum (ordered): 0, present; 1, absent. |
| **#164** | Jugular foramen: 0, small and lateralized; 1, large and occlusally visible. |
| **#165** | Mandibular condyle height: 0, lower than coronoid; 1, equal or higher than coronoid. |
| **#166** | Most lateral position of the occipital crest relative to the anterior-most point of the occipital-temporal suture (in basioccipital view): 0, posterior; 1, anterior. |
| **#167** | Carotid canal orientation: 0, oblique; 1, vertical. |
| **#168** | Carotid canal direction: 0, anterior; 1, medial. |
| **#169** | Size of the vestibule relative to the semicircular canals: 0, small; 1, large. |
| **#170** | Robusticity of the semicircular canals: 0, slender; 1, stout. |
| **#171** | Shape of the anterior semicircular canal: 0, vertically compressed; 1, rounded; 2, elongated superiorly. |
| **#172** | Shape of the anterior portion of the anterior semicircular canal: 0, non-projecting anterosuperiorly; 1, anterosuperiorly projecting. |
| **#173** | Shape of the posterior semicircular canal: 0, vertically compressed; 1, rounded; 2, elongated superiorly. |
| **#174** | Shape of the lateral semicircular canal ampullary portion: 0, flat or only slightly bent superiorly; 1, markedly bent superiorly. |
| **#175** | Length of the common crus: 0, long; 1, intermediate; 2, short. |
| **Postcranial** | |
| **#176** | Humeral head torsion 0, <15º; 1, >15º. |
| **#177** | Humeral head shape 0, oblong, extending between tuberosities; 1, less oblong; 2, spherical with tuberosities anterior. |
| **#178** | Humeral head height 0, lower than tuberosities; 1, as high or slightly higher than tuberosities; 2, much higher than tuberosities. |
| **#179** | Humeral tuberosities relative sizes 0, lesser tuberosity <60% of greater tuberosity; 1, lesser tuberosity 60-80% of greater tuberosity. |
| **#180** | Humeral intertuberosity angle 0, <90º; 1, >90º. |
| **#181** | Bicipital groove in humerus 0, broad; 1, intermediate; 2, narrow. |
| **#182** | Deltoid insertion in humerus 0, on proximal half of shaft; 1, on distal half. |
| **#183** | Deltopectoral plane in humerus 0, prominent; 1, reduced. |
| **#184** | Proximal humeral shaft shape 0, angular; 1, rounded. |
| **#185** | Humeral shaft curvature 0, retroflected; 1, straight; 2, strongly retroflected. |
| **#186** | Length of the humerus relative to body mass 0, short; 1, long. |
| **#187** | Entepicondylar foramen in humerus (ordered) 0, present; 1, absent. |
| **#188** | Olecranon fossa in humerus 0, shallow; 1, deep, sharp laterally. |
| **#189** | Olecranon fossa articular surface in humerus 0, no extension into fossa; 1, extends into fossa laterally. |
| **#190** | Coronoid/radial fossae size in humerus 0, radial > coronoid; 1, radial < coronoid. |
| **#191** | Coronoid fossa in humerus 0, shallow; 1, deep. |
| **#192** | Medial epicondyle/keel in humerus 0, distinct; 1, merged. |
| **#193** | Medial epicondyle orientation in humerus 0, very retroflexed; 1, slightly retroflexed; 2, medial. |
| **#194** | Humeral capitulum proportions 0, longer proximodistally; 1, similar proximodistal and anteroposterior dimensions. |
| **#195** | Humeral capitulum articular surface 0, no distal expansion of articular surface; 1, distal expansion of articular surface. |
| **#196** | Humeral trochlear shape 0, cylindrical with a weak medial keel; 1, trochleiform with a strong medial keel. |
| **#197** | Humeral trochlear breadth 0, < capitular breadth 1, > capitular breadth. |
| **#198** | Humeral trochlear proximal border 0, straight; 1, notched (V-shaped for coronoid beak). |
| **#199** | Humeral conoid facet 0, absent; 1, present. |
| **#200** | Lateral trochlear keel in humerus 0, weak; 1, moderate; 2, strong. |
| **#201** | Medial trochlear keel in humerus 0, anterior to epicondyle; 1, projects distally from epicondyle. |
| **#202** | Zona conoidea width in humerus 0, broad; 1, narrow. |
| **#203** | Zona conoidea depth in humerus 0, shallow; 1, deep. |
| **#204** | Distal radioulnar articulation on radius 0, proximodistally narrow; 1, proximodistally expanded. |
| **#205** | Radial head outline 0, oval with flat posterolateral area; 1, smaller flat area; 2, round. |
| **#206** | Radial head lateral lip 0, large; 1, present; 2, absent. |
| **#207** | Radial neck shape 0, oval; 1, circular. |
| **#208** | Humeral capitular tail 0, present; 1, absent. |
| **#209** | Sigmoid median keel in ulna 0, none; 1, strong keel. |
| **#210** | Humeral shaft 0, shaft concave in anterior view; 1, shaft straight. |
| **#211** | Radius length relative to body mass 0, short; 1, long 2, very long. |
| **#212** | Radial head fossa for capitulum 0, small; 1, relativelly large. |
| **#213** | Beveled surface in radius for humeral zona conoidea 0, absent or reduced; 1, present but not very developped; 2, extensive around the margin of radial head; 3, present all around the radial head. |
| **#214** | Radial lunate articular surface 0, anteroposteriorly narrow; 1, ventrally expanded. |
| **#215** | Ulnar olecranon process 0, not abbreviated; 1, abbreviated. |
| **#216** | Radial articulation with radial notch of the ulna 0, small; 1, extends along a great portion of the radial head; 2, extends all over the radial head. |
| **#217** | Radial shaft 0, small curvature; 1, very curved. |
| **#218** | Bicipital tuberosity in radius 0, facing anteriorly; 1, facing more laterally. |
| **#219** | Radial head tilt 0, marked; 1, low or absent. |
| **#220** | Ulnar shelf of radius 0, not excludes ulnar head from distal radiocarpal joint; 1, excludes ulnar head from joint: |
| **#221** | Radial notch shape in ulna 0, single oval facet; 1, two facets forming a right angle; 2, two distinct facets. |
| **#222** | Ulnar shaft cross-section 0, proximally flat; 1, proximally wider. |
| **#223** | Ulnar shaft bowing 0, convex dorsally; 1, concave or straight dorsally. |
| **#224** | Arm angle of ulna 0, inexistent or small; 1, large. |
| **#225** | Cubital angle in ulna 0, inexistent or low (<5º); 1, higher (>5º). |
| **#226** | Brachialis insertion in ulna 0, not buttressed medially; 1, buttressed medially. |
| **#227** | Laterally-facing ulnar articular area with lateral side of humeral trochlea 0, reduced; 1, extensive. |
| **#228** | Radial notch in ulna 0, anterolaterally oriented; 1, laterally oriented. |
| **#229** | Trochlear notch in ulna 0, mediolaterally narrow; 1, mediolaterally wide. |
| **#230** | Olecranon beak in ulna 0, relatively long; 1, relatively short. |
| **#231** | Radioulnar joint 0, non diarthrodial; 1, fully diarthrodial. |
| **#232** | Ulnar styloid process 0, massive; 1, with reduced girth. |
| **#233** | Ulnar styloid process length 0, long; 1, short. |
| **#234** | Ulnar styloid process articulation 0, articulates with pisiform; 1, does not articulate with pisiform. |
| **#235** | Ulnar head 0, small facet; 1, large semilunar articulation. |
| **#236** | Ulnar styloid process 0, distally straight or ulnarly horiented; 1, hook-like, radially facing. |
| **#237** | Ulnar head 0, widest transverse area placed close to styloid; 1, widest transverse area placed on radial articulation. |
| **#238** | Ulnar fovea (ordered) 0, not developed; 1, partially developed; 2, fully developed. |
| **#239** | Dorsal epitrochlear fossa 0, deep and large; 1, restricted and shallow; 2, absent. |
| **#240** | Position of the groove for the extensor pollicis longus tendon 0, dorsomedial; 1, dorsal over lunate facet. |
| **#241** | Groove for the m. extensor carpi ulnaris tendon 0, strong and laterally limited by a dorsally prominent ridge; 1, lateral ridge smooth or inexistent; 2, canal absent. |
| **#242** | Ulnar styloid articulation with triquetrum 0, present; 1, absent. |
| **#243** | Dorsal ridges on distal radius and scaphoid 0, absent; 1, present. |
| **#244** | Os centrale facet on scaphoid 0, not beaked; 1, beaked. |
| **#245** | Os centrale and scaphoid 0, separate; 1, fused. |
| **#246** | Os centrale articulation with trapezium 0, present; 1, absent. |
| **#247** | Scaphoid-radial facet in lunate 0, low angle; 1, high angle. |
| **#248** | Lunate mediolateral width 0, narrow; 1, broad. |
| **#249** | Trapezium-metacarpal I joint 0, sellar; 1, modified hinge; 2, non-sellar. |
| **#250** | Capitate head mediolaterally 0, broad; 1, narrow. |
| **#251** | Dorsal/palmar extension of the proximal articular facet of the capitate 0, < 1.4; 1, ≥ 1.4. |
| **#252** | Proximal capitate surface 0, hemispherical in lateral view; 1, oblong in lateral view. |
| **#253** | Hamate border of the capitate 0, ulnarly oriented; 1, radially oriented. |
| **#254** | Centrale facet on capitate 0, flat; 1, concavoconvex. |
| **#255** | Metacarpal II facet on capitate 0, continuous; 1, divided. |
| **#256** | Metacarpal III facet on capitate 0, flat; 1, irregular. |
| **#257** | Hamate hamulus 0, not projecting; 1, distally projecting. |
| **#258** | Hamate distolateral edge 0, uninterrupted; 1, interrupted by ligament pit. |
| **#259** | Metacarpal IV facet on hamate 0, deep; 1, shallow. |
| **#260** | Dorsal extension of metacarpal head articular surfaces 0, absent; 1, present. |
| **#261** | Dorsal transverse ridge on metacarpal heads 0, absent; 1, present. |
| **#262** | Metacarpal I proximal articulation 0, no lateral extension; 1, lateral extension. |
| **#263** | Metarcapal I head dorsal part 0, narrow; 1, browed. |
| **#264** | Metacarpal II medial facet 0, undivided; 1, divided by ligament pit. |
| **#265** | Distal metacarpal shape 0, widest palmarly; 1, quadrilateral. |
| **#266** | Proximal articulation surface of proximal phalanges 0, oval; 1, square. |
| **#267** | Proximal phalangeal palmar tubercles 0, large; 1, small. |
| **#268** | Phalangeal secondary shaft features 0, small; 1, large. |
| **#269** | Palmar gutters in metacarpals head 0, strong; 1, poorly-developed. |
| **#270** | Pollical distal phalanx’s tendon insertion in palmar side 0, present; 1, poorly-developed or absent. |
| **#271** | Scaphoid tubercle length 0, similarly long or longer than body; 1, shorter. |
| **#272** | Triquetral surface on hamate is highly globular proximally 0, absent; 1, present. |
| **#273** | Triquetral surface on hamate is proximodistally long and narrow 0, absent; 1, present. |
| **#274** | Large pits for collateral ligaments on metacarpals converging dorsally 0, absent; 1, present. |
| **#275** | Proximal articular surface on proximal phalanges is dorsally canted 0, present; 1, absent. |
| **#276** | Trochlea in proximal phalanges is palmarly bent or “beaked” 0, absent; 1, present. |
| **#277** | Triquetrum size relative to hamate 0, large; 1, small. |
| **#278** | Lunate proximodistal length 0, long; 1, short. |
| **#279** | Trochleae in proximal phalanges are distally convergent (in palmar view) 0, present; 1, absent. |
| **#280** | Capitate and hamate proximally (heads) 0, capitate dominates; 1, hamate dominates. |
| **#281** | Reciprocal articular surfaces on capitate and metacarpal IV 0, present; 1, absent. |
| **#282** | Dorsal extension in proximal articular surface of metacarpal V 0, present; 1, absent. |
| **#283** | Pisiform position 0, not distally migrated; 1, distally migrated. |
| **#284** | Pisiform length 0, long; 1, short. |
| **#285** | Pisiform facet for ulnar styloid process 0, present; 1, absent. |
| **#286** | Pisiform orientation 0, palmar; 1, distal. |
| **#287** | Stylotriquetral facet relative size and presence 0, occupies mostly of the proximal surface; 1, occupies only half of the proximal surface; 2, smaller; 3, absent. |
| **#288** | Proximally protruding beak on the dorsoulnar side of the triquetrum 0, present; 1, absent. |
| **#289** | Triquetrum articular surface for hamate 0, relativelly small and flat; 1, large and concavo-convex. |
| **#290** | Triquetrum proximal and distal articular facets 0, paralell; 1, convergent. |
| **#291** | Articular surface for os centrale on the capitate 0, anteriorly oriented; 1, laterally oriented. |
| **#292** | Exposed metacarpal III facet on the lateral aspect of the capitate 0, not exposed; 1, well exposed. |
| **#293** | Size of trapezium facet on capitate 0, small; 1, large. |
| **#294** | Articular facet of the scafoid for lunate 0, narrow and small; 1, wide and large. |
| **#295** | Scapula shape 0, vertebral border smaller than caudal; 1, longer vertebral border. |
| **#296** | Scapula position relative to thorax 0, lateral; 1, dorsal. |
| **#297** | Scapular spine 0, oblique to axial border; 1, low angle relative to axial border. |
| **#298** | Spinous process root in scapula 0, robust; 1, gracile. |
| **#299** | Spinous process inclination in scapula 0, cranially inclined; 1, caudally inclined. |
| **#300** | Glenoid shape in scapula 0, elongate; 1, round. |
| **#301** | Glenoid curvature in scapula 0, more curved craniocaudally than dorsoventrally; 1, uniformly and moderately curved. |
| **#302** | Glenoid-axillary angle in scapula 0, large; 1, small. |
| **#303** | Infraglenoid tubercle in scapula 0, broad; 1, narrow. |
| **#304** | Teres minor attachment in scapula 0, axillary; 1, anterior. |
| **#305** | Coracoid deflection in scapula 0, less; 1, greatest. |
| **#306** | Acromial process in scapula 0, short; 1, elongate. |
| **#307** | Acromion root in scapula 0, inferior; 1, superior. |
| **#308** | Clavicle 0, short; 1, long. |
| **#309** | Torso shape 0, craniocaudally long and mediolaterally narrow; 1, craniocaudally short and mediolaterally broad. |
| **#310** | Sternum 0, long and narrow; 1, short and broad. |
| **#311** | Costal angle 0, low; 1, high. |
| **#312** | Ventral keeling of vertebral body 0, present; 1, absent or reduced. |
| **#313** | Vertebral body height 0, tall; 1, medium; 2, short. |
| **#314** | Spooling of lumbar centrum 0, present; 1, absent or reduced. |
| **#315** | Hollowing of lumbar centrum 0, present; 1, absent or reduced. |
| **#316** | Sternebrae 0, narrow; 1, intermediate; 2, broad. |
| **#317** | Accessory processes in vertebrae 0, large; 1, small; 2, absent. |
| **#318** | Transverse process position in lumbar vertebrae 0, ventral (on body); 1, intermediate (on body- pedicle junction); 2, dorsal (on pedicle). |
| **#319** | Transverse process inclination in lumbar vertebrae 0, ventrally inclined; 1, neutral (horizontally incline); 1, dorsally and caudally inclined. |
| **#320** | Modal number of lumbar vertebrae 0, ≥ 6; 1, ca. 5; 2, ≤ 4: |
| **#321** | Sacrum shape 0, broad; 1, narrow. |
| **#322** | Sacral number 0, 3 vertebrae; 1, 4 vertebrae; 2, 5 vertebrae; 3, 6 vertebrae. |
| **#323** | External tail 0, present; 1, absent. |
| **#324** | Iliac blade breadth 0, narrow; 1, intermediate; 2, wide. |
| **#325** | Lower iliac height 0, short; 1, long. |
| **#326** | Anterior inferior iliac spine 0, not knob-like; 1, knob-like. |
| **#327** | Anterior iliac spine 0, absent or separated; 1, near acetabulum. |
| **#328** | Acetabulum 0, expanded with raised lip; 1, cranially expanded lunate surface; 2, symmetrical lunate surface. |
| **#329** | Pubic length 0, short; 1, medium; 2, long. |
| **#330** | Ischial spine 0, distal to acetabulum; 1, same level as acetabulum. |
| **#331** | Femoral head height 0, below trochanter; 1, at or above trochanter. |
| **#332** | Size of femoral head relative to midshaft diameter 0, small; 1, expanded. |
| **#333** | Trochanteric fossa 0, open; 1, intermediate; 2, deep. |
| **#334** | Linea aspera ridge up to or above lesser trochanter 0, absent; 1, present. |
| **#335** | Femoral neck tubercle 0, present; 1, absent. |
| **#336** | Femoral shaft 0, vertical; 1, outwardly angled. |
| **#337** | Distal femur shaft shape 0, round; 1, anteroposteriorly flattened. |
| **#338** | Buttressing of intercondylar notch in femur 0, no buttressing; 1, buttressing. |
| **#339** | Patellar groove 0, narrow and deep; 1, broad and shallow; 2, wider than shaft. |
| **#340** | Femoral condyles 0, nearly symmetrical; 1, medial condyle larger. |
| **#341** | Femoral condyle depth 0, deep; 1, shallow. |
| **#342** | Popliteal groove in femur 0, shallow; 1, deep. |
| **#343** | Tibial condylar facets 0, symmetrical; 1, medial facet larger; 2, lateral facet larger. |
| **#344** | Lateral tibial facet 0, convex; 1, concave. |
| **#345** | Lateral proximal tibial facet 0, below the medial facet; 1, same level as medial facet. |
| **#346** | Distal tibial facet 0, square; 1, short. |
| **#347** | Lateral malleolus in tibia 0, small; 1, intermediate; 2, large. |
| **#348** | Fibular robusticity 0, thin; 1, robust. |
| **#349** | Distal fibula 0, robust and laterally flaring; 1, reduced, with less lateral flare. |
| **#350** | Talar trochlear depth 0, deep; 1, intermediate; 2, shallow. |
| **#351** | Talar trochlear wedging 0, strong; 1, weak. |
| **#352** | Talar trochlear rim asymmetry 0, subequal height; 1, lateral keel taller; 2, lateral keel much taller. |
| **#353** | Talar head 0, as wide as high; 1, wider than high. |
| **#354** | Fibular facet of talus 0, vertical; 1, laterally projecting. |
| **#355** | Curve of medial trochlear margin in talus 0, gently curved; 1, strongly curved. |
| **#356** | Anterior talar facet 0, curved; 1, intermediate; 2, flat. |
| **#357** | Talus height 0, tall; 1, short. |
| **#358** | Cotylar fossa in talus 0, distinct cup; 1, shallow. |
| **#359** | Plantar calcaneal tubercle 0, small; 1, large. |
| **#360** | Posterior calcaneoastragalar joint 0, faces medially; 1, faces dorsally. |
| **#361** | Calcaneocuboid joint 0, round facet with deep pit; 1, smaller pit. |
| **#362** | Sustentacular facet 0, undivided; 1, divided. |
| **#363** | Ectal facet curve in calcaneus 0, gently concave; 1, sharply concave. |
| **#364** | Ectal facet shape in calcaneus 0, broadest anterolaterally; 1, broadest proximomedially: |
| **#365** | Cuboid peg 0, small; 1, intermediate; 2, large. |
| **#366** | Cuboid wedging 0, slight; 1, stronger. |
| **#367** | Cuboid length 0, long; 1, short. |
| **#368** | Flexor hallucis longus groove 0, small; 1, intermediate; 2, large. |
| **#369** | Entocuneiform-metatarsal I joint 0, distal; 1, medial. |
| **#370** | Entocuneiform-metatarsal I facet 0, convex with medial extension; 1, less convex with slight extension. |
| **#371** | Cuneiform length 0, long; 1, short. |
| **#372** | Metartarsal I robusticity 0, gracile; 1, intermediate; 2, robust. |
| **#373** | Metartarsal I sesamoid grooves 0, small; 1, large. |
| **#374** | Metartarsal I head shape 0, symmetrical; 1, asymmetrical. |
| **#375** | Metartarsal I head position 0, aligned; 1, twisted. |
| **#376** | Metatarsal II lateral facet 0, divided; 1, single. |
| **#377** | Metatarsals II-V robusticity 0, gracile; 1, robust. |
| **#378** | Foot axis 0, runs through digit III; 1, runs through digit II. |
| **#379** | Phalangeal robusticity 0, gracile; 1, robust. |
| **#380** | Phalangeal curvature 0, straight; 1, curved. |
| **#381** | Phalangeal flexor ridges 0, weak; 1, intermediate; 2, strong. |
