# Supplementary material for: The Miocene primate Pliobates is a pliopithecoid
Source: Nat Commun. 2024 Apr 1;15:2822. doi: 10.1038/s41467-024-47034-9 (PMC10984959; doi:10.1038/s41467-024-47034-9)
Supplement: Supplementary file 8 — Supplementary Data 5 [file 41467_2024_47034_MOESM8_ESM.docx]

0*0*00000000000000000000000*0*0*0*0*00*0000000000*0*00*0*0000000000000*0*0000000000000*0*000000000000000000000*0*000011111111111111111111111

0*0*00000001111111111222222*2*2*2*2*33*3333333344*4*44*4*4444555555555*5*6666666666777*7*777777888888888899999*9*999900000000001111111112222

Taxon/character 1*2*34567890123456789012345*6*7*8*9*01*2345678901*2*34*5*6789012345678*9*0123456789012*3*456789012345678901234*5*678901234567890123456789012

*Aegyptopithecus* 00?0??0001000000?2011010?01111011111011000100000001110001000000000100000000001111000000021100000000000000010?0100000000000

*Saadanius* ???1?????????0?????10?1??????0?1?????????01??000000110000000000000100?10???????????????????????????000000000?01000000?0001

*Pliobates* 0?0???1101????0?21111012011101010????0??1021020110101110000112100011000000200000100101000??0?????1011110100000011101101102

*Micropithecus* 0000010100010010021011020111010001000011001010011110111010010201101011110011011110110001101000100??1??10?0???0?0???110010?

*Dendropithecus* 100?110010?00011??101003111111000100001100111100111111101001010110101D110011011100111000110010100??1?1?????????0???1?00?0?

*Simiolus* 10????0010110011021000031111010001000001001111011101111010010101101010010011110010101000111010100??1?1?????????0???1?00?0?

*Pliopithecus* 0000??10011?00101110100201111100A121001100110100001111100001A1100010001002D0011100000A00110001101??0??0????????0?0????0100

*Epipliopithecus* ?0000010011100????111002011111001121101100111100001101101001A1100010001101100110000010001010011011?10110100?00001001110100

*Dionysopithecus* ?00?0?1001?0???????????????1010001211?01?01001010111111010010110001010010220011000010100110001100?????????????????????????

*Barberapithecus* ?00?????????001111???????????????12110011021010000111110000111100011000002210010100100001010011?1???????????????0?????????

*Anapithecus* 0010??0101??0010210010120101010100212011103102011?1?111000000110001100000210101010000200201101100????????00???0??1???11?1?

*Plesiopliopithecus* ??????1101???????????????????????121000?002?02??????????????????????????0210000??10001002??0???0??????????????????????????

*Crouzelia* ?????????????????????????????????????001002?02??????????????????????????021100001D0001100??0??????????????????????????????

*Ekembo* 000010001011201000111002111111000110011000011101100101100000021110100B00000111110010000111100010000000000000200000?0000001

*Victoriapithecus* 100011001101010112000111111001111200100101111111110021100010010221001200000221110B11001010100010201000000110?1100000000002

*Macaca* 100011000100111112000111111000111200000121111111110122210120001221002000000220110211-0101010001122100000001011001000001001

*Pierolapithecus* ?11?????????20????101202111111100????????000?001101001110101011110101010???????????????????????????0000101012011101?011011

*Hispanopithecus* 11101?0010??20????1012031111011001??011110001000111102210101010110101010000221110010100111000010000011010100?011101?012000

*Hylobates* 00100001000010011001101201110111122001012000200111100221010001111010000000122111101000002100110001011110100000011101101102

*Pongo* 21100000100020010000120301110110011021112000100111110222-20101111010001000022111101010A2A100001001000201010121111010012000

*Gorilla* 11110000100020000010020200110100011021101011210111110221010101111010001000011111001010011101101002001000122020211000012000

*Pan* 11110000100020010001120201110110011021112000200111101221010101111010001000022111101010A0A100111002001101122020211000012000

11111111111111111111111111111111111111111111111111111111111111111111111111111222222222222222222222222222222222222222222222

222222*2*3*3*3333333344*4*44*4*4444555555555*5*6666666666777*7*777777888888888899999*9*9999000000000011111111122222222222333333333344444

Taxon/character 34567890123456789012345*6*7*8*9*01*2345678901*2*34*5*6789012345678*9*0123456789012*3*456789012345678901234*5*67890123456789012345678901234

*Aegyptopithecus* 0?00200201000000000000000?000000?00-?0?0?000??002020000110000?01000000100000000000???000??????????000???0000????????0??0??

*Saadanius* ???00001120010000?000100??000????120??????????0020200?????????????????????????????????????????????????????????????????????

*Pliobates* 0?0?10120110010001?001000?1011??0110111111?10000102010????1000011000001100001001111101011121?11110000110000?11011111101000

*Micropithecus* ???????201?1?0?0011000?1??????????????0???????????????????????????????????????????????????????????????????????????????0???

*Dendropithecus* ???????201?1?1?0000001?0?????????????????????????????????000001?100000100000000000000001?0000000000000000000000000000?????

*Simiolus* ???????201?1?1?0000001?0?????????????????????????????????000001?100000100000000000000001?0000000000000000000000000000?????

*Pliopithecus* ??????????????????????????????????????0???????????????01110??0??10?00?????????????????????????????????????????????????????

*Epipliopithecus* ?????012?????1??????0?10????0????11001?1??0?010010200011000011100000001000000000000000010000000000000000000000000000200?00

*Dionysopithecus* ??????????????????????????????????????0???????????????????????????????????????????????????????????????????????????????????

*Barberapithecus* ??????????????????????????????????????0???????????????????????????????????????????000????00????01?????????????????????????

*Anapithecus* ?????01201????????????00??????????????0???????????????????????????????????????????????????????????????????????????????????

*Plesiopliopithecus* ??????????????????????????????????????????????????????????????????????????????????????????????????????????????????????????

*Crouzelia* ??????????????????????????????????????0???????????????????????????????????????????????????????????????????????????????????

*Ekembo* ??0000110?01010000??000000000?00?120?0????0?01100011201??00000001110001110101101101101000111010010001001000010001011200000

*Victoriapithecus* 1?012000--221100010000020001000001200??0??001000101010001000?0201100010000000010000???0?????0?????20????0000????????000???

*Macaca* 10002000--2111000000110200010000013100001000100010101000101000201000010000000010000000000000000000200000000000000000200001

*Pierolapithecus* 1??11103121011?1????1101???????????????????????????????????????????????????????????????????????????????????????????????100

*Hispanopithecus* 0??1110201?00?11????1?11??100??0?1212?????1???0010101??????1111?11?110?101?11??11??????????????1?????????111????????2???00

*Hylobates* 00001001011001000100010100001100013111111111000001112021012011111111102111011201112211112131121111010110111111011112211000

*Pongo* 12011103121011212112111110000100013120021110001101A12121012111111111102111111201012211111131121111010111111110111012212100

*Gorilla* 01111113101011121011111111101111113120120110001100A1B12101211111111110211111120111121111013112111001010111111011101221211-

*Pan* 01110113101011121011111111101111113120120110001100B1B12101211111111110211111120111121111013112111001010111111011101221211-

22222222222222222222222222222222222222222222222222222223333333333333333333333333333333333333333333333333333333333333333333

444*4*4555555555*5*6666666666777*7*777777888888888899999*9*99990000000000111111111222222222223333333333444444444455555555556666666

Taxon/character 567890123456789012345*6*7*8*9*01*2345678901*2*34*5*6789012345678*9*0123456789012*3*456789012345678901234*5*6789012345678901234567890123456

*Aegyptopithecus* ????????????????0??????1??????10??1????????????????0??????????????????????????0???????1?1??10?210????????1011111000?0000??

*Saadanius* ??????????????????????????????????????????????????????????????????????????????????????????????????????????????????????????

*Pliobates* 0????111001110110???01101?0111111?0100111010111101???????????????????????????????????????????????????12?0101?10111010???10

*Micropithecus* ?????????????????????????????????????????????????????????????????????????????????????????1??1?????????????????????????????

*Dendropithecus* ???????????????????????????????????????????????????????????????????????????????????0??1?1????????????????111111110100000??

*Simiolus* ???????????????????????????????????????????????????????????????????????????????????0??1?1????????????????111111110100000??

*Pliopithecus* ???????????????10???0?????????????????????????????????????????????????????????????????????????????????????????????????????

*Epipliopithecus* ??101???????0001000000101?000111?00?0?000????????0???0000??0???100001000210010000?????11200011011121111??210110111111100??

*Dionysopithecus* ??????????????????????????????????????????????????????????????????????????????????????????????????????????????????????????

*Barberapithecus* ??????????????????????????????????????????????????????????????????????????????????????????????????????????????????????????

*Anapithecus* ???????????????????????????????????????????????????????????????????????????????????????????1??????????????????????????????

*Plesiopliopithecus* ??????????????????????????????????????????????????????????????????????????????????????????????????????????????????????????

*Crouzelia* ??????????????????????????????????????????????????????????????????????????????????????????????????????????????????????????

*Ekembo* 00100000000000010101000110000100100100000000000001?0010??000????0?0010001000??10010200110?0000?110???01101111100?00001??20

*Victoriapithecus* ????0??????????100??00000????100??0????????????????0??????????????????????????0?0??2?0002???1??????0101?11?100010?00?11???

*Macaca* 01100000000000010000000000100100010000000000000000000000001000100000200020000000000200002101100001010010110200010101111110

*Pierolapithecus* 00000100001101010??100011010010011000???1?21110100?1???????????11?11211??12?????1?????????????????????????????????????????

*Hispanopithecus* 0?100110001101010??101011??01101?00000?????????????11????????0?11?11211??221??????????1121101????????02???????????????????

*Hylobates* 01002111101011110011111110001011100101111121111101111011111101111011111111111D11100011111000102010001120021001111000000010

*Pongo* 01010110001111100111111111000011111010111031110111111011111111111111211222221212100021110110012111100121020111111010000021

*Gorilla* 10000010011111111111111111100010111011101131111110111111110000011011211222221B12101021112110012111100121020111111011000011

*Pan* 10000010011111011111111111100011111011111031110110111111110000011011211222221B12101021112110012111100121020111111011000011

333333333333333

666777*7*77777788

Taxon/character 789012345678901

*Aegyptopithecus* ?????1?011??112

*Saadanius* ???????????????

*Pliobates* 10100????1??0?1

*Micropithecus* ???????????????

*Dendropithecus* ?????1??????011

*Simiolus* ?????1??????011

*Pliopithecus* ???????????????

*Epipliopithecus* ??100011000?011

*Dionysopithecus* ???????????????

*Barberapithecus* ???????????????

*Anapithecus* ?????????????12

*Plesiopliopithecus* ???????????????

*Crouzelia* ???????????????

*Ekembo* 011001111100001

*Victoriapithecus* ??011??0????101

*Macaca* 101111000000001

*Pierolapithecus* ??????????0?000

*Hispanopithecus* ???????????????

*Hylobates* 111001011000012

*Pongo* 121002011011112

*Gorilla* 121012011011112

*Pan* 121012011011112
