# Supplementary material for: The Miocene primate Pliobates is a pliopithecoid
Source: Nat Commun. 2024 Apr 1;15:2822. doi: 10.1038/s41467-024-47034-9 (PMC10984959; doi:10.1038/s41467-024-47034-9)
Supplement: Supplementary file 9 — Reporting Summary [file 41467_2024_47034_MOESM9_ESM.pdf]

nature portfolio

Corresponding author(s): Florian Bouchet (florian.bouchet@icp.cat), David M. Alba (david.alba@icp.cat)

Last updated by author(s): Dec 13, 2023

Reporting Summary

Nature Portfolio wishes to improve the reproducibility of the work that we publish. This form provides structure for consistency and transparency in reporting. For further information on Nature Portfolio policies, see our Editorial Policies and the Editorial Policy Checklist.

Statistics

For all statistical analyses, confirm that the following items are present in the figure legend, table legend, main text, or Methods section.

n/a	Confirmed	
☐	☒	The exact sample size (*n*) for each experimental group/condition, given as a discrete number and unit of measurement
☒	☐	A statement on whether measurements were taken from distinct samples or whether the same sample was measured repeatedly
☒	☐	The statistical test(s) used AND whether they are one- or two-sided<br>*Only common tests should be described solely by name; describe more complex techniques in the Methods section.*
☒	☐	A description of all covariates tested
☒	☐	A description of any assumptions or corrections, such as tests of normality and adjustment for multiple comparisons
☐	☒	A full description of the statistical parameters including central tendency (e.g. means) or other basic estimates (e.g. regression coefficient) AND variation (e.g. standard deviation) or associated estimates of uncertainty (e.g. confidence intervals)
☒	☐	For null hypothesis testing, the test statistic (e.g. *F*, *t*, *r*) with confidence intervals, effect sizes, degrees of freedom and *P* value noted<br>*Give P values as exact values whenever suitable.*
☒	☐	For Bayesian analysis, information on the choice of priors and Markov chain Monte Carlo settings
☒	☐	For hierarchical and complex designs, identification of the appropriate level for tests and full reporting of outcomes
☒	☐	Estimates of effect sizes (e.g. Cohen's *d*, Pearson's *r*), indicating how they were calculated

Our web collection on statistics for biologists contains articles on many of the points above.

Software and code

Policy information about availability of computer code

Data collection	Dental measurements of most Pliobates cataloniae teeth were taken on the original specimens with a digital caliper, but some teeth were digitally measured in Avizo v.7.0. Dental measurements for the comparative sample were taken from published sources and/or measured from casts by one of us (D.M.A). The µCT scans of P. cataloniae dentognathic specimens were segmented using Avizo v.7.0 (FEI Visualization Sciences Group) to digitally reconstruct the outer enamel and enamel-dentine junction surfaces of the teeth as well as one infant mandible (and digitally extract and reconstruct its preserved teeth).
Data analysis	Body mass estimations and bivariate plots of dental size and proportions were performed in Microsoft Excel 2023. The cladisitic analysis was performed in PAUP*v.4.0a169. Box-and-whisker plots of dental proportions as well as the time-calibrated cladogram were computed in R v.4.1.1.

For manuscripts utilizing custom algorithms or software that are central to the research but not yet described in published literature, software must be made available to editors and reviewers. We strongly encourage code deposition in a community repository (e.g. GitHub). See the Nature Portfolio guidelines for submitting code & software for further information.

Data

Policy information about availability of data

All manuscripts must include a data availability statement. This statement should provide the following information, where applicable:

- Accession codes, unique identifiers, or web links for publicly available datasets
- A description of any restrictions on data availability
- For clinical datasets or third party data, please ensure that the statement adheres to our policy

All dentognathic remains of P. cataloniae, including those from ACM/C5-D1 and the holotype, are housed in the Institut Català de Paleontologia Miquel Crusafont (ICP), Sabadell, Spain, and available to study by other researchers. 3D models of the outer enamel surface (OES) are openly available from MorphoSource, whereas 3D models of the enamel-dentine junction (EDJ) have been uploaded to MorphoSource but are embargoed until ongoing research of tooth endostructural morphology is published. MicroCT scans are curated at the ICP and have also been deposited in MorphoSource. They are accessible upon reasonable request by other researchers, as in the case of the physical fossils housed in the same institution. Most dental measurements for the comparative sample (including both extinct and extant taxa) are available from various published sources. Some dental measurements for extant taxa are available from PRIMO, the NYCEP PRImate Morphology Online database (http://primo.nycep.org). The authors declare that all other data supporting the findings of this study are available within the paper, its supplementary information files, and supplementary data files. Source data are provided as a Source Data file.

Research involving human participants, their data, or biological material

Policy information about studies with human participants or human data. See also policy information about sex, gender (identity/presentation), and sexual orientation and race, ethnicity and racism.

Reporting on sex and gender	N/A
Reporting on race, ethnicity, or other socially relevant groupings	N/A
Population characteristics	N/A
Recruitment	N/A
Ethics oversight	N/A

Note that full information on the approval of the study protocol must also be provided in the manuscript.

Field-specific reporting

Please select the one below that is the best fit for your research. If you are not sure, read the appropriate sections before making your selection.

☐ Life sciences ☐ Behavioural & social sciences ☒ Ecological, evolutionary & environmental sciences

For a reference copy of the document with all sections, see nature.com/documents/nr-reporting-summary-flat.pdf

Ecological, evolutionary & environmental sciences study design

All studies must disclose on these points even when the disclosure is negative.

Study description	The systematic status of Pliobates cataloniae has been subject to debate, originally recoverd as a stem hominoid largely because of its numerous hominoid-like posctranial features or, alternatively, as a pliopithecoid. While many authors consider hominoid postcranial synapomorphies homoplastic, dental morphology provides the main basis in primate taxonomy and is especially diagnostic in pliopithecoids. However P. cataloniae holotype teeth were rather worn and not particularly informative. In this study sixteen additional dentognathic remains of Pliobates cataloniae recovered from a new locality (ACM/C5-D1) adjacent and roughly coeval with the type locality of P. cataloniae (ACM/C8-A4) are presented. These newly reported dental specimens-including lower molars-allow a much better evaluation of Pliobates external tooth morphology, based on which we provide an emended diagnosis and confirm an attribution of this taxon to the superfamily Pliopithecoidea. Dental comparisons with pliopithecoids are performed. We compile a new matrix of dental character including all known pliopithecoid taxa-but not only-which is so far the largest assembled character-taxon matrix for this group. Our cladistic analysis recovers Pliobates cataloniae as a pliopithecoid most closely related to Crouzelia and Plesiopliopithecus, two poorly-known crouzeliid genera from Europe. Further analyses including extant hominoids and using not only dental, but also cranial and postcranial characters, support a pliopithecoid status for Pliobates. Confirming that Pliobates is a pliopithecoid (crouzeliid) shows that locomotor adaptions among stem catarrhines were much more varied than previously thought and has important implications for ape evolution as it supports the repeated evolutionary convergences of the postcranium among various catarrhines lineages, indicating that many of these features might have evolved in parallel between hylobatids and hominids. While most data are qualitative, quantitative data only primarily include tooth measurements and proportions derived from them (i.e., all continuous data) that were not subjected to any type of statistical analysis. The sample of P. cataloniae includes a total of 29 teeth, of which 23 were formally used for comparisons and matrix coding. The sample sizes for the comparative taxa are variable but as many teeth as possible were included for each group, depending upon availability. For each tooth locus the number of teeth included varies between 1 and 15 in the case of extinct species, and can reach more than 100 teeth for extant taxa.

Research sample	We describe the external morphology of sixteen additional dentognathic remains of Pliobates cataloniae from the Abocador de Can Mata locality ACM/C5-D1, also taking into account the two (and only dentognathic) maxillary specimens from the holotype of this species. Three-dimensional virtual imaging is used to reconstruct and figure most P. cataloniae premolars and molars as well as an infant mandible and teeth. Specimens included in the research sample are all those specimens formally attributed to P. cataloniae.
Sampling strategy	Except for the holotype, other specimens of P. cataloniae are reported for the first time. They are relevant in the field of palaeoanthropology, in the context of the debate on Pliobates cataloniae systematic status, as most additional teeth are better preserved than those of the ones and further allow the description of tooth loci previously unknown. No particular sampling procedure was conducted, as all available specimens attributable to P. cataloniae were included in the study. The sample size thus corresponds to all available specimens for this taxon. As most taxa included in the comparative sample are extinct species, the available sample sizes for such taxa are not particularly extensive simple because no further remains are available. The research sample used here is thus adequate in the context of a palaeoanthropological study.
Data collection	The specimens were measured using a digital caliper or digitally measured in Avizo v.7.0. Some of them were scanned by X-ray microtomography. All measures were originally taken by D.M.A but later retaken by F.B. (when the present study started) and repeated at least three times. Scanning procedures were handled by J.F. at the CENIEH (Burgos, Spain). Most 3D models of teeth were segmented from scans by a former ICP technician but verified and/or modified (i.e., dentine reconstructions not previously done) by F.B. and C.Z. The remaining 3D models were segmented by F.B. (with input from C.Z. for dentine reconstructions).
Timing and spatial scale	The scans of the holotype (of better resolution than those of the original study) were performed in March 2018 while those of the ACM/C5-D1 specimens were performed in May 2018. Two scan sessions had to be done for sample and specimen size reasons. The specimens from ACM/C5-D1 were measured between 2020 and 2022: measurements were first taken by D.M.A. in 2020 and retaken by F.B. (with replicates) between 2021 and 2022. All P. cataloniae specimens are from ACM (Spain). The dates of data collection are however irrelevant because they refer to fossil specimens.
Data exclusions	No available data was excluded from this study.
Reproducibility	The methods employed in this work have been previously tested and variously published, and fully demonstrated to be reliable and replicable. All variables are provided in the main text and supplementary information. In particular, dental measurements were repeated several times to ensure value coherence and segmentations were made following standard procedures and tools widely used in palaeoanthropology. The definition and selection of characters in cladistic analysis is observer-dependant but the character states are defined based on concrete morphological descriptions and both the character coding and the cladistic analysis are reproducible by other researchers.
Randomization	No randomization was necessary or is relevant, because this study focuses on a finite and specific sample for which all available specimens/individuals need to be considered.
Blinding	No blinding was necessary in this study since it does not involve any clinical trial with participants and groups.

Did the study involve field work? ☒ Yes ☐ No

Field work, collection and transport

Field conditions	Fieldwork was conducted in the framework of a rescue paleontological intervention during the construction of the Can Mata landfill by professional paleontologists hired to do the work. No environmental conditions were relevant to the study question and species/area investigated since the research sample concern fossil specimens recovered from a single locality.
Location	Abocador de Can Mata, els Hostalets de Pierola, Barcelona, Spain. Lat: 41.52/Long: 1.80. Elevation of the specific fossil locality from which the fossils were recovered is irrelevant and was not recorded. What matters in this regard is the stratigraphic position of the locality relative to the composite stratigraphic sequence of Abocador de Can Mata, which was used to estimate its age based on magnetostratigraphic correlation (reported in the paper following Alba et al., 2017, 2022).
Access & import/export	Paleontological fieldwork was performed under the relevant permits issued on a yearly basis by the regional administration (Culture Department, Generalitat de Catalunya) in accordance with regional and national laws applicable in Spain to paleontological heritage. The fossils are considered cultural heritage and adequately curated at the Museum of the Institut Català de Paleontologia Miquel Crusafont (Sabadell, Spain), which is a public research center. They are accessible to other researchers upon reasonable request for research purposes.
Disturbance	Not applicable because meteorological conditions are irrelevant for paleontological fieldwork, which implies the recovery of fossil remains from sediments, not of samples from living organisms

Reporting for specific materials, systems and methods

We require information from authors about some types of materials, experimental systems and methods used in many studies. Here, indicate whether each material, system or method listed is relevant to your study. If you are not sure if a list item applies to your research, read the appropriate section before selecting a response.

Materials & experimental systems

n/a	Involved in the study
☒	☐ Antibodies
☒	☐ Eukaryotic cell lines
☐	☒ Palaeontology and archaeology
☒	☐ Animals and other organisms
☒	☐ Clinical data
☒	☐ Dual use research of concern
☒	☐ Plants

Methods

n/a	Involved in the study
☒	☐ ChIP-seq
☒	☐ Flow cytometry
☒	☐ MRI-based neuroimaging

Palaeontology and Archaeology

Specimen provenance

All Pliobates cataloniae specimens analyzed in this work come from Abocador de Can Mata (ACM), els Hostalets de Pierola, Catalonia, Spain, from the ACM stratigraphic series (Vallès-Penedès Basin, northeast Iberian Peninsula). The newly reported fossils were recovered in 2008 or subsequently screen-washed from sediments collected during that year during fielwork activities performed by a team of paleontologists hired by the companies PALAEOTHERIA, S.C.P. and FOSSILIA Serveis Paleontològics i Geològics, S.L., under the main direction of J.M.R. Fieldwork permits for 2011 were issued by the Servei d'Arqueologia i Paleontologia of the Culture Department of the Generalitat de Catalunya (permit No. Expedient 437 K121 N 352).

Specimen deposition

All specimens are housed at the Museum of the ICP, Sabadell, Spain.

Dating methods

No new dates are proposed.

☒ Tick this box to confirm that the raw and calibrated dates are available in the paper or in Supplementary Information.

Ethics oversight

No ethical approval or guidance was required because this study focuses on fossil material.

Note that full information on the approval of the study protocol must also be provided in the manuscript.
